# Sex- and Ethnic-Specific Associations of Serum Lipids with Risk of 12 Cancers: Findings from 506,381 Adults in Two Large Cohorts

**DOI:** 10.3390/antiox14091135

**Published:** 2025-09-19

**Authors:** Minh Nguyen Thien, Ji Woo Baek, Yeun Soo Yang, Sun Ha Jee

**Affiliations:** 1 Department of Epidemiology, Faculty of Public Health, University of Medicine and Pharmacy at Ho Chi Minh City, Ho Chi Minh City 17000, Vietnam; minhnt@ump.edu.vn; 2 Department of Epidemiology and Health Promotion, Institute for Health Promotion, Graduate School of Public Health, Yonsei University, Seoul 03772, Republic of Korea; jwbaek0205@yuhs.ac (J.W.B.); or ysyang4647@yuhs.ac (Y.S.Y.)

**Keywords:** serum lipids, cancer risk, neoplasms, cohort studies, ethnicity

## Abstract

The contribution of serum lipids to carcinogenesis, including their effects on inflammation and oxidative stress, remains debated due to inconsistent evidence across populations. This study aimed to elucidate sex- and ethnic-specific associations between serum lipid indices and the risk of 12 common cancers in two large, distinct populations. We conducted a pooled analysis of 506,381 participants from the UK Biobank (UKB) and the Korean Cancer Prevention Study-II (KCPS-II) cohort, with median follow-ups of 12.0 and 13.0 years, respectively. Multivariable-adjusted Cox hazards models were used to estimate hazard ratios (HRs) for the association between baseline lipids and cancer incidence. In the UKB, a one-standard deviation (1-SD) increase in HDL-C was associated with a decreased overall cancer risk (HR 0.982, 95% CI: 0.969–0.995); meanwhile, a 1-SD increase in LDL-C was associated with an increased risk (HR 1.021, 1.009–1.034); higher HDL-C was linked to an increased risk of cervical cancer (HR 1.167, 1.019–1.337) and prostate cancer (HR 1.025, 1.001–1.049). These associations were not significant in the KCPS-II. The association between serum lipids and cancer risk is substantially modified by sex and ethnicity, suggesting that universal lipid-based prevention strategies may be inappropriate and underscoring the need for population-specific research.

## 1. Introduction

Lipids are a class of molecules with a dual role in biological systems. They are indispensable for cellular structure, energy storage, and signal transduction, yet their polyunsaturated fatty acid components render them highly susceptible to oxidative damage. This susceptibility places lipids at the nexus of redox biology and pathology. The peroxidation of lipids, particularly polyunsaturated fatty acids within lipoprotein particles, generates a cascade of reactive aldehydes, including malondialdehyde (MDA) and 4-hydroxynonenal (4-HNE). These electrophilic species can form mutagenic DNA adducts and disrupt cellular signaling, directly implicating lipid-derived oxidative stress in carcinogenesis [1,2].

Within this framework, different lipoprotein fractions can be conceptualized as having opposing roles in systemic redox homeostasis. Low-density lipoprotein (LDL), when modified by reactive oxygen species (ROS), forms oxidized LDL (ox-LDL), a potent pro-inflammatory and pro-oxidant particle that promotes cellular dysfunction [3]. Conversely, high-density lipoprotein (HDL) is classically endowed with significant antioxidant capacity, largely through associated enzymes such as paraoxonase-1 (PON1) and lipid transfer proteins, which can hydrolyze oxidized lipids and prevent the accumulation of ox-LDL [4,5]. However, under conditions of chronic inflammation and high oxidative stress, HDL can become “dysfunctional” losing its protective properties and potentially converting into a pro-inflammatory particle [6].

While these mechanisms have been extensively characterized in vitro and in animal models, their net effect on cancer development at the human population level remains incompletely understood. Epidemiological studies linking circulating serum lipid concentrations to cancer risk have yielded inconsistent results [7,8,9], likely because a single lipid measurement represents an integrated signal influenced by genetics, diet, lifestyle, and comorbidities—all of which modulate the underlying systemic redox environment. This inconsistency strongly suggests the presence of unexamined effect modifiers that fundamentally alter the relationship between systemic lipid levels and cancer development.

This study leverages two large, distinct prospective cohorts-the UK Biobank (UKB) and the Korean Cancer Prevention Study-II (KCPS-II)-to address this gap. These cohorts provide a unique opportunity for direct comparison, as they differ substantially in ethnicity, age structure, lifestyle factors, and baseline cancer incidence patterns. We treated serum lipid profiles as systemic, albeit indirect, indicators of redox balance. We hypothesized that (1) atherogenic lipids (LDL-C, TG, TC, non-HDL-C), as markers of substrate availability for pro-oxidant processes and inflammation, would be positively associated with cancer risk; (2) HDL-C, as a proxy for systemic antioxidant capacity, would be inversely associated with cancer risk; and (3) these associations would be fundamentally modified by population context and sex, reflecting underlying differences in the aggregate burden of oxidative stress and metabolic milieu. By systematically evaluating both serum lipid indices and composite lipid ratios in relation to 12 site-specific cancers, our study aims to clarify the mechanistic and translational implications of lipid metabolism in cancer epidemiology through the lens of redox biology.

## 2. Materials and Methods

### 2.1. Study Design and Study Population

This study utilized data from two extensive prospective cohort studies: the UK Biobank (UKB) and the Korean Cancer Prevention Study-II (KCPS-II). Participants from both cohorts were excluded if they lacked complete information regarding age, sex, height, weight, serum lipid indices, smoking status, alcohol consumption frequency, or physical activity levels. The UK Biobank recruited 502,478 individuals aged 40 to 70 years between 2006 and 2010 from 22 assessment centers across England, Scotland, and Wales. Participants underwent comprehensive physical examinations, provided biological samples (blood, urine, and saliva), and completed detailed health-related questionnaires and interviews [10]. All participants provided electronic informed consent and agreed to be followed up for future health research. After applying the exclusion criteria, the final analytical sample comprised 368,288 participants. Similarly, the KCPS-II collected data from 156,701 individuals who underwent health examinations between 2004 and 2013 at 18 centers across South Korea (15 in Seoul and Gyeonggi Province, and 3 in other provinces). After applying the exclusion criteria, the final analytical sample included 138,093 individuals with complete data [11]. A comprehensive report detailing the excluded cases and the reasons for exclusion can be found in the Supplementary Methods and Figure S1.

### 2.2. Case Ascertainment

In the UKB and KCPS-II, disease information for participants is systematically collected using ICD-10 (International Classification of Diseases, 10th Revision) codes through comprehensive linkages to several national health datasets. These datasets include records from primary care (general practitioners), hospital inpatient and outpatient data, cancer registries, and death registries. The collected data encompasses a wide range of diagnostic and procedural information, such as dates of hospital admissions, diagnoses, procedures, and causes of death. The UK Biobank employs a multi-tiered approach that begins with electronic health records and includes the review of detailed medical records, imaging data, and occasionally biological samples to ensure high-quality data for research purposes [10]. In this study, we classify participants with diabetes, hypertension, cardiovascular diseases, and various types of cancer (including lung cancer, colon cancer, rectal cancer, stomach cancer, liver cancer, bladder cancer, prostate cancer, breast cancer, cervical uteri cancer, thyroid cancer, pancreas cancer, nervous cancer, ovarian cancer) according to ICD-10 codes. All cases diagnosed with cancer within two years from the study enrollment date are excluded from the analysis. Details regarding the ICD-10 codes used for classifying these conditions are provided in Table S1. In addition to the primary outcome of cancer diagnosis throughout the study period, the secondary outcome was the occurrence of early-onset cancer in both cohorts. Early-onset cancer was defined as a cancer diagnosis before the age of 50. In the analysis of early-onset cancer risk, all participants who entered the cohort after the age of 50 were excluded. Additionally, any cases of cancer diagnosed within two years from the start of follow-up were also excluded.

### 2.3. Assessment of Serum Lipid Indices and Serum Lipid Ratios

In the UK Biobank, approximately 45 mL of blood is collected from each participant using vacutainer tubes containing specific anticoagulants or clot activators suitable for subsequent analyses. At the central laboratory, the blood samples are centrifuged to separate serum and plasma, which are then aliquoted into cryostorage tubes. These aliquots are stored in two archives: an automated system at −80 °C and a manual liquid nitrogen system at −180 °C, ensuring stability and future use [12]. Serum samples for lipid analysis, including low-density lipoprotein cholesterol (LDL-C), high-density lipoprotein cholesterol (HDL-C), triglycerides (TG), and total cholesterol (TC), are analyzed using high-throughput clinical chemistry analyzers. Serum lipid levels are measured in millimoles per liter (mmol/L), with enzymatic methods used for TG and TC, while LDL-C and HDL-C levels are either directly measured or calculated using standard laboratory methods. Serum lipid ratios are calculated from serum lipid indices, including non-HDL-C, triglycerides/HDL-C ratio, atherogenic index of plasma (AIP), Castelli’s index-I (CRI-I), Castelli’s index-II (CRI-II), lipoprotein combination index (LCI), and the atherogenic coefficient (AC). The formulas for calculating serum lipid ratios are detailed in Table S2.

### 2.4. Covariables

Serum lipid indices and lipid ratios were categorized into quintiles for analysis within subgroups defined by sex, body mass index (BMI), cardiovascular disease (CVD), type 2 diabetes mellitus (T2DM), and hypertension. We assessed the risk of various cancer types in relation to serum lipid indices across the entire population, followed by separate analyses for males and females. Subsequently, stratified analyses were conducted by risk factors, including T2DM, hypertension, CVD, age, smoking history, alcohol consumption habits, and physical activity frequency, with separate assessments for males and females. BMI was categorized into groups based on different cut-off points adjusted for racial variation. Dyslipidaemia was defined as the presence of any of the following conditions: TC ≥ 200 mg/dL (5.18 mmol/L), TG ≥ 150 mg/dL (1.7 mmol/L), LDL-C ≥ 130 mg/dL (3.37 mmol/L), or HDL-C < 40 mg/dL (1.03 mmol/L) for men and HDL-C < 50 mg/dL (1.29 mmol/L) for women. Details of the cut-off points are provided in the Supplementary Methods.

### 2.5. Statistical Analysis

Descriptive statistics (means with SD for continuous variables; frequencies and percentages for categorical variables) were used to summarize baseline characteristics of the cohorts. Incidence rates per 100,000 person-years were calculated for each cancer type, stratified by sex and cohort. Cox proportional hazards models were employed to estimate hazard ratios (HRs) and 95% confidence intervals (CIs). For the association between serum lipids and cancer risk, each serum lipid index and ratio was modeled as a continuous variable, standardized to a mean of 0 and a standard deviation (SD) of 1, to report the risk associated with a 1-SD increase. Analyses were conducted for each cohort separately and stratified by sex. The final multivariable model (Model 3) was adjusted for age (as the time scale), BMI, smoking status, alcohol consumption, physical activity level, and baseline prevalence of type 2 diabetes and hypertension. For sex-specific cancers, analyses were restricted to the relevant sex. For comparing cancer incidence between cohorts, the cox models were used to estimate HRs for cancer incidence in UKB participants compared to KCPS-II participants. In these models, the cohort indicator (UKB vs. KCPS-II, with KCPS-II as the reference) was the primary exposure variable. Model 1 was adjusted for age and sex. Model 2 further adjusted for BMI, dyslipidemia, hypertension, type 2 diabetes mellitus, and cardiovascular disease (CVD). Model 3 included additional adjustments for smoking status, alcohol consumption, and physical activity frequency. To formally test for effect modification, an interaction term between the standardized lipid variable and a binary cohort indicator (UKB vs. KCPS-II) was included in a pooled model. Sensitivity analyses were conducted by excluding cancer cases diagnosed within the first two years of follow-up to further address the potential for reverse causality. We also calculated E-values for the associations. The E-value quantifies the minimum strength of association, on the hazard ratio scale, that an unmeasured confounder would need to have with both the exposure (cohort) and the outcome (cancer) to fully explain away the observed association, conditional on the measured covariates. A larger E-value indicates greater robustness of the observed association to unmeasured confounding. All statistical analyses and data presentations were conducted using R version 4.3.3 and relevant R packages (Supplementary Table S6). A two-sided *p*-value of less than 0.05 was considered statistically significant.

## 3. Results

### 3.1. Study Population and Cancer Incidence

We analyzed 506,381 adults from two cohorts: 368,288 participants in the UK Biobank (UKB; 196,739 women; 171,549 men) and 138,093 in the Korean Cancer Prevention Study-II (KCPS-II; 51,949 women; 86,144 men) (Table 1). During follow-up, overall cancer occurred in 27,457 women (14.0%) and 28,052 men (16.4%) in UKB, and in 4024 women (7.8%) and 5356 men (6.2%) in KCPS-II. When overall cancer was defined after excluding breast and/or thyroid cancer, incident cases were 20,942 (10.6%) in UKB women, 27,955 (16.3%) in UKB men, 1931 (3.7%) in KCPS-II women, and 4460 (5.2%) in KCPS-II men (Supplementary Table S3).

**Table 1:** Demographic characteristics of study participants.

Characteristics	UK Biobank (n = 368,288)		KCPS-II Biobank (n = 138,093)	
	Women (n = 196,739)	Men (n = 171,549)	Women (n = 51,949)	Men (n = 86,144)
Mean Age (SD), years	56.7 (8.00)	57.0 (8.19)	39.9 (10.9)	41.5 (9.55)
Age groups				
Under 40 years old	73 (0.04%)	79 (0.05%)	28,610 (55.1%)	41,660 (48.4%)
40 to 50 years old	47,898 (24.3%)	41,167 (24.0%)	13,318 (25.6%)	27,545 (32.0%)
50 to 60 years old	68,474 (34.8%)	55,751 (32.5%)	7018 (13.5%)	12,655 (14.7%)
Over 60 years old	80,294 (40.8%)	74,552 (43.5%)	2578 (4.96%)	3593 (4.17%)
Mean BMI (SD), kg/m^2^	27.0 (5.11)	27.8 (4.19)	22.1 (3.08)	24.4 (2.90)
BMI groups				
Underweight	1501 (0.76%)	373 (0.22%)	4899 (9.44%)	1182 (1.37%)
Normal weight	78,172 (39.7%)	42,926 (25.0%)	29,843 (57.5%)	25,990 (30.2%)
Overweight	72,281 (36.7%)	85,364 (49.8%)	13,627 (26.2%)	44,396 (51.6%)
Obesity	44,785 (22.8%)	42,886 (25.0%)	3550 (6.84%)	14,538 (16.9%)
Type 2 Diabetes mellitus				
No	186,566 (94.8%)	155,564 (90.7%)	50,567 (97.3%)	81,105 (94.2%)
Yes	10,173 (5.17%)	15,985 (9.32%)	1382 (2.66%)	5039 (5.85%)
Hypertension				
No	149,636 (76.1%)	116,579 (68.0%)	46,710 (89.9%)	68,632 (79.7%)
Yes	47,103 (23.9%)	54,970 (32.0%)	5239 (10.1%)	17,512 (20.3%)
Cardiovascular diseases				
No	181,038 (92.0%)	142,265 (82.9%)	51,685 (99.5%)	85,726 (99.5%)
Yes	15,701 (7.98%)	29,284 (17.1%)	264 (0.51%)	418 (0.49%)
Smoking status				
Never	117,465 (59.7%)	84,861 (49.5%)	46,543 (89.6%)	19,725 (22.9%)
Former	62,125 (31.6%)	65,835 (38.4%)	3232 (6.22%)	27,943 (32.4%)
Current	17,149 (8.72%)	20,853 (12.2%)	2174 (4.18%)	38,476 (44.7%)
Alcohol consumption				
Under 1 times/week	124,879 (63.5%)	134,173 (78.2%)	16,178 (31.1%)	5056 (5.87%)
1 to 2 times/week	25,789 (13.1%)	15,230 (8.88%)	8653 (16.7%)	7109 (8.25%)
Over 2 times/week	46,071 (23.4%)	22,146 (12.9%)	27,118 (52.2%)	73,979 (85.9%)
Physical activity				
Under 2 times/week	39,535 (20.1%)	37,056 (21.6%)	14,267 (43.9%)	37,870 (56.4%)
2 to 4 times/week	81,373 (41.4%)	65,276 (38.1%)	12,931 (39.8%)	21,706 (32.3%)
Over 4 times/week	75,831 (38.5%)	69,217 (40.3%)	5279 (16.3%)	7571 (11.3%)
Mean Systolic blood pressure (SD), mmHg	137 (20.2)	143 (18.4)	112 (14.2)	121 (13.0)
Mean Diastolic blood pressure (SD), mmHg	80.6 (10.5)	84.1 (10.5)	70.1 (9.65)	76.5 (9.63)
Mean FBS, (SD), mmol/L	5.05 (1.04)	5.17 (1.35)	4.86 (0.85)	5.16 (1.12)
Mean HDL-C (SD), mmol/L	1.60 (0.38)	1.28 (0.31)	1.49 (0.29)	1.26 (0.24)
Mean LDL-C (SD), mmol/L	3.63 (0.87)	3.50 (0.86)	2.76 (0.78)	2.98 (0.81)
Mean TG (SD), mmol/L	1.54 (0.85)	1.97 (1.13)	1.10 (0.66)	1.78 (1.12)
Mean TC (SD), mmol/L	5.88 (1.12)	5.50 (1.12)	4.74 (0.85)	4.98 (0.85)
Mean non-HDL-C (SD)	4.28 (1.07)	4.22 (1.07)	3.25 (0.88)	3.71 (0.90)
Mean AIP (SD)	−0.14 (0.62)	0.32 (0.67)	−0.40 (0.56)	0.21 (0.63)
Mean AC (SD)	2.83 (1.00)	3.46 (1.15)	2.30 (1.09)	3.09 (1.18)
Mean CRI-I (SD)	3.83 (1.00)	4.46 (1.15)	3.30 (1.09)	4.09 (1.18)
Mean CRI-II (SD)	2.39 (0.78)	2.85 (0.87)	1.94 (0.93)	2.47 (0.98)
Mean LCI (SD)	25.2 (25.3)	35.6 (33.3)	22.0 (15.2)	25.0 (28.7)
Mean THDL (SD)	1.07 (0.80)	1.71 (1.27)	0.80 (0.64)	1.52 (1.18)

Note: FBS: Fasting blood sugar; HDL-C: High-density lipoprotein Cholesterol; LDL-C: Low-density lipoprotein Cholesterol; TG: Triglycerides; TC: Total Cholesterol; AIP: Atherogenic Index of plasma; AC: Atherogenic coefficient; CRI-I: Castelli risk index -I; CRI-II: Castelli risk index-II; LCI: Lipoprotein combination index; THDL: Triglyceride HDL-C ratio; SD: Standard deviation; BMI: Body mass index; UK: United Kingdom; KCPS-II: Korean Cancer Prevention Study-II.

### 3.2. Overall Cancer

Across cohorts, overall cancer incidence (per 100,000 person-years) was higher in the UK Biobank (UKB) than in KCPS-II and showed an opposite pattern by sex between the cohorts: in UKB, men exceeded women (1472.89 vs. 1238.28), whereas in KCPS-II, women exceeded men (572.09 vs. 456.38) (Table 2). Site-specific patterns mirrored established geographic epidemiology: colon, bladder, lung, prostate, breast, and pancreatic cancer rates were substantially higher in UKB than KCPS-II (e.g., colon: 111.06 vs. 23.92 in men; prostate: 424.80 vs. 53.32 in men; breast: 437.16 vs. 130.08 in women), while stomach and thyroid cancer rates were markedly higher in KCPS-II (e.g., stomach: 73.70 vs. 26.25 in men; thyroid: 168.47 vs. 12.82 in women). Early-onset overall cancer was more frequent in women in both cohorts and notably higher in KCPS-II women than UKB women (458.45 vs. 389.17 per 100,000 person-years), driven in part by elevated early-onset thyroid cancer in KCPS-II. These contrasts underscore substantial population and sex heterogeneity in background cancer risk that is essential for interpreting lipid–cancer associations.

In UKB women, higher atherogenic lipids were associated with higher overall cancer risk, whereas HDL-C was inversely associated. Per 1-SD increase, the adjusted hazard ratios (aHRs) were as follows: LDL-C 1.021 (95% CI 1.009–1.034; *p* < 0.001), TG 1.020 (1.007–1.032; *p* = 0.0015), TC 1.016 (1.004–1.029; *p* = 0.008), and non-HDL-C 1.022 (1.010–1.035; *p* < 0.001); HDL-C 0.982 (0.969–0.995; *p* = 0.008). Atherogenic ratios were also positively associated (e.g., AIP 1.029 [1.015–1.043], AC/CRI-I 1.031 [1.019–1.044], CRI-II 1.032 [1.019–1.044]; all *p* < 0.001). These patterns were similar or modestly stronger when breast and/or thyroid cancers were excluded from the “overall cancer” outcome in UKB women: HDL-C 0.974 (0.959–0.990; *p* < 0.001), LDL-C 1.024 (1.010–1.038; *p* < 0.001), TG 1.023 (1.009–1.037; *p* = 0.001), TC 1.018 (1.004–1.032; *p* = 0.012), non-HDL-C 1.026 (1.012–1.040; *p* < 0.001), and AIP 1.035 (1.019–1.050; *p* < 0.001). In UKB men, HDL-C and TG were not associated with overall cancer, but LDL-C, TC, and non-HDL-C showed consistent positive associations per 1-SD: LDL-C 1.028 (1.016–1.041; *p* < 0.001), TC 1.028 (1.016–1.040; *p* < 0.001), and non-HDL-C 1.028 (1.015–1.040; *p* < 0.001). Atherogenic ratios (AC/CRI-I/CRI-II) were similarly positive (e.g., CRI-II 1.027 [1.014–1.040]; *p* < 0.001). Excluding breast/thyroid cancers yielded nearly identical estimates.

In KCPS-II, per-SD lipid associations with overall cancer were generally null in both women and men, with effect sizes near unity (e.g., women: LDL-C 1.005 [0.966–1.046]; men: LDL-C 1.008 [0.977–1.039]). Forest plots summarizing per-SD effects by sex and cohort are provided in Table 3 (women) and Table 4 (men).

### 3.3. Cancer Site–Specific Associations

For women in UK Biobank, higher TG and TG-based ratios were associated with a higher risk of lung cancer: per 1-SD TG 1.060 (1.015–1.107; *p* = 0.008), AIP 1.110 (1.053–1.170; *p* < 0.001), and TG/HDL-C 1.055 (1.013–1.099; *p* = 0.010). LDL-C and TC were not associated. For colon cancer, TG and composite indices were positively associated (TG 1.053 [1.010–1.098]; *p* = 0.016; TC 1.048 [1.001–1.097]; *p* = 0.046; LCI 1.049 [1.009–1.090]; *p* = 0.017), whereas HDL-C was not. Rectal cancer showed no consistent per-SD associations. For stomach cancer, HDL-C was inversely associated (per 1-SD 0.812 [0.693–0.952]; *p* = 0.010) with other lipids and the ratios were largely null. For breast cancer, several atherogenic markers were positively associated: LDL-C 1.027 (1.007–1.048; *p* = 0.009), TG 1.025 (1.005–1.046; *p* = 0.015), TC 1.027 (1.006–1.048; *p* = 0.010), non-HDL-C 1.027 (1.007–1.048; *p* = 0.008), and LCI 1.033 (1.014–1.053; *p* < 0.001). HDL-C was not associated. For cervix uteri cancer, HDL-C was positively associated (1.167 [1.019–1.337]; *p* = 0.025), while other lipids were not consistently related. Thyroid and pancreatic cancers showed no material per-SD associations. For ovarian cancer, TC showed a modest positive association (1.069 [1.001–1.141]; *p* = 0.046).

For Men in UK Biobank, LDL-C and non-HDL-C were inversely associated with the risk of lung cancer (LDL-C 0.948 [0.902–0.996]; *p* = 0.034; non-HDL-C 0.951 [0.905–0.999]; *p* = 0.045), whereas other lipids were null. For colorectal cancer, atherogenic lipids were positively associated—more strongly for colon than rectum. Colon cancer: LDL-C 1.072 (1.027–1.119; *p* = 0.001), TG 1.061 (1.017–1.106; *p* = 0.006), TC 1.079 (1.034–1.126; *p* < 0.001), and non-HDL-C 1.085 (1.039–1.132; *p* < 0.001). Rectal cancer: LDL-C 1.095 (1.030–1.165; *p* = 0.004), TC 1.112 (1.046–1.183; *p* < 0.001), non-HDL-C 1.093 (1.028–1.162; *p* = 0.005); notably, HDL-C was also positively associated (1.087 [1.018–1.160]; *p* = 0.012). For liver cancer, we observed robust inverse associations across multiple atherogenic measures: LDL-C 0.807 (0.718–0.906; *p* < 0.001), TC 0.833 (0.741–0.937; *p* = 0.002), non-HDL-C 0.813 (0.723–0.915; *p* < 0.001), AC/CRI-I 0.829 (0.736–0.934; *p* = 0.002), and CRI-II 0.813 (0.725–0.912; *p* < 0.001). For bladder cancer, higher LDL-C, non-HDL-C, and related ratios were associated with higher risk (e.g., LDL-C 1.057 [1.011–1.106]; *p* = 0.016; non-HDL-C 1.055 [1.008–1.103]; *p* = 0.020; CRI-II 1.075 [1.026–1.126]; *p* = 0.002). For prostate cancer, atherogenic indices were consistently positive: LDL-C 1.056 (1.034–1.080; *p* < 0.001), TC 1.056 (1.034–1.080; *p* < 0.001), non-HDL-C 1.052 (1.029–1.075; *p* < 0.001), and CRI-II 1.033 (1.010–1.057; *p* = 0.005). For thyroid cancer, TG-based markers were positively associated (TG 1.176 [1.008–1.373]; *p* = 0.040; TG/HDL-C 1.198 [1.050–1.367]; *p* = 0.007; AIP 1.277 [1.038–1.572]; *p* = 0.021), while HDL-C suggested an inverse trend (0.778 [0.604–1.001]; *p* = 0.051). Pancreatic cancer showed no material per-SD associations.

**Table 3:** Hazard ratio and 95% CI between serum lipids and cancer risk in women, when 1 SD increases the serum lipid indexes or serum lipid ratios.

Cancer/Cohort	HDL-C	LDL-C	TG	TC	Non-HDL-C	AIP	AC	CRI-I	CRI-II	LCI	THDL
Overall cancer											
UKB	0.982 (0.969–0.995)	1.021 (1.009–1.034)	1.020 (1.007–1.032)	1.016 (1.004–1.029)	1.022 (1.010–1.035)	1.029 (1.015–1.043)	1.031 (1.019–1.044)	1.031 (1.019–1.044)	1.032 (1.019–1.044)	1.023 (1.012–1.035)	1.022 (1.009–1.034)
KCPS-II	0.974 (0.934–1.015)	1.005 (0.966–1.046)	0.992 (0.956–1.030)	0.990 (0.951–1.030)	0.999 (0.960–1.039)	1.018 (0.977–1.061)	1.018 (0.974–1.063)	1.018 (0.974–1.063)	1.023 (0.979–1.070)	0.986 (0.946–1.029)	1.003 (0.967–1.041)
Overall cancer *											
UKB	0.974 (0.959–0.990)	1.024 (1.010–1.038)	1.023 (1.009–1.037)	1.018 (1.004–1.032)	1.026 (1.012–1.040)	1.035 (1.019–1.050)	1.038 (1.024–1.053)	1.038 (1.024–1.053)	1.038 (1.024–1.053)	1.025 (1.012–1.038)	1.025 (1.012–1.039)
KCPS-II	0.996 (0.938–1.057)	1.023 (0.968–1.082)	0.979 (0.931–1.029)	1.006 (0.951–1.065)	1.008 (0.952–1.067)	1.009 (0.954–1.069)	1.027 (0.964–1.093)	1.027 (0.964–1.093)	1.041 (0.977–1.109)	1.007 (0.953–1.065)	0.991 (0.943–1.042)
Lung cancer											
UKB	0.912 (0.861–0.966)	0.976 (0.930–1.023)	1.060 (1.015–1.107)	0.967 (0.922–1.015)	0.990 (0.945–1.038)	1.110 (1.053–1.170)	1.045 (0.995–1.096)	1.045 (0.995–1.096)	1.037 (0.987–1.089)	1.020 (0.980–1.061)	1.055 (1.013–1.099)
KCPS-II	0.999 (0.815–1.226)	1.068 (0.912–1.250)	0.985 (0.807–1.204)	1.045 (0.884–1.234)	1.044 (0.885–1.231)	0.967 (0.790–1.185)	1.049 (0.875–1.257)	1.049 (0.875–1.257)	1.074 (0.900–1.280)	1.095 (0.944–1.270)	1.024 (0.864–1.213)
Colon cancer											
UKB	1.012 (0.961–1.066)	1.046 (0.999–1.095)	1.053 (1.010–1.098)	1.048 (1.001–1.097)	1.046 (0.999–1.095)	1.045 (0.993–1.099)	1.031 (0.984–1.081)	1.031 (0.984–1.081)	1.032 (0.985–1.083)	1.049 (1.009–1.090)	1.039 (0.997–1.084)
KCPS-II	0.975 (0.799–1.190)	1.114 (0.891–1.393)	1.015 (0.888–1.159)	1.095 (0.918–1.305)	1.101 (0.930–1.303)	1.071 (0.883–1.298)	1.063 (0.883–1.279)	1.063 (0.883–1.279)	1.080 (0.884–1.320)	1.012 (0.896–1.143)	1.004 (0.867–1.161)
Rectal cancer											
UKB	1.064 (0.980–1.156)	1.033 (0.956–1.117)	1.033 (0.956–1.116)	1.058 (0.979–1.144)	1.041 (0.964–1.125)	1.008 (0.924–1.099)	0.981 (0.905–1.063)	0.981 (0.905–1.063)	0.972 (0.897–1.053)	0.998 (0.930–1.070)	1.011 (0.929–1.099)
KCPS-II	0.934 (0.730–1.194)	1.124 (0.844–1.497)	1.036 (0.866–1.239)	1.075 (0.845–1.367)	1.096 (0.870–1.383)	1.018 (0.773–1.341)	1.126 (0.858–1.480)	1.126 (0.858–1.480)	1.152 (0.852–1.557)	1.041 (0.889–1.219)	1.034 (0.873–1.224)
Stomach cancer											
UKB	0.812 (0.693–0.952)	0.969 (0.863–1.088)	0.958 (0.851–1.078)	0.930 (0.824–1.051)	0.979 (0.872–1.098)	1.077 (0.937–1.239)	1.081 (0.962–1.216)	1.081 (0.962–1.216)	1.084 (0.963–1.221)	0.947 (0.849–1.055)	0.999 (0.898–1.111)
KCPS-II	0.883 (0.739–1.055)	1.046 (0.895–1.221)	1.027 (0.929–1.136)	0.974 (0.830–1.142)	1.014 (0.869–1.182)	1.116 (0.955–1.304)	1.066 (0.911–1.248)	1.066 (0.911–1.248)	1.090 (0.933–1.273)	1.017 (0.915–1.130)	1.044 (0.949–1.147)
Liver cancer											
UKB	0.978 (0.830–1.152)	0.945 (0.829–1.078)	1.055 (0.929–1.198)	0.957 (0.836–1.095)	0.961 (0.841–1.098)	1.092 (0.941–1.268)	1.024 (0.883–1.188)	1.024 (0.883–1.188)	1.006 (0.869–1.165)	1.030 (0.904–1.173)	1.090 (0.964–1.232)
KCPS-II	1.156 (0.739–1.809)	1.002 (0.654–1.536)	0.854 (0.655–1.113)	1.046 (0.684–1.599)	0.995 (0.651–1.523)	0.922 (0.665–1.278)	1.008 (0.630–1.615)	1.008 (0.630–1.615)	1.029 (0.620–1.709)	0.936 (0.657–1.334)	0.869 (0.655–1.154)
Breast cancer											
UKB	1.000 (0.979–1.023)	1.027 (1.007–1.048)	1.025 (1.005–1.046)	1.027 (1.006–1.048)	1.027 (1.007–1.048)	1.027 (1.004–1.049)	1.026 (1.004–1.047)	1.026 (1.004–1.047)	1.026 (1.005–1.048)	1.033 (1.014–1.053)	1.021 (1.001–1.042)
KCPS-II	1.004 (0.924–1.092)	1.030 (0.948–1.119)	1.009 (0.931–1.093)	1.028 (0.949–1.114)	1.026 (0.946–1.113)	1.017 (0.929–1.112)	1.024 (0.933–1.123)	1.024 (0.933–1.123)	1.026 (0.934–1.127)	0.993 (0.917–1.077)	1.016 (0.935–1.105)
Cervix uteri cancer											
UKB	1.167 (1.019–1.337)	1.007 (0.890–1.140)	1.040 (0.922–1.172)	1.066 (0.947–1.201)	1.020 (0.902–1.154)	0.980 (0.854–1.125)	0.955 (0.834–1.093)	0.955 (0.834–1.093)	0.942 (0.822–1.080)	1.008 (0.906–1.121)	0.979 (0.867–1.105)
KCPS-II	0.970 (0.670–1.404)	1.524 (1.103–2.105)	1.101 (1.003–1.209)	1.190 (1.062–1.334)	1.197 (1.067–1.344)	1.279 (0.923–1.774)	1.261 (1.076–1.478)	1.261 (1.076–1.478)	1.302 (1.094–1.549)	1.126 (1.041–1.217)	1.112 (1.011–1.224)
Thyroid cancer											
UKB	0.878 (0.769–1.002)	1.015 (0.903–1.141)	1.054 (0.944–1.176)	1.002 (0.888–1.131)	1.038 (0.923–1.167)	1.093 (0.967–1.236)	1.083 (0.969–1.210)	1.083 (0.969–1.210)	1.069 (0.958–1.194)	1.062 (0.955–1.182)	1.069 (0.963–1.186)
KCPS-II	0.900 (0.833–0.972)	0.962 (0.893–1.037)	1.014 (0.942–1.091)	0.933 (0.865–1.007)	0.968 (0.898–1.043)	1.047 (0.970–1.131)	1.008 (0.935–1.087)	1.008 (0.935–1.087)	1.002 (0.925–1.086)	0.939 (0.867–1.017)	1.025 (0.961–1.094)
Pancreas cancer											
UKB	1.052 (0.953–1.161)	1.050 (0.962–1.146)	1.001 (0.913–1.099)	1.061 (0.972–1.158)	1.049 (0.962–1.143)	0.996 (0.902–1.100)	1.013 (0.924–1.111)	1.013 (0.924–1.111)	1.012 (0.923–1.110)	1.017 (0.930–1.112)	0.990 (0.900–1.090)
KCPS-II	1.022 (0.688–1.517)	0.948 (0.637–1.410)	1.067 (0.934–1.219)	1.092 (0.715–1.667)	1.083 (0.708–1.656)	1.134 (0.818–1.572)	1.092 (0.716–1.665)	1.092 (0.716–1.665)	0.981 (0.625–1.539)	1.100 (0.935–1.294)	1.074 (0.897–1.287)
Ovarian cancer											
UKB	1.018 (0.953–1.088)	1.063 (0.996–1.135)	1.026 (0.964–1.091)	1.069 (1.001–1.141)	1.066 (0.998–1.138)	1.018 (0.950–1.091)	1.037 (0.971–1.107)	1.037 (0.971–1.107)	1.034 (0.969–1.103)	1.049 (0.990–1.111)	1.015 (0.953–1.082)
KCPS-II	1.047 (0.803–1.365)	1.130 (0.853–1.497)	0.993 (0.729–1.352)	1.107 (0.907–1.353)	1.094 (0.862–1.388)	0.924 (0.667–1.280)	1.095 (0.854–1.406)	1.095 (0.854–1.406)	1.120 (0.898–1.397)	1.098 (0.972–1.239)	1.027 (0.755–1.395)

Note: *: Overall cancer excluded participants of breast cancer and thyroid cancer. HDL: High-Density Lipoprotein Cholesterol; LDL: Low-density lipoprotein Cholesterol; TG: Triglycerides; TC: Total Cholesterol; Non HDL-C: Non-high-density lipoprotein Cholesterol; AIP: Atherogenic Index of plasma; AC: Atherogenic coefficient; CRI1: Castelli risk index -I; CRI2: Castelli risk index-II; LCI: Lipoprotein combination index; THDL: Triglyceride HDL-C ratio.

**Table 4:** Hazard ratio and 95% CI between serum lipids and cancer risk in men, when 1 SD increases the serum lipid indexes or serum lipid ratios.

Cancer/Cohort	HDL-C	LDL-C	TG	TC	Non-HDL-C	AIP	AC	CRI-I	CRI-II	LCI	THDL
Overall cancer											
UKB	1.006 (0.993–1.019)	1.028 (1.016–1.041)	0.999 (0.987–1.012)	1.028 (1.016–1.040)	1.028 (1.015–1.040)	1.001 (0.988–1.014)	1.026 (1.013–1.039)	1.026 (1.013–1.039)	1.027 (1.014–1.040)	1.011 (0.998–1.023)	1.001 (0.988–1.013)
KCPS-II	1.017 (0.987–1.049)	1.008 (0.977–1.039)	0.982 (0.950–1.015)	1.008 (0.978–1.040)	1.003 (0.972–1.035)	0.984 (0.951–1.017)	0.991 (0.960–1.023)	0.991 (0.960–1.023)	0.995 (0.965–1.027)	0.987 (0.951–1.024)	0.981 (0.949–1.016)
Overall cancer *											
UKB	1.006 (0.993–1.019)	1.029 (1.016–1.041)	1.000 (0.987–1.012)	1.028 (1.016–1.041)	1.028 (1.016–1.040)	1.001 (0.988–1.014)	1.026 (1.013–1.039)	1.026 (1.013–1.039)	1.027 (1.014–1.040)	1.011 (0.999–1.024)	1.001 (0.989–1.014)
KCPS-II	1.026 (0.993–1.060)	1.013 (0.979–1.048)	0.969 (0.934–1.006)	1.014 (0.980–1.050)	1.006 (0.972–1.042)	0.969 (0.933–1.005)	0.989 (0.954–1.024)	0.989 (0.954–1.024)	0.995 (0.961–1.030)	0.978 (0.938–1.019)	0.969 (0.932–1.007)
Lung cancer											
UKB	1.035 (0.980–1.092)	0.948 (0.902–0.996)	0.999 (0.950–1.051)	0.961 (0.914–1.009)	0.951 (0.905–0.999)	0.987 (0.935–1.042)	0.961 (0.913–1.012)	0.961 (0.913–1.012)	0.958 (0.909–1.008)	0.976 (0.926–1.028)	0.995 (0.947–1.045)
KCPS-II	1.058 (0.970–1.154)	1.047 (0.943–1.162)	0.984 (0.874–1.109)	1.047 (0.943–1.162)	1.026 (0.925–1.139)	0.958 (0.854–1.075)	0.961 (0.861–1.071)	0.961 (0.861–1.071)	0.979 (0.880–1.090)	0.977 (0.855–1.115)	0.962 (0.850–1.088)
Colon cancer											
UKB	0.991 (0.945–1.038)	1.072 (1.027–1.119)	1.061 (1.017–1.106)	1.079 (1.034–1.126)	1.085 (1.039–1.132)	1.046 (0.999–1.097)	1.082 (1.036–1.130)	1.082 (1.036–1.130)	1.074 (1.028–1.122)	1.077 (1.036–1.119)	1.060 (1.018–1.103)
KCPS-II	0.989 (0.868–1.127)	1.150 (1.006–1.314)	1.053 (0.928–1.194)	1.200 (1.057–1.362)	1.192 (1.048–1.357)	1.088 (0.951–1.246)	1.142 (1.012–1.290)	1.142 (1.012–1.290)	1.132 (0.998–1.283)	1.068 (0.989–1.153)	1.034 (0.918–1.165)
Rectal cancer											
UKB	1.087 (1.018–1.160)	1.095 (1.030–1.165)	1.022 (0.958–1.089)	1.112 (1.046–1.183)	1.093 (1.028–1.162)	0.985 (0.922–1.053)	1.037 (0.972–1.106)	1.037 (0.972–1.106)	1.036 (0.972–1.104)	1.057 (0.993–1.124)	1.006 (0.942–1.075)
KCPS-II	1.032 (0.908–1.172)	0.951 (0.819–1.105)	1.068 (0.939–1.214)	1.007 (0.868–1.168)	0.998 (0.858–1.162)	1.068 (0.913–1.248)	0.962 (0.825–1.121)	0.962 (0.825–1.121)	0.930 (0.794–1.089)	0.986 (0.863–1.126)	1.031 (0.906–1.173)
Stomach cancer											
UKB	0.991 (0.892–1.101)	0.973 (0.897–1.056)	1.003 (0.924–1.089)	0.973 (0.896–1.056)	0.974 (0.899–1.055)	1.023 (0.932–1.123)	1.006 (0.925–1.093)	1.006 (0.925–1.093)	1.002 (0.922–1.090)	0.958 (0.884–1.038)	1.023 (0.943–1.110)
KCPS-II	0.948 (0.877–1.025)	1.043 (0.965–1.128)	1.051 (0.978–1.129)	1.061 (0.982–1.148)	1.075 (0.995–1.161)	1.061 (0.977–1.152)	1.071 (0.998–1.150)	1.071 (0.998–1.150)	1.054 (0.980–1.133)	1.030 (0.973–1.090)	1.052 (0.984–1.124)
Liver cancer											
UKB	1.061 (0.945–1.190)	0.807 (0.718–0.906)	0.980 (0.880–1.091)	0.833 (0.741–0.937)	0.813 (0.723–0.915)	0.955 (0.855–1.067)	0.829 (0.736–0.934)	0.829 (0.736–0.934)	0.813 (0.725–0.912)	0.894 (0.784–1.020)	0.988 (0.889–1.098)
KCPS-II	1.082 (0.959–1.221)	0.860 (0.759–0.975)	0.626 (0.477–0.820)	0.765 (0.673–0.869)	0.747 (0.649–0.861)	0.650 (0.553–0.763)	0.818 (0.680–0.983)	0.818 (0.680–0.983)	0.876 (0.757–1.013)	0.694 (0.494–0.976)	0.724 (0.518–1.012)
Bladder cancer											
UKB	0.957 (0.910–1.007)	1.057 (1.011–1.106)	1.019 (0.973–1.067)	1.042 (0.995–1.090)	1.055 (1.008–1.103)	1.043 (0.994–1.095)	1.066 (1.018–1.116)	1.066 (1.018–1.116)	1.075 (1.026–1.126)	1.027 (0.982–1.074)	1.011 (0.968–1.057)
KCPS-II	0.921 (0.787–1.077)	1.032 (0.855–1.247)	1.125 (0.977–1.295)	1.089 (0.903–1.312)	1.113 (0.925–1.339)	1.184 (0.963–1.455)	1.066 (0.907–1.252)	1.066 (0.907–1.252)	1.018 (0.857–1.208)	1.033 (0.909–1.174)	1.096 (0.964–1.246)
Prostate cancer											
UKB	1.025 (1.001–1.049)	1.056 (1.034–1.080)	0.986 (0.963–1.010)	1.056 (1.034–1.080)	1.052 (1.029–1.075)	0.980 (0.957–1.004)	1.028 (1.005–1.052)	1.028 (1.005–1.052)	1.033 (1.010–1.057)	1.014 (0.990–1.038)	0.978 (0.954–1.003)
KCPS-II	1.054 (0.972–1.143)	1.079 (0.991–1.174)	0.934 (0.839–1.041)	1.069 (0.982–1.165)	1.050 (0.962–1.146)	0.941 (0.856–1.035)	1.001 (0.915–1.096)	1.001 (0.915–1.096)	1.032 (0.945–1.126)	0.979 (0.873–1.098)	0.938 (0.840–1.048)
Thyroid cancer											
UKB	0.778 (0.604–1.001)	1.022 (0.833–1.253)	1.176 (1.008–1.373)	1.012 (0.821–1.247)	1.069 (0.877–1.302)	1.277 (1.038–1.572)	1.202 (1.009–1.431)	1.202 (1.009–1.431)	1.172 (0.980–1.400)	1.140 (1.006–1.292)	1.198 (1.050–1.367)
KCPS-II	0.950 (0.873–1.034)	1.000 (0.931–1.074)	1.050 (0.977–1.129)	1.002 (0.931–1.079)	1.015 (0.943–1.092)	1.078 (0.996–1.166)	1.026 (0.956–1.101)	1.026 (0.956–1.101)	1.018 (0.950–1.091)	1.032 (0.956–1.113)	1.048 (0.977–1.123)
Pancreas Cancer											
UKB	1.014 (0.923–1.115)	1.039 (0.955–1.130)	0.999 (0.919–1.086)	1.040 (0.955–1.132)	1.038 (0.955–1.128)	1.001 (0.918–1.093)	1.025 (0.939–1.118)	1.025 (0.939–1.118)	1.028 (0.943–1.120)	1.022 (0.939–1.112)	0.993 (0.912–1.081)
KCPS-II	1.086 (0.920–1.282)	1.073 (0.908–1.269)	0.933 (0.775–1.123)	1.072 (0.900–1.277)	1.044 (0.873–1.250)	0.941 (0.769–1.150)	0.949 (0.786–1.145)	0.949 (0.786–1.145)	0.976 (0.824–1.156)	0.934 (0.769–1.134)	0.887 (0.723–1.089)

Note: *: Overall cancer excluded participants of breast cancer and thyroid cancer. HDL: High-Density Lipoprotein Cholesterol; LDL: Low-density lipoprotein Cholesterol; TG: Triglycerides; TC: Total Cholesterol; Non HDL-C: Non-high-density lipoprotein Cholesterol; AIP: Atherogenic Index of plasma; AC: Atherogenic coefficient; CRI1: Castelli risk index -I; CRI2: Castelli risk index-II; LCI: Lipoprotein combination index; THDL: Triglyceride HDL-C ratio.

In KCPS-II Site-specific associations in KCPS-II were largely null, with two notable exceptions in women. First, cervical cancer risk rose with increasing atherogenic lipids and ratios: per 1-SD increases in LDL-C 1.524 (1.103–2.105; *p* = 0.011), TG 1.101 (1.003–1.209; *p* = 0.042), TC 1.190 (1.062–1.334; *p* = 0.003), non-HDL-C 1.197 (1.067–1.344; *p* = 0.002). Second, thyroid cancer risk was inversely associated with HDL-C (0.900 [0.833–0.972]; *p* = 0.008), while other lipids were not consistently related.

**Table 5:** Hazard ratios (95% CI) for cancer incidence in UKB participants compared to KCPS-II participants.

Cancer Type	KCPS-II	UK Biobank					
		Model 1		Model 2		Model 3	
		HR (95% CI)	E-Value (CI)	HR (95% CI)	E-Value (CI)	HR (95% CI)	E-Value (CI)
Overall cancer	1	1.185 (1.155–1.215)	1.499 (1.446)	1.256 (1.224–1.29)	1.619 (1.566)	1.302 (1.265–1.340)	1.691 (1.633)
Overall cancer *	1	1.450 (1.407–1.494)	1.909 (1.847)	1.536 (1.489–1.585)	2.028 (1.964)	1.608 (1.555–1.663)	2.123 (2.053)
Lung cancer	1	0.689 (0.627–0.758)	1.910 (1.718)	0.671 (0.607–0.742)	1.965 (1.762)	0.713 (0.632–0.805)	1.840 (1.596)
Colon cancer	1	1.562 (1.393–1.752)	2.063 (1.827)	1.663 (1.481–1.869)	2.196 (1.952)	1.718 (1.516–1.947)	2.265 (2.001)
Rectal cancer	1	0.946 (0.834–1.074)	NA	1.028 (0.901–1.173)	NA	1.016 (0.875–1.179)	NA
Stomach cancer	1	0.127 (0.115–0.141)	7.281 (6.796)	0.125 (0.112–0.141)	7.354 (6.823)	0.120 (0.102–0.141)	7.572 (6.820)
Liver cancer	1	0.259 (0.224–0.300)	4.440 (3.977)	0.304 (0.260–0.355)	3.934 (3.479)	0.284 (0.232–0.348)	4.144 (3.541)
Bladder cancer	1	2.675 (2.210–3.237)	3.338 (2.845)	2.840 (2.335–3.454)	3.504 (2.983)	3.308 (2.691–4.066)	3.950 (3.355)
Prostate cancer	1	2.402 (2.192–2.632)	3.054 (2.825)	2.533 (2.307–2.780)	3.192 (2.952)	2.482 (2.247–2.741)	3.138 (2.886)
Breast cancer	1	2.190 (2.033–2.36)	2.823 (2.645)	2.299 (2.131–2.479)	2.943 (2.757)	2.349 (2.166–2.548)	2.997 (2.796)
Cervix uteri cancer	1	1.436 (0.966–2.136)	NA	1.44 (0.960–2.161)	NA	1.039 (0.659–1.637)	NA
Thyroid cancer	1	0.072 (0.064–0.082)	10.330 (9.599)	0.073 (0.064–0.083)	10.315 (9.547)	0.062 (0.051–0.076)	11.285 (10.040)
Pancreas cancer	1	0.897 (0.748–1.074)	NA	1.001 (0.831–1.205)	NA	1.129 (0.916–1.390)	NA
Ovarian cancer	1	2.292 (1.772–2.964)	2.935 (2.332)	2.405 (1.854–3.121)	3.058 (2.433)	2.276 (1.723–3.007)	2.918 (2.271)

Note: *: Overall cancer excluded breast and/or thyroid cancer; Model 1: Cox Proportional-Hazards Model adjusted for age and sex.; Model 2: Cox Proportional-Hazards Model adjusted for age, sex, BMI, dyslipidemia, hypertension, type 2 diabetes mellitus, and cardiovascular disease (CVD); Model 3: Cox Proportional-Hazards Model adjusted for age, sex, BMI, dyslipidemia, hypertension, type 2 diabetes mellitus, CVD, smoking status, alcohol consumption, and physical activity frequency; HR: Hazard ratio; CI: confidence interval; NA: Not available.

### 3.4. Cross-Cohort, Sex-Specific Patterns

In Table 5, fully adjusted Cox models comparing UKB to KCPS-II showed a higher overall cancer risk in UKB (HR 1.30, 95% CI 1.27–1.34), which strengthened when breast and/or thyroid cancers were excluded (HR 1.61, 95% CI 1.56–1.66). Site-specific estimates revealed a consistent elevation for colon (HR 1.72, 1.52–1.95), bladder (HR 3.31, 2.69–4.07), prostate (HR 2.48, 2.25–2.74), breast (HR 2.35, 2.17–2.55), and ovarian cancer (HR 2.28, 1.72–3.01) in UKB relative to KCPS-II, alongside a marked deficit for stomach (HR 0.12, 0.10–0.14), liver (HR 0.28, 0.23–0.35), thyroid (HR 0.06, 0.05–0.08), and lung cancer (HR 0.71, 0.63–0.81). Rectal and pancreatic cancers showed no clear between-cohort difference (HR 1.02, 0.88–1.18; HR 1.13, 0.92–1.39, respectively), and cervical cancer estimates were imprecise (HR 1.04, 0.66–1.64).

## 4. Discussion

This study investigated whether serum lipids and lipid-derived ratios, biomarkers that reflect and modulate oxidative processes, predict cancer risk in the general population. Our analysis of the UKB and KCPS-II cohorts reveals a complex, population- and sex-specific interplay between serum lipid indicators (SLIs) and cancer risk [13,14]. In UKB women, elevated LDL-C was associated with a higher overall cancer risk (HR for 1-SD increase: 1.021; 95% CI: 1.009 to 1.034), an association not observed in KCPS-II women. Conversely, higher HDL-C was protective against overall cancer in women across cohorts (HR: 0.982; 95% CI: 0.969 to 0.995), a benefit not seen in men. Significant sex disparities were evident. While increased LDL-C and total cholesterol were linked to a lower liver cancer risk in men, no such association existed for women. Paradoxically, higher HDL-C levels correlated with an increased risk of cervical cancer in women (HR: 1.167; 95% CI: 1.019 to 1.337) and prostate cancer in men (HR: 1.025; 95% CI: 1.001 to1.049).

Emerging evidence underscores the importance of lipid profiles in influencing cancer susceptibility across various types of cancer, though the exact role differs by lipid type and cancer site. For instance, the Copenhagen General Population Study demonstrated that individuals with HDL-C levels below 39 mg/dL exhibited a significantly higher risk of developing any form of cancer, with hazard ratios (HR) ranging from 1.13 to 1.29, particularly notable in hematological and nervous system cancers [9]. However, the effects of HDL-C on cancer risk are not uniformly protective across all cancer types. Some studies indicate that HDL-C has no substantial impact on the risk of colorectal cancer [7]. Conversely, higher HDL-C levels have been associated with an increased risk of liver cancer [8]. This disparity suggests that the relationship between HDL-C and cancer risk is context-specific, influenced by factors such as the cancer type and the underlying metabolic pathways involved in lipid metabolism.

Serum lipids are embedded in redox-sensitive biochemical networks. Lipid peroxidation of ω-6 polyunsaturated fatty acids yields electrophilic aldehydes—most prominently malondialdehyde (MDA) and 4-hydroxynonenal (4-HNE)—that act as “second messengers” of oxidative stress. These aldehydes form covalent adducts with nucleic acids and proteins, perturbing genome maintenance and signaling. MDA reacts with deoxyguanosine and deoxyadenosine to generate the mutagenic pyrimidopurinone adduct M1dG, which miscodes and is repaired via nucleotide-excision pathways, providing a direct route from lipid peroxidation to fixed mutations [1]. 4-HNE similarly modifies DNA and proteins, modulating proliferation, differentiation, and apoptosis; depending on concentration and cellular context, it can either promote tumor progression or induce cytotoxicity, thereby shaping clonal selection under chronic oxidative stress [2,15].

**Figure 1:**
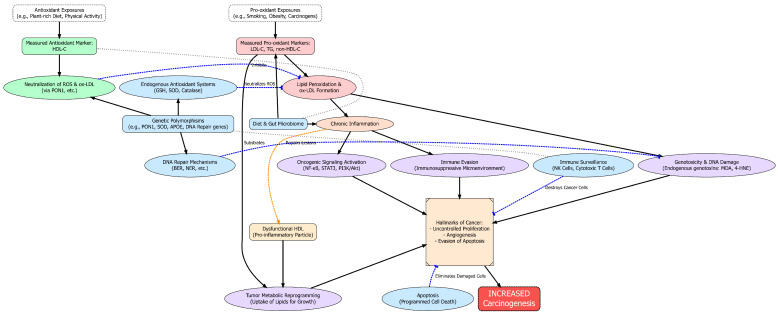
A Conceptual Model of the Interplay between Pro-oxidant and Protective Mechanisms in Lipid-Mediated Carcinogenesis. Solid arrows indicate stimulatory or causal pathways, while blunt-ended lines represent inhibitory or protective processes.

In inflamed tissues, oxidized low-density lipoprotein (ox-LDL) accumulates and ligates the scavenger receptor LOX-1, amplifying reactive oxygen species, activating NF-κB/STAT3 signaling, and fostering an epithelial–mesenchymal transition phenotype—molecular programs linked to tumor growth, invasion, and therapy resistance [3,16]. Although high-density lipoprotein (HDL) is classically anti-oxidative—via enzymes such as paraoxonase-1 (PON1) and lipid transfer proteins—systemic inflammation can render HDL “dysfunctional.” Myeloperoxidase (MPO)–mediated oxidation of apoA-I impairs cholesterol efflux and inactivates PON1, converting HDL from an anti- to pro-inflammatory particle with diminished redox-buffering capacity [4,5,6]. Concurrently, many tumors up-regulate the HDL receptor SR-B1, enhancing HDL-cholesterol uptake to support membrane synthesis and steroidogenesis; experimental SR-B1 loss reduces HDL-driven proliferation and disease progression, underscoring a pathway through which “protective” lipoproteins can, in specific microenvironments, fuel oncogenesis [17,18]. These lipid–redox interactions intersect canonical stress pathways (e.g., KEAP1–NRF2 and NF-κB), linking chronic inflammation to mutagenesis, survival signaling, and metabolic rewiring—mechanistic scaffolding that explains why population associations between serum lipids and cancer can be directionally heterogeneous and context dependent (Figure 1) [19]. The carcinogenic cascade begins with Pro-oxidant Exposures (e.g., obesity, smoking), which elevate circulating pro-oxidant lipid markers (LDL-C, TG). These lipids are substrates for Lipid Peroxidation, a process that generates two key oncogenic drivers. First, it produces endogenous genotoxins (e.g., MDA, 4-HNE) that cause direct DNA damage, leading to mutations. Second, it forms oxidized LDL (ox-LDL), which fuels chronic inflammation. This inflammatory state activates oncogenic signaling (e.g., NF-κB, STAT3), promotes immune evasion, and provides substrates for tumor metabolic reprogramming, where cancer cells exploit lipids for growth. Collectively, these pathways drive the Hallmarks of Cancer, increasing the overall risk of carcinogenesis. This pro-carcinogenic cascade is continuously opposed by a sophisticated network of protective mechanisms, supported by Antioxidant Exposures (e.g., plant-rich diet) and reflected in higher HDL-C levels. The first line of defense includes Endogenous Antioxidant Systems (e.g., SOD, GSH) and the enzymatic functions of HDL (via PON1), which neutralize ROS and prevent lipid peroxidation. If damage occurs, robust DNA Repair Mechanisms are activated to preserve genomic integrity. Cells with irreparable damage are eliminated by Apoptosis (programmed cell death), a critical tumor-suppressive checkpoint. Finally, Immune Surveillance by NK and T cells recognizes and destroys nascent cancer cells.

Observed disparities in cancer incidence, where females exhibit higher rates for certain malignancies such as thyroid cancer, may be partially attributable to sex-based differences in healthcare-seeking behaviors, leading to greater diagnostic surveillance in women [20]. One crucial aspect of these sex-specific differences lies in the role of sex hormones. Estrogen and testosterone play a pivotal role in regulating lipid metabolism and, consequently, cancer development. For instance, estrogen, while generally considered protective against cardiovascular disease, has been linked to an increased risk of certain cancers, such as breast cancer. Studies have shown that estrogen may promote cell proliferation in breast tissue, leading to an increased risk of breast cancer [21]. Conversely, free testosterone, rather than total testosterone, is associated with an increased risk of prostate cancer, potentially due to its role in promoting cell proliferation and growth in the prostate gland [22]. Further exploration of the specific mechanisms by which sex hormones influence SLI levels and cancer risk, including their interactions with other metabolic pathways, is crucial for developing tailored preventive and therapeutic strategies.

Behavioral factors further contribute to the observed sex-specific differences. A comprehensive understanding of these factors is essential for understanding the complexities of SLI-cancer risk associations. Smoking prevalence is generally higher among men, and this increased exposure to carcinogens can significantly impact both SLI levels and cancer risk [23]. Similarly, dietary habits, particularly alcohol consumption, exhibit sex-specific patterns that could influence SLI levels and subsequent cancer risk [24]. Another study suggests that obesity-associated inflammation may induce an androgenic to estrogenic switch in the prostate gland, potentially influencing prostate cancer risk [25]. These differences in behaviors, when combined with the underlying biological influences of sex hormones, contribute to the intricate interplay between SLI and cancer risk in a sex-specific manner.

The potential differences in ethnicity could also alter the relationship between serum lipid indices (SLIs) and ratios with cancer [26]. The hazard ratio (HR) for overall cancer risk by ethnicity was 1.302 (95% CI: 1.265–1.340), and the e-value was 1.691 with a confidence interval (1.633). Sensitivity analysis showed that some cancer risk factors, including factors such as environmental pollution, infections, and dietary habits (e.g., red meat consumption), are potential confounders that could influence the observed differences [27].

The E-values presented in Table 5 provide an important quantitative assessment of the robustness of the observed inter-cohort differences against unmeasured confounding. For highly significant differences, such as the deficit of stomach cancer (HR 0.12, E-value > 7.5) and thyroid cancer (HR 0.06, E-value > 11.2) in UKB relative to KCPS-II, the very high E-values indicate that an unmeasured confounder would need to be extraordinarily strong to explain these associations. For instance, for thyroid cancer, an unmeasured confounder would need to be associated with both cohort and outcome by more than 11-fold to negate the observed HR. Conversely, for associations with E-values closer to the observed HR, such as bladder cancer (HR 3.31, E-value 3.95), the results are more susceptible to unmeasured confounding. These E-values underscore that while our findings are robust for some associations, others require further validation and consideration of context-specific confounders.

### 4.1. Strengths

This study benefits from the use of two large, well-characterized cohorts, the UKB and the KCPS-II Biobank, which enhances the study’s statistical power and generalizability. Moreover, the prospective design, with data collected prior to cancer diagnosis, allows for a temporal assessment of SLIs and cancer risk, minimizing the risk of reverse causality. The study further strengthens its validity by adjusting for a wide range of potential confounders, including age, sex, body mass index, diabetes status, hypertension, cardiovascular disease, smoking status, physical activity frequency and alcohol consumption.

### 4.2. Limitations

Firstly, the observational nature of the study design precludes definitive causal inferences. While we have adjusted for a range of potential confounders, the possibility remains that unmeasured confounders might influence the observed associations. Secondly, although the study included two diverse populations, the findings might not be generalizable to all populations and ethnicities, due to potential differences in genetic background, lifestyle, and environmental exposures. Finally, the measurement of SLIs at a single time point is a limitation. Lipid levels can fluctuate over time, and a single measurement might not accurately reflect long-term exposure.

### 4.3. Practical Implications

These findings could inform clinical practice by prompting physicians to consider a more personalized approach to risk assessment and potentially more frequent screening for individuals with elevated SLI levels in these specific groups. Furthermore, this study underscores the need for tailored prevention strategies that consider an individual’s SLI profile, sex, ethnicity, and other risk factors. A one-size-fits-all approach to cancer prevention is unlikely to be effective, as the observed sex-specific differences highlight the importance of considering individual susceptibility.

### 4.4. Future Research

Future research should integrate genetic, metabolic, and environmental data to elucidate the causal architecture of lipid-mediated carcinogenesis and to identify intervention windows for effective, precision-based cancer prevention.

## 5. Conclusions

Our findings reveal that the association between serum lipid profiles, as systemic indicators of redox status, and cancer risk is profoundly heterogeneous across distinct populations and sexes. In the UK Biobank, markers of pro-oxidant lipids were associated with increased overall cancer risk, while HDL-C showed a complex, context-dependent pattern, consistent with lipid-mediated oxidative stress playing a role in carcinogenesis. However, these associations were largely absent in the Korean Cancer Prevention Study-II, suggesting population-specific thresholds or modulating factors for redox-mediated cancer development. This work challenges the notion of a universal role for lipid-mediated oxidative stress in carcinogenesis and emphasizes the critical need for population-specific research to inform our understanding of the complex interplay between metabolic factors, redox biology, and cancer etiology.

## Figures and Tables

**Table 2:** Incidence Rate per 100,000 Person-Years by sex, cohort, and cancer type.

Cancer Type	UK Biobank		KCPS-II Biobank	
	Women	Men	Women	Men
OVERALL				
N	196,739	171,549	51,949	86,144
Overall cancer	1238.28	1472.89	572.09	456.38
Overall cancer *	930.02	1467.45	274.53	380.04
Lung cancer	66.44	83.28	23.56	43.26
Colon cancer	80.27	111.06	17.49	23.92
Rectal cancer	27.36	53.12	13.36	22.25
Stomach cancer	10.33	26.25	31.98	73.70
Liver cancer	9.64	19.63	6.47	26.17
Bladder cancer	25.39	93.15	2.34	10.16
Prostate cancer	0	424.80	0	53.32
Breast cancer	437.16	4.67	130.08	0.42
Cervix uteri cancer	10.54	0	7.99	0
Thyroid cancer	12.82	5.76	168.47	77.01
Pancreas cancer	20.09	29.17	6.74	11.32
Ovarian cancer	43.72	0	10.74	0
EARLY ONSET CANCER				
N	47,971	41,246	42,971	71,191
Overall cancer	389.17	201.06	458.45	212.90
Overall cancer *	260.54	199.99	171.25	128.05
Lung cancer	7.46	4.77	6.52	6.81
Colon cancer	16.78	11.66	7.61	7.64
Rectal cancer	6.52	5.30	8.26	15.15
Stomach cancer	0.47	3.18	16.75	25.30
Liver cancer	0	1.06	0.87	9.03
Bladder cancer	2.80	5.83	1.09	2.22
Prostate cancer	0	12.19	0	2.92
Breast cancer	191.38	0	114.98	0.14
Cervix uteri cancer	9.32	0	8.48	0
Thyroid cancer	9.79	2.65	173.63	84.42
Pancreas cancer	2.80	4.24	1.09	2.08
Ovarian cancer	20.98	0	7.39	0

Note: *: Overall cancer excluded breast and/or thyroid cancer; UK: United Kingdom; KCPS-II: Korean Cancer Prevention Study-II.

## Data Availability

The data presented in this study are available on request from the corresponding author. The data are not publicly available due to privacy and ethical restrictions.

## Supplementary Materials

The following supporting information can be downloaded at: https://www.mdpi.com/article/10.3390/antiox14091135/s1, Figure S1. Flowchart of the Data Cleaning and Preparation Process for the Study, Table S1. ICD-10 codes used for disease classification in research; Table S2. Formulas for calculating serum lipid ratios; Table S3. Incidence case by sex, cohorts, and cancer type; Table S4. Results of the estimated association between serum lipids and overall cancer risk for UK Biobank data; Table S5. Results of the estimated association between serum lipids and overall cancer risk in KCPS-II Biobank; Table S6. R Packages.

## Abbreviations

The following abbreviations are used in this manuscript:

4-HNE	4-hydroxynonenal
AC	Atherogenic coefficient
AIP	Atherogenic Index of Plasma
BMI	Body Mass Index
CI	Confidence Interval
CRI-I	Castelli Risk Index-I
CRI-II	Castelli Risk Index-II
CVD	Cardiovascular Disease
FBS	Fasting Blood Sugar
HDL-C	High-density Lipoprotein Cholesterol
HR	Hazard Ratio
ICD-10	International Classification of Diseases, 10th Revision
KCPS-II	Korean Cancer Prevention Study-II
LCI	Lipoprotein Combination Index
LDL-C	Low-density Lipoprotein Cholesterol
MDA	Malondialdehyde
ox-LDL	Oxidized Low-density Lipoprotein
SD	Standard Deviation
SLIs	Serum Lipid Indicators
T2DM	Type 2 Diabetes Mellitus
TC	Total Cholesterol
TG	Triglycerides
THDL	Triglyceride HDL-C Ratio
UKB	UK Biobank
