# Supplementary material for: Sex- and Ethnic-Specific Associations of Serum Lipids with Risk of 12 Cancers: Findings from 506,381 Adults in Two Large Cohorts
Source: Antioxidants (Basel). 2025 Sep 19;14(9):1135. doi: 10.3390/antiox14091135 (PMC12466349; doi:10.3390/antiox14091135)
Supplement: Supplementary file 1 [file antioxidants-14-01135-s001.zip › antioxidants-3840195-supplementary.pdf]

Title: Sex- and Ethnic-Specific Associations of Serum Lipids with Risk of 12 Cancers: Findings from 506,381 Adults in Two Large Cohorts

Supplementary Tables, Figures and Methods

Supplementary Methods.

Reasons for excluding research subjects

For the UK Biobank cohort, the initial cohort included 502,478 adults. We excluded 114,219 participants based on the following criteria: missing data on body mass index (BMI) (n=1,614); serum lipids (LDL-C, HDL-C, triglycerides, and total cholesterol) (n=73,617); fasting blood glucose (n=25,071); a diagnosis of cancer prior to enrollment or within two years of baseline recruitment (n= 31,945); and insufficient data on health-related behaviors, including smoking history, alcohol consumption, and physical activity (n=1,943). For the KCPS-II cohort, the initial cohort included 156,701 adults. We excluded 18,608 participants based on the following criteria: missing data on serum lipids (LDL-C, HDL-C, triglycerides, and total cholesterol) (n=639); fasting blood glucose (n=40); a diagnosis of cancer prior to enrollment or within two years of baseline recruitment (n=4,177); and insufficient data on health-related behaviors, including smoking history, alcohol consumption, and physical activity (n=13,752).

Covariables

Potential confounders included age (continuous), gender (male, female), body mass index (continuous), T2DM (yes, no), hypertension (yes, no), CVD (yes, no), smoking status (never, former, current), physical activity (number of days per week engaging in moderate physical activity for at least 10 minutes), alcohol consumption (frequency, number of days per week consuming more than one standard drink), and continuous measurements of gamma-glutamyl transferase (GGT), alanine aminotransferase (ALT), and aspartate aminotransferase (AST). Subgroup analyses by BMI utilised the following categorical cutoff points: for the UK population, underweight (<18.5) and normal weight (18.5 to 24.9), overweight (25.0 to 29.9), and obesity (≥30.0); for the Korean population, underweight (< 18.5), normal weight (18.5 to 22.9), overweight (23 to 26.9), and obesity (≥ 27). Dyslipidaemia was defined as TC ≥ 200 mg/dL, TG ≥ 150 mg/dL, LDL-C ≥ 130 mg/dL, or HDL-C < 40 mg/dL for men and HDL-C < 50 mg/dL for women. To convert lipid values from mg/dL to mmol/L, the following conversion factors were applied: for total cholesterol (TC), LDL-C, and HDL-C, divide by 38.67; for triglycerides (TG), divide by 88.57. The corresponding thresholds for dyslipidaemia after conversion are:

TC ≥ 5.18 mmol/L, TG ≥ 1.7 mmol/L, LDL-C ≥ 3.37 mmol/L, HDL-C < 1.03 mmol/L for men, and HDL-C < 1.29 mmol/L for women.

Statistical Analysis

Serum lipid profiles and lipid ratios were categorized into five quintiles for analysis within each subgroup defined by gender, Body mass index (BMI), cardiovascular disease (CVD), Type 2 Diabetes Mellitus (T2DM), and hypertension. The formula for standardising serum lipid indexes or ratios involves subtracting the mean and dividing by the standard deviation.

$$Z_{lipid} = \frac{X_{lipid} - \mu_{lipid}}{\sigma_{lipid}}$$

Where:

- Z_{lipid} is the standardised value (z-score),
- X_{lipid} is the original serum lipid index or ratio value,
- μ_{lipid} is the mean of the serum lipid index or ratio,
- σ_{lipid} is the standard deviation of the serum lipid index or ratio.

Cox proportional hazard models were employed to estimate hazard ratios (HR) and 95% confidence intervals (CIs). A two-slide p-value of less than 0.05 was considered statistically significant.

The incidence rate per 100,000 person-years was calculated for each gender and cohort. Trend analyses were conducted within each quintile to assess differences in cancer incidence and associated risks. Quintile categorisation for each serum lipid indices and the ratio was recalculated for each gender, cohort, and cancer type. Additionally, the impact of a one-standard-deviation increase in serum lipid indices and ratios on cancer risk was reported. Serum lipid indices and ratios were standardised by the mean and standard deviation. When analysing cancer risk by gender, prostate cancer analyses were limited to men, while breast cancer and cervical cancer analyses were restricted to female participants.

All statistical analyses and data presentations were performed using R version 4.3.3.

Figure S1. Flowchart of the Data Cleaning and Preparation Process for the Study

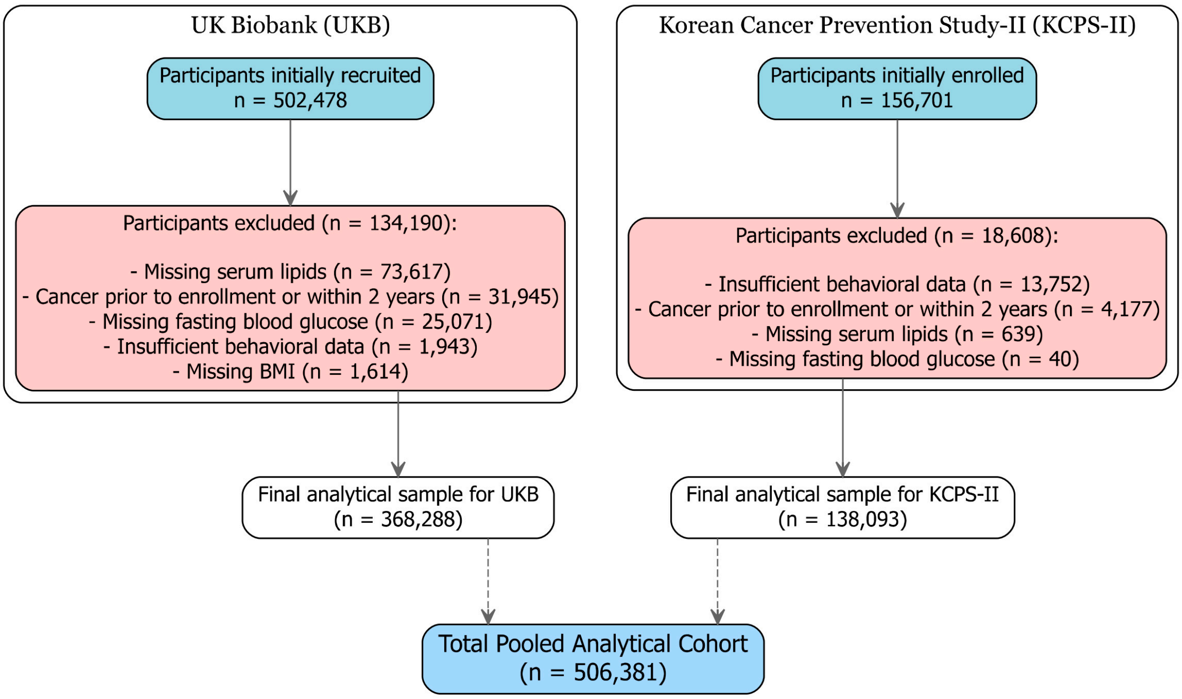

Table S1. ICD-10 codes used for disease classification in research.

Disease	ICD-10 code
Type 2 diabetes	E11 Non-insulin-dependent diabetes mellitus
Hypertension	I10: Essential (primary) hypertension<br>I11: Hypertensive heart disease<br>I12: Hypertensive chronic kidney disease<br>I13: Hypertensive heart and chronic kidney disease
Cardiovascular disease	I20 - I25: Ischaemic Heart Diseases<br>I60 - I64: Cerebrovascular Diseases
Lung cancer	C34 Malignant neoplasm of bronchus and lung
Conlon cancer	C18 Malignant neoplasm of colon<br>C19 Malignant neoplasm of rectosigmoid junction
Rectal cancer	C20 Malignant neoplasm of rectum
Stomach cancer	C16 Malignant neoplasm of stomach
Liver cancer	C22 Malignant neoplasm of liver and intrahepatic bile ducts
Bladder cancer	C67 - Malignant neoplasm of bladder
Prostate cancer	C61 - Malignant neoplasm of prostate
Breast cancer	C50 - Malignant neoplasm of breast
Cervix uteri cancer	C53 - Malignant neoplasm of cervix uteri
Pancreas cancer	C25 - Malignant neoplasm of pancreas
Leukemia cancer	C91 - Lymphoid leukaemia<br>C92 - Myeloid leukaemia<br>C93 - Monocytic leukaemia<br>C94 - Other leukaemias of specified cell type<br>C95 - Leukaemia of unspecified cell type
Nervous cancer: Malignant neoplasms of the brain and other parts of the central nervous system.	C70 - Malignant neoplasm of meninges<br>C71 - Malignant neoplasm of brain<br>C72 - Malignant neoplasm of spinal cord, cranial nerves, and other parts of central nervous system
Thyorid cancer	C73: Malignant neoplasm of thyroid gland
Ovarian cacner	C56: Malignant neoplasm of ovary

Table S2. Formulas for calculating serum lipid ratios.

Serum lipids ratios	Formula
Atherogenic Index of Plasma (AIP)	$$\text{AIP} = \log\left(\frac{\text{Triglycerides (TG)}}{\text{HDL-C}}\right)$$ Triglycerides (TG) and HDL-C should be in mmol/L for this calculation. If they are in mg/dL, convert them to mmol/L by dividing by 88.5 for triglycerides and 38.7 for HDL-C.
Atherogenic Coefficient (AC)	$$\text{AC} = \frac{\text{Total Cholesterol (TC)} - \text{HDL-C}}{\text{HDL-C}}$$ Total Cholesterol (TC) and HDL-C should be in the same units (mg/dL or mmol/L).
Castelli's Index-I (CRI-I)	$$\text{CRI-I} = \frac{\text{Total Cholesterol (TC)}}{\text{HDL-C}}$$ Total Cholesterol (TC) and HDL-C should be in the same units (mg/dL or mmol/L).
Castelli's Index-II (CRI-II)	$$\text{CRI-II} = \frac{\text{LDL-C}}{\text{HDL-C}}$$ LDL-C and HDL-C should be in the same units (mg/dL or mmol/L).
Non-HDL-C	$$\text{Non-HDL-C} = \text{Total Cholesterol} - \text{HDL-C}$$ Total Cholesterol (TC) and HDL-C in the same units.
Lipoprotein Combination Index (LCI)	$$\text{LCI} = \frac{\text{Total Cholesterol} \times \text{Triglycerides} \times \text{LDL-C}}{\text{HDL-C}}$$ Total Cholesterol (TC), Triglycerides (TG), LDL-C and HDL-C in the same units.
Triglyceride HDL-C ratio	$$\text{Triglycerides/HDL-C Ratio} = \frac{\text{Triglycerides (TG)}}{\text{HDL-C}}$$ Triglycerides (TG) and HDL-C are measured in the same units, either mg/dL or mmol/L.

Table S3. Incidence case by sex, cohorts, and cancer type

Cancer type	UK Biobank		KCPS-II Biobank	
	Women	Men	Women	Men
**OVERALL**	n=196,739	n= 171,549	n= 51,949	n= 86,144
Overall cancer, n(%)				
No	169,282 (86.0%)	143,497 (83.6%)	47,925 (92.3%)	80,788 (93.8%)
Yes	27,457 (14.0%)	28,052 (16.4%)	4,024 (7.75%)	5,356 (6.22%)
Overall cancer *, n(%)				
No	175,797 (89.4%)	143,594 (83.7%)	50,018 (96.3%)	81,684 (94.8%)
Yes	20,942 (10.6%)	27,955 (16.3%)	1,931 (3.72%)	4,460 (5.18%)
Lung cancer, n(%)				
No	195,190 (99.2%)	169,875 (99.0%)	51,778 (99.7%)	85,625 (99.4%)
Yes	1,549 (0.79%)	1,674 (0.98%)	171 (0.33%)	519 (0.60%)
Colon cancer				
No	19,4871 (99.1%)	169,323 (98.7%)	51,822 (99.8%)	85,857 (99.7%)
Yes	1,868 (0.95%)	2,226 (1.30%)	127 (0.24%)	287 (0.33%)
Rectal cancer, n(%)				
No	196,101 (99.7%)	170,482 (99.4%)	51,852 (99.8%)	85,877 (99.7%)
Yes	638 (0.32%)	1,067 (0.62%)	97 (0.19%)	267 (0.31%)
Stomach cancer, n(%)				
No	196,498 (99.9%)	171,021 (99.7%)	51,717 (99.6%)	85,262 (99.0%)
Yes	241 (0.12%)	528 (0.31%)	232 (0.45%)	882 (1.02%)
Liver cancer, n(%)				
No	196,514 (99.9%)	171,154 (99.8%)	51,902 (99.9%)	85,830 (99.6%)
Yes	225 (0.11%)	395 (0.23%)	47 (0.09%)	314 (0.36%)
Bladder cancer, n(%)				
No	196,147 (99.7%)	169,681 (98.9%)	51,932 (100.0%)	86,022 (99.9%)
Yes	592 (0.30%)	1,868 (1.09%)	17 (0.03%)	122 (0.14%)
Prostate cancer, n(%)				
No	196,739 (100.0%)	163,206 (95.1%)	51,949 (100%)	85,505 (99.3%)
Yes	0 (0.00%)	8,343 (4.86%)	0 (0.00%)	639 (0.74%)
Breast cancer, n(%)				
No	186,748 (94.9%)	171,455 (99.9%)	51,010 (98.2%)	86,139 (100.0%)
Yes	9,991 (5.08%)	94 (0.05%)	939 (1.81%)	5 (0.01%)
Cervix uteri cancer, n(%)				
No	196,493 (99.9%)	171,549 (100%)	51,891 (99.9%)	86,144 (100%)
Yes	246 (0.13%)	0 (0.00%)	58 (0.11%)	0 (0.00%)
Thyroid cancer, n(%)				
No	196,440 (99.8%)	171,433 (99.9%)	50,740 (97.7%)	85,224 (98.9%)
Yes	299 (0.15%)	116 (0.07%)	1,209 (2.33%)	920 (1.07%)
Pancreas cancer, n(%)				
No	196,270 (99.8%)	170,962 (99.7%)	51,900 (99.9%)	86,008 (99.8%)
Yes	469 (0.24%)	587 (0.34%)	49 (0.09%)	136 (0.16%)
Ovarian cancer, n(%)				
No	195,720 (99.5%)	171,549 (100.0%)	51,871 (99.8%)	86,144 (100%)
Yes	1,019 (0.52%)	0 (0.00%)	78 (0.15%)	0 (0.00%)

Note: *Overall cancer excluded breast and/ or thyroid cancer; UK: United Kingdom; KCPS-II: Korean Cancer Prevention Study-II.

Table S3. Incidence case by sex, cohorts, and cancer type (cont.)

Cancer type	UK Biobank		KCPS-II Biobank	
	Women	Men	Women	Men
**EARLY ONSET CANCER**	n= 47971	n= 41246	n=42971	n=71191
Overall cancer, n(%)				
No	47,143 (98.3%)	40,868 (99.1%)	40,907 (95.2%)	69,673 (97.9%)
Yes	828 (1.73%)	378 (0.92%)	2064 (4.80%)	1518 (2.13%)
Overall cancer * , n(%)				
No	47,415 (98.8%)	40,870 (99.1%)	42,200 (98.2%)	70,278 (98.7%)
Yes	556 (1.16%)	376 (0.91%)	771 (1.79%)	913 (1.28%)
Lung cancer, n(%)				
No	47,955 (100.0%)	41,237 (100.0%)	42,941 (99.9%)	71,142 (99.9%)
Yes	16 (0.03%)	9 (0.02%)	30 (0.07%)	49 (0.07%)
Colon cancer, n(%)				
No	47,935 (99.9%)	41,224 (99.9%)	42,936 (99.9%)	71,136 (99.9%)
Yes	36 (0.08%)	22 (0.05%)	35 (0.08%)	55 (0.08%)
Rectal cancer, n(%)				
No	47,957 (100.0%)	41,236 (100.0%)	42,933 (99.9%)	71,082 (99.8%)
Yes	14 (0.03%)	10 (0.02%)	38 (0.09%)	109 (0.15%)
Stomach cancer, n(%)				
No	47,970 (100.0%)	41,240 (100.0%)	42,894 (99.8%)	71,009 (99.7%)
Yes	1 (0.00%)	6 (0.01%)	77 (0.18%)	182 (0.26%)
Liver cancer, n(%)				
No	47,971 (100%)	41,244 (100.0%)	42,967 (100.0%)	71,126 (99.9%)
Yes	0 (0.00%)	2 (0.00%)	4 (0.01%)	65 (0.09%)
Bladder cancer, n(%)				
No	47,965 (100.0%)	41,235 (100.0%)	42,966 (100.0%)	71,175 (100.0%)
Yes	6 (0.01%)	11 (0.03%)	5 (0.01%)	16 (0.02%)
Prostate cancer, n(%)				
No	47,971 (100%)	41,223 (99.9%)	42,971 (100%)	71,170 (100.0%)
Yes	0 (0.00%)	23 (0.06%)	0 (0.00%)	21 (0.03%)
Breast cancer, n(%)				
No	47,562 (99.1%)	41,246 (100%)	42,444 (98.8%)	71,190 (100.0%)
Yes	409 (0.85%)	0 (0.00%)	527 (1.23%)	1 (0.00%)
Cervix uteri cancer, n(%)				
No	47,951 (100.0%)	41,246 (100%)	42,932 (99.9%)	71,191 (100%)
Yes	20 (0.04%)	0 (0.00%)	39 (0.09%)	0 (0.00%)
Thyroid cancer, n(%)				
No	47,950 (100.0%)	41,241 (100.0%)	42,180 (98.2%)	70,586 (99.2%)
Yes	21 (0.04%)	5 (0.01%)	791 (1.84%)	605 (0.85%)
Pancreas cancer, n(%)				
No	47,965 (100.0%)	41,238 (100.0%)	42,966 (100.0%)	71,176 (100.0%)
Yes	6 (0.01%)	8 (0.02%)	5 (0.01%)	15 (0.02%)
Ovarian cancer, n(%)				
No	47,926 (99.9%)	41,246 (100%)	42,937 (99.9%)	71,191 (100%)
Yes	45 (0.09%)	0 (0.00%)	34 (0.08%)	0 (0.00%)

Note: *Overall cancer excluded breast and/ or thyroid cancer; UK: United Kingdom; KCPS-II: Korean Cancer Prevention Study-II.

Table S4. Results of the estimated association between serum lipids and overall cancer risk for UK Biobank data

Gender	Cancer type	Lipid index	1st quintile	2nd quintile		3rd quintile		4th quintile		5th quintile		p-value for trend	Per 1 SD increase	
				aHR (95% CI)	p-value	aHR (95% CI)	p-value	aHR (95% CI)	p-value	aHR (95% CI)	p-value		aHR (95% CI)	p-value
Women	Overall cancer	HDL-C	1	0.966 (0.930 to 1.002)	0.066	0.968 (0.931 to 1.005)	0.0927	0.951 (0.914 to 0.989)	0.0127	0.938 (0.901 to 0.978)	0.0024	0.0029	0.982 (0.969 to 0.995)	0.0076
Women	Overall cancer	LDL-C	1	1.058 (1.018 to 1.100)	0.004	1.025 (0.986 to 1.066)	0.2073	1.030 (0.991 to 1.070)	0.1353	1.079 (1.039 to 1.121)	<0.001	0.0034	1.021 (1.009 to 1.034)	<0.001
Women	Overall cancer	TG	1	1.026 (0.985 to 1.069)	0.2204	1.062 (1.019 to 1.105)	0.0039	1.064 (1.022 to 1.109)	0.0027	1.077 (1.034 to 1.123)	<0.001	<0.001	1.020 (1.007 to 1.032)	0.0015
Women	Overall cancer	TC	1	1.042 (1.002 to 1.083)	0.041	1.044 (1.004 to 1.085)	0.0311	1.028 (0.989 to 1.069)	0.1634	1.061 (1.021 to 1.102)	0.0027	0.0189	1.016 (1.004 to 1.029)	0.008
Women	Overall cancer	NONHDL	1	1.039 (0.999 to 1.080)	0.0561	1.014 (0.975 to 1.054)	0.4808	1.028 (0.989 to 1.068)	0.1663	1.082 (1.042 to 1.124)	<0.001	<0.001	1.022 (1.010 to 1.035)	<0.001
Women	Overall cancer	AIP	1	1.028 (0.987 to 1.070)	0.179	1.050 (1.009 to 1.093)	0.0167	1.053 (1.011 to 1.096)	0.0125	1.089 (1.044 to 1.135)	<0.001	<0.001	1.029 (1.015 to 1.043)	<0.001
Women	Overall cancer	AC	1	1.024 (0.985 to 1.064)	0.2399	0.996 (0.958 to 1.036)	0.8342	1.041 (1.001 to 1.083)	0.0427	1.083 (1.042 to 1.127)	<0.001	<0.001	1.031 (1.019 to 1.044)	<0.001
Women	Overall cancer	CRI-I	1	1.024 (0.985 to 1.064)	0.2399	0.996 (0.958 to 1.036)	0.8342	1.041 (1.001 to 1.083)	0.0427	1.083 (1.042 to 1.127)	<0.001	<0.001	1.031 (1.019 to 1.044)	<0.001
Women	Overall cancer	CRI-II	1	1.051 (1.011 to 1.092)	0.0119	1.010 (0.971 to 1.050)	0.6311	1.034 (0.995 to 1.076)	0.089	1.108 (1.066 to 1.152)	<0.001	<0.001	1.032 (1.019 to 1.044)	<0.001
Women	Overall cancer	LCI	1	1.053 (1.011 to 1.096)	0.0118	1.045 (1.004 to 1.088)	0.0307	1.068 (1.026 to 1.112)	0.0013	1.101 (1.058 to 1.147)	<0.001	<0.001	1.023 (1.012 to 1.035)	<0.001
Women	Overall cancer	THDL	1	1.028 (0.987 to 1.070)	0.179	1.050 (1.009 to 1.093)	0.0167	1.053 (1.011 to 1.096)	0.0125	1.089 (1.044 to 1.135)	<0.001	<0.001	1.022 (1.009 to 1.034)	<0.001
Women	Overall cancer*	HDL-C	1	0.954 (0.914 to 0.996)	0.0302	0.948 (0.907 to 0.990)	0.0159	0.934 (0.893 to 0.978)	0.0032	0.912 (0.870 to 0.956)	<0.001	<0.001	0.974 (0.959 to 0.990)	<0.001
Women	Overall cancer*	LDL-C	1	1.062 (1.016 to 1.110)	0.0081	1.046 (1.000 to 1.093)	0.0489	1.041 (0.997 to 1.088)	0.071	1.088 (1.041 to 1.136)	<0.001	0.0027	1.024 (1.010 to 1.038)	<0.001
Women	Overall cancer*	TG	1	1.017 (0.970 to 1.066)	0.4877	1.048 (1.000 to 1.098)	0.051	1.054 (1.006 to 1.104)	0.0285	1.083 (1.032 to 1.135)	0.001	<0.001	1.023 (1.009 to 1.037)	0.0011
Women	Overall cancer*	TC	1	1.043 (0.997 to 1.090)	0.0673	1.050 (1.004 to 1.098)	0.0313	1.042 (0.997 to 1.090)	0.0662	1.063 (1.017 to 1.110)	0.007	0.0188	1.018 (1.004 to 1.032)	0.012
Women	Overall cancer*	NONHDL	1	1.048 (1.002 to 1.095)	0.0414	1.025 (0.980 to 1.072)	0.2774	1.045 (1.000 to 1.092)	0.0516	1.092 (1.046 to 1.141)	<0.001	<0.001	1.026 (1.012 to 1.040)	<0.001
Women	Overall cancer*	AIP	1	1.035 (0.988 to 1.084)	0.1501	1.045 (0.997 to 1.094)	0.0638	1.063 (1.015 to 1.113)	0.0101	1.104 (1.053 to 1.158)	<0.001	<0.001	1.035 (1.019 to 1.050)	<0.001
Women	Overall cancer*	AC	1	1.029 (0.984 to 1.076)	0.2122	1.003 (0.959 to 1.049)	0.8857	1.071 (1.024 to 1.120)	0.0027	1.101 (1.053 to 1.152)	<0.001	<0.001	1.038 (1.024 to 1.053)	<0.001
Women	Overall cancer*	CRI-I	1	1.029 (0.984 to 1.076)	0.2122	1.003 (0.959 to 1.049)	0.8857	1.071 (1.024 to 1.120)	0.0027	1.101 (1.053 to 1.152)	<0.001	<0.001	1.038 (1.024 to 1.053)	<0.001
Women	Overall cancer*	CRI-II	1	1.054 (1.008 to 1.101)	0.0215	1.014 (0.969 to 1.060)	0.5572	1.062 (1.016 to 1.111)	0.0084	1.124 (1.075 to 1.176)	<0.001	<0.001	1.038 (1.024 to 1.053)	<0.001
Women	Overall cancer*	LCI	1	1.063 (1.015 to 1.114)	0.0092	1.040 (0.993 to 1.090)	0.0949	1.085 (1.036 to 1.136)	<0.001	1.119 (1.069 to 1.172)	<0.001	<0.001	1.025 (1.012 to 1.038)	<0.001
Women	Overall cancer*	THDL	1	1.035 (0.988 to 1.084)	0.1501	1.045 (0.997 to 1.094)	0.0638	1.063 (1.015 to 1.113)	0.0101	1.104 (1.053 to 1.158)	<0.001	<0.001	1.025 (1.012 to 1.039)	<0.001
Women	Lung cancer	HDL-C	1	0.910 (0.784 to 1.057)	0.2159	0.957 (0.820 to 1.117)	0.5758	0.819 (0.693 to 0.968)	0.0192	0.712 (0.596 to 0.851)	<0.001	<0.001	0.912 (0.861 to 0.966)	0.0018

Gender	Cancer type	Lipid index	1st quintile	2nd quintile		3rd quintile		4th quintile		5th quintile		p-value for trend	Per 1 SD increase	
				aHR (95% CI)	p-value	aHR (95% CI)	p-value	aHR (95% CI)	p-value	aHR (95% CI)	p-value		aHR (95% CI)	p-value
Women	Lung cancer	LDL-C	1	1.021 (0.870 to 1.199)	0.7974	1.053 (0.898 to 1.235)	0.5234	0.922 (0.785 to 1.083)	0.3241	0.944 (0.808 to 1.103)	0.4706	0.2238	0.976 (0.930 to 1.023)	0.3113
Women	Lung cancer	TG	1	1.214 (0.996 to 1.481)	0.0554	1.344 (1.108 to 1.630)	0.0027	1.359 (1.124 to 1.643)	0.0016	1.412 (1.168 to 1.708)	<0.001	<0.001	1.060 (1.015 to 1.107)	0.0079
Women	Lung cancer	TC	1	1.115 (0.948 to 1.311)	0.189	1.102 (0.937 to 1.296)	0.2414	0.990 (0.841 to 1.165)	0.8999	0.945 (0.805 to 1.111)	0.4946	0.171	0.967 (0.922 to 1.015)	0.1728
Women	Lung cancer	NONHDL	1	1.088 (0.924 to 1.281)	0.3095	1.037 (0.879 to 1.223)	0.667	1.028 (0.875 to 1.209)	0.7347	1.024 (0.875 to 1.199)	0.7657	0.9388	0.990 (0.945 to 1.038)	0.6827
Women	Lung cancer	AIP	1	1.308 (1.076 to 1.592)	0.0072	1.467 (1.213 to 1.774)	<0.001	1.450 (1.201 to 1.751)	<0.001	1.520 (1.257 to 1.839)	<0.001	<0.001	1.110 (1.053 to 1.170)	<0.001
Women	Lung cancer	AC	1	1.018 (0.856 to 1.212)	0.837	1.128 (0.954 to 1.335)	0.1594	1.086 (0.916 to 1.286)	0.3422	1.173 (0.993 to 1.386)	0.0599	0.0454	1.045 (0.995 to 1.096)	0.0761
Women	Lung cancer	CRI-I	1	1.018 (0.856 to 1.212)	0.837	1.128 (0.954 to 1.335)	0.1594	1.086 (0.916 to 1.286)	0.3422	1.173 (0.993 to 1.386)	0.0599	0.0454	1.045 (0.995 to 1.096)	0.0761
Women	Lung cancer	CRI-II	1	1.037 (0.875 to 1.229)	0.6764	1.081 (0.915 to 1.278)	0.3582	1.084 (0.917 to 1.281)	0.3435	1.137 (0.964 to 1.340)	0.1278	0.1122	1.037 (0.987 to 1.089)	0.1457
Women	Lung cancer	LCI	1	1.194 (0.990 to 1.440)	0.0632	1.225 (1.019 to 1.473)	0.0307	1.292 (1.079 to 1.548)	0.0054	1.320 (1.104 to 1.579)	0.0024	0.0029	1.020 (0.980 to 1.061)	0.331
Women	Lung cancer	THDL	1	1.308 (1.076 to 1.592)	0.0072	1.467 (1.213 to 1.774)	<0.001	1.450 (1.201 to 1.751)	<0.001	1.520 (1.257 to 1.839)	<0.001	<0.001	1.055 (1.013 to 1.099)	0.0101
Women	Colon cancer	HDL-C	1	1.026 (0.890 to 1.182)	0.7277	0.983 (0.848 to 1.138)	0.8163	0.912 (0.781 to 1.064)	0.2418	1.038 (0.887 to 1.214)	0.6454	0.8204	1.012 (0.961 to 1.066)	0.6492
Women	Colon cancer	LDL-C	1	1.136 (0.978 to 1.320)	0.0952	1.036 (0.889 to 1.206)	0.6511	1.068 (0.919 to 1.241)	0.3896	1.123 (0.970 to 1.301)	0.1206	0.3036	1.046 (0.999 to 1.095)	0.054
Women	Colon cancer	TG	1	0.948 (0.806 to 1.116)	0.5203	1.052 (0.898 to 1.232)	0.5289	0.970 (0.827 to 1.138)	0.7084	1.131 (0.967 to 1.323)	0.1246	0.091	1.053 (1.010 to 1.098)	0.016
Women	Colon cancer	TC	1	1.081 (0.927 to 1.260)	0.3219	1.123 (0.965 to 1.307)	0.1334	1.104 (0.949 to 1.284)	0.1999	1.131 (0.974 to 1.312)	0.1071	0.1302	1.048 (1.001 to 1.097)	0.0464
Women	Colon cancer	NONHDL	1	1.123 (0.964 to 1.308)	0.1351	1.096 (0.941 to 1.276)	0.239	1.031 (0.885 to 1.202)	0.6923	1.190 (1.027 to 1.378)	0.0207	0.1068	1.046 (0.999 to 1.095)	0.0536
Women	Colon cancer	AIP	1	0.994 (0.849 to 1.164)	0.9403	0.946 (0.808 to 1.107)	0.4867	0.981 (0.839 to 1.148)	0.8145	1.097 (0.937 to 1.285)	0.2488	0.2572	1.045 (0.993 to 1.099)	0.0911
Women	Colon cancer	AC	1	0.979 (0.842 to 1.138)	0.7824	0.967 (0.833 to 1.124)	0.6658	0.956 (0.822 to 1.113)	0.5636	1.071 (0.921 to 1.244)	0.3737	0.458	1.031 (0.984 to 1.081)	0.2033
Women	Colon cancer	CRI-I	1	0.979 (0.842 to 1.138)	0.7824	0.967 (0.833 to 1.124)	0.6658	0.956 (0.822 to 1.113)	0.5636	1.071 (0.921 to 1.244)	0.3737	0.458	1.031 (0.984 to 1.081)	0.2033
Women	Colon cancer	CRI-II	1	0.949 (0.816 to 1.104)	0.4967	1.046 (0.903 to 1.212)	0.5492	0.966 (0.831 to 1.123)	0.6554	1.083 (0.933 to 1.257)	0.2953	0.2845	1.032 (0.985 to 1.083)	0.1858
Women	Colon cancer	LCI	1	1.118 (0.954 to 1.309)	0.1685	1.014 (0.864 to 1.190)	0.8653	1.087 (0.929 to 1.273)	0.2964	1.223 (1.048 to 1.428)	0.0106	0.0251	1.049 (1.009 to 1.090)	0.017
Women	Colon cancer	THDL	1	0.994 (0.849 to 1.164)	0.9403	0.946 (0.808 to 1.107)	0.4867	0.981 (0.839 to 1.148)	0.8145	1.097 (0.937 to 1.285)	0.2488	0.2572	1.039 (0.997 to 1.084)	0.0714
Women	Rectal cancer	HDL-C	1	1.301 (1.007 to 1.681)	0.0437	1.100 (0.840 to 1.442)	0.4876	1.317 (1.005 to 1.726)	0.0457	1.361 (1.029 to 1.800)	0.0307	0.0556	1.064 (0.980 to 1.156)	0.1415
Women	Rectal cancer	LDL-C	1	1.128 (0.874 to 1.457)	0.3541	0.998 (0.768 to 1.296)	0.988	1.147 (0.891 to 1.477)	0.2858	1.123 (0.872 to 1.446)	0.3704	0.3919	1.033 (0.956 to 1.117)	0.4093
Women	Rectal cancer	TG	1	0.861 (0.655 to 1.133)	0.2856	0.922 (0.705 to 1.207)	0.5544	1.093 (0.841 to 1.420)	0.5063	1.031 (0.788 to 1.349)	0.824	0.289	1.033 (0.956 to 1.116)	0.4121
Women	Rectal cancer	TC	1	0.966 (0.743 to 1.256)	0.7954	0.955 (0.735 to 1.239)	0.7279	1.120 (0.871 to 1.439)	0.377	1.109 (0.863 to 1.426)	0.4196	0.2056	1.058 (0.979 to 1.144)	0.1527

Gender	Cancer type	Lipid index	1st quintile	2nd quintile		3rd quintile		4th quintile		5th quintile		p-value for trend	Per 1 SD increase	
				aHR (95% CI)	p-value	aHR (95% CI)	p-value	aHR (95% CI)	p-value	aHR (95% CI)	p-value		aHR (95% CI)	p-value
Women	Rectal cancer	NONHDL	1	1.013 (0.780 to 1.315)	0.9221	1.058 (0.818 to 1.368)	0.6696	1.029 (0.796 to 1.330)	0.828	1.190 (0.929 to 1.526)	0.1691	0.1879	1.041 (0.964 to 1.125)	0.3032
Women	Rectal cancer	AIP	1	0.979 (0.752 to 1.274)	0.8752	1.024 (0.789 to 1.328)	0.8586	0.987 (0.755 to 1.290)	0.9251	0.977 (0.743 to 1.283)	0.8654	0.8928	1.008 (0.924 to 1.099)	0.8655
Women	Rectal cancer	AC	1	1.112 (0.865 to 1.429)	0.4094	1.077 (0.836 to 1.388)	0.5641	0.982 (0.755 to 1.276)	0.8893	1.016 (0.779 to 1.324)	0.9086	0.731	0.981 (0.905 to 1.063)	0.6347
Women	Rectal cancer	CRI-I	1	1.112 (0.865 to 1.429)	0.4094	1.077 (0.836 to 1.388)	0.5641	0.982 (0.755 to 1.276)	0.8893	1.016 (0.779 to 1.324)	0.9086	0.731	0.981 (0.905 to 1.063)	0.6347
Women	Rectal cancer	CRI-II	1	1.146 (0.894 to 1.468)	0.2819	1.035 (0.803 to 1.334)	0.7914	0.971 (0.748 to 1.260)	0.8247	1.021 (0.785 to 1.328)	0.878	0.655	0.972 (0.897 to 1.053)	0.4914
Women	Rectal cancer	LCI	1	1.059 (0.813 to 1.379)	0.671	1.010 (0.773 to 1.318)	0.9443	1.045 (0.800 to 1.365)	0.7457	1.115 (0.854 to 1.455)	0.4245	0.4893	0.998 (0.930 to 1.070)	0.9468
Women	Rectal cancer	THDL	1	0.979 (0.752 to 1.274)	0.8752	1.024 (0.789 to 1.328)	0.8586	0.987 (0.755 to 1.290)	0.9251	0.977 (0.743 to 1.283)	0.8654	0.8928	1.011 (0.929 to 1.099)	0.8001
Women	Stomach cancer	HDL-C	1	0.862 (0.595 to 1.247)	0.4301	0.724 (0.479 to 1.095)	0.126	0.749 (0.487 to 1.152)	0.1879	0.605 (0.375 to 0.977)	0.0397	0.0385	0.812 (0.693 to 0.952)	0.0103
Women	Stomach cancer	LDL-C	1	0.921 (0.606 to 1.398)	0.6988	1.048 (0.695 to 1.581)	0.8229	1.199 (0.811 to 1.772)	0.3629	0.790 (0.518 to 1.206)	0.2756	0.6819	0.969 (0.863 to 1.088)	0.5902
Women	Stomach cancer	TG	1	1.004 (0.632 to 1.593)	0.9878	0.927 (0.576 to 1.492)	0.7547	1.130 (0.713 to 1.790)	0.6037	0.970 (0.605 to 1.554)	0.8991	0.9144	0.958 (0.851 to 1.078)	0.475
Women	Stomach cancer	TC	1	0.903 (0.602 to 1.356)	0.6232	0.860 (0.566 to 1.306)	0.4791	1.030 (0.698 to 1.519)	0.8833	0.751 (0.496 to 1.139)	0.1782	0.3568	0.930 (0.824 to 1.051)	0.2459
Women	Stomach cancer	NONHDL	1	0.895 (0.584 to 1.372)	0.6104	1.260 (0.845 to 1.878)	0.2575	0.995 (0.658 to 1.504)	0.9799	0.956 (0.634 to 1.441)	0.8293	0.9683	0.979 (0.872 to 1.098)	0.7175
Women	Stomach cancer	AIP	1	1.170 (0.727 to 1.884)	0.5175	1.195 (0.738 to 1.936)	0.4693	1.224 (0.749 to 1.998)	0.42	1.328 (0.813 to 2.169)	0.2565	0.2883	1.077 (0.937 to 1.239)	0.2954
Women	Stomach cancer	AC	1	1.154 (0.715 to 1.862)	0.5575	1.554 (0.992 to 2.434)	0.0543	1.630 (1.032 to 2.573)	0.0361	1.390 (0.870 to 2.220)	0.1683	0.0717	1.081 (0.962 to 1.216)	0.1915
Women	Stomach cancer	CRI-I	1	1.154 (0.715 to 1.862)	0.5575	1.554 (0.992 to 2.434)	0.0543	1.630 (1.032 to 2.573)	0.0361	1.390 (0.870 to 2.220)	0.1683	0.0717	1.081 (0.962 to 1.216)	0.1915
Women	Stomach cancer	CRI-II	1	1.339 (0.835 to 2.147)	0.2261	1.557 (0.987 to 2.459)	0.0571	1.780 (1.127 to 2.812)	0.0134	1.465 (0.914 to 2.347)	0.1124	0.0584	1.084 (0.963 to 1.221)	0.1826
Women	Stomach cancer	LCI	1	1.042 (0.658 to 1.650)	0.8616	1.072 (0.681 to 1.686)	0.7637	1.209 (0.769 to 1.900)	0.411	1.096 (0.694 to 1.730)	0.6938	0.5385	0.947 (0.849 to 1.055)	0.3222
Women	Stomach cancer	THDL	1	1.170 (0.727 to 1.884)	0.5175	1.195 (0.738 to 1.936)	0.4693	1.224 (0.749 to 1.998)	0.42	1.328 (0.813 to 2.169)	0.2565	0.2883	0.999 (0.898 to 1.111)	0.9816
Women	Liver cancer	HDL-C	1	0.843 (0.558 to 1.272)	0.4151	1.248 (0.846 to 1.841)	0.2645	0.844 (0.534 to 1.333)	0.4662	1.144 (0.737 to 1.775)	0.5494	0.6026	0.978 (0.830 to 1.152)	0.7908
Women	Liver cancer	LDL-C	1	0.791 (0.520 to 1.203)	0.2728	0.874 (0.579 to 1.318)	0.5197	0.929 (0.625 to 1.382)	0.7177	0.780 (0.519 to 1.172)	0.2318	0.4429	0.945 (0.829 to 1.078)	0.4032
Women	Liver cancer	TG	1	1.304 (0.788 to 2.156)	0.302	1.043 (0.620 to 1.756)	0.8726	1.375 (0.843 to 2.242)	0.2017	1.292 (0.781 to 2.138)	0.3179	0.3405	1.055 (0.929 to 1.198)	0.4112
Women	Liver cancer	TC	1	0.632 (0.408 to 0.979)	0.0399	0.878 (0.590 to 1.308)	0.5234	0.865 (0.578 to 1.294)	0.4803	0.822 (0.553 to 1.223)	0.3335	0.7373	0.957 (0.836 to 1.095)	0.5231
Women	Liver cancer	NONHDL	1	0.868 (0.577 to 1.306)	0.4973	0.837 (0.555 to 1.260)	0.393	0.946 (0.638 to 1.404)	0.7841	0.724 (0.479 to 1.094)	0.1256	0.236	0.961 (0.841 to 1.098)	0.5604
Women	Liver cancer	AIP	1	1.135 (0.694 to 1.858)	0.6134	1.041 (0.634 to 1.707)	0.8743	1.099 (0.676 to 1.789)	0.7025	1.163 (0.707 to 1.913)	0.5518	0.628	1.092 (0.941 to 1.268)	0.2468
Women	Liver cancer	AC	1	1.020 (0.661 to 1.574)	0.9275	0.776 (0.493 to 1.221)	0.2724	1.001 (0.655 to 1.530)	0.996	0.900 (0.579 to 1.397)	0.6374	0.6784	1.024 (0.883 to 1.188)	0.7552

Gender	Cancer type	Lipid index	1st quintile	2nd quintile		3rd quintile		4th quintile		5th quintile		p-value for trend	Per 1 SD increase	
				aHR (95% CI)	p-value	aHR (95% CI)	p-value	aHR (95% CI)	p-value	aHR (95% CI)	p-value		aHR (95% CI)	p-value
Women	Liver cancer	CRI-I	1	1.020 (0.661 to 1.574)	0.9275	0.776 (0.493 to 1.221)	0.2724	1.001 (0.655 to 1.530)	0.996	0.900 (0.579 to 1.397)	0.6374	0.6784	1.024 (0.883 to 1.188)	0.7552
Women	Liver cancer	CRI-II	1	1.183 (0.773 to 1.810)	0.4394	0.909 (0.582 to 1.419)	0.6739	0.887 (0.569 to 1.383)	0.5966	0.984 (0.636 to 1.524)	0.943	0.5261	1.006 (0.869 to 1.165)	0.9362
Women	Liver cancer	LCI	1	1.285 (0.804 to 2.053)	0.2941	1.108 (0.686 to 1.789)	0.6753	1.260 (0.796 to 1.993)	0.3236	1.037 (0.644 to 1.669)	0.881	0.926	1.030 (0.904 to 1.173)	0.6609
Women	Liver cancer	THDL	1	1.135 (0.694 to 1.858)	0.6134	1.041 (0.634 to 1.707)	0.8743	1.099 (0.676 to 1.789)	0.7025	1.163 (0.707 to 1.913)	0.5518	0.628	1.090 (0.964 to 1.232)	0.1679
Women	Bladder cancer	HDL-C	1	1.026 (0.794 to 1.326)	0.8453	0.960 (0.736 to 1.253)	0.7648	1.153 (0.882 to 1.506)	0.2976	1.077 (0.811 to 1.431)	0.607	0.414	1.016 (0.928 to 1.111)	0.7347
Women	Bladder cancer	LDL-C	1	0.988 (0.754 to 1.296)	0.9313	1.077 (0.827 to 1.401)	0.5825	1.067 (0.824 to 1.382)	0.6227	1.108 (0.858 to 1.432)	0.4318	0.3424	1.030 (0.953 to 1.113)	0.4535
Women	Bladder cancer	TG	1	1.078 (0.801 to 1.452)	0.6184	1.145 (0.856 to 1.531)	0.361	1.059 (0.793 to 1.414)	0.6977	0.984 (0.732 to 1.323)	0.9142	0.6933	0.957 (0.885 to 1.035)	0.2694
Women	Bladder cancer	TC	1	0.964 (0.735 to 1.264)	0.7906	1.035 (0.793 to 1.350)	0.8026	1.171 (0.910 to 1.508)	0.2198	1.059 (0.818 to 1.372)	0.6621	0.3101	1.024 (0.949 to 1.105)	0.5477
Women	Bladder cancer	NONHDL	1	0.999 (0.761 to 1.311)	0.9936	1.013 (0.775 to 1.324)	0.924	1.063 (0.819 to 1.379)	0.6476	1.101 (0.852 to 1.423)	0.462	0.3775	1.020 (0.944 to 1.102)	0.6146
Women	Bladder cancer	AIP	1	1.125 (0.839 to 1.510)	0.4305	1.169 (0.876 to 1.560)	0.2893	1.091 (0.816 to 1.460)	0.5566	1.072 (0.794 to 1.448)	0.6509	0.8791	0.977 (0.893 to 1.067)	0.6017
Women	Bladder cancer	AC	1	0.921 (0.695 to 1.220)	0.5665	0.967 (0.734 to 1.274)	0.8131	1.292 (0.997 to 1.674)	0.0525	0.995 (0.754 to 1.312)	0.9708	0.2729	1.008 (0.930 to 1.093)	0.8501
Women	Bladder cancer	CRI-I	1	0.921 (0.695 to 1.220)	0.5665	0.967 (0.734 to 1.274)	0.8131	1.292 (0.997 to 1.674)	0.0525	0.995 (0.754 to 1.312)	0.9708	0.2729	1.008 (0.930 to 1.093)	0.8501
Women	Bladder cancer	CRI-II	1	0.920 (0.698 to 1.213)	0.5548	0.975 (0.743 to 1.277)	0.8517	1.166 (0.899 to 1.512)	0.2469	1.010 (0.771 to 1.325)	0.94	0.3969	1.017 (0.937 to 1.103)	0.691
Women	Bladder cancer	LCI	1	1.001 (0.747 to 1.341)	0.9962	1.102 (0.830 to 1.462)	0.5016	1.088 (0.821 to 1.441)	0.556	1.091 (0.822 to 1.449)	0.5463	0.4402	0.961 (0.895 to 1.032)	0.2787
Women	Bladder cancer	THDL	1	1.125 (0.839 to 1.510)	0.4305	1.169 (0.876 to 1.560)	0.2893	1.091 (0.816 to 1.460)	0.5566	1.072 (0.794 to 1.448)	0.6509	0.8791	0.951 (0.879 to 1.028)	0.2024
Women	Breast cancer	HDL	1	0.971 (0.912 to 1.034)	0.3618	0.989 (0.928 to 1.055)	0.7455	0.994 (0.931 to 1.061)	0.8521	0.973 (0.909 to 1.043)	0.4431	0.7075	1.000 (0.979 to 1.023)	0.9658
Women	Breast cancer	LDL	1	1.056 (0.991 to 1.126)	0.0915	1.015 (0.951 to 1.082)	0.6575	1.023 (0.959 to 1.090)	0.4951	1.092 (1.025 to 1.163)	0.0063	0.0426	1.027 (1.007 to 1.048)	0.0089
Women	Breast cancer	TG	1	1.069 (0.999 to 1.143)	0.0527	1.119 (1.047 to 1.197)	<0.001	1.114 (1.041 to 1.192)	0.0018	1.104 (1.030 to 1.183)	0.0051	0.0046	1.025 (1.005 to 1.046)	0.0149
Women	Breast cancer	TC	1	1.072 (1.005 to 1.143)	0.0347	1.044 (0.978 to 1.113)	0.197	1.025 (0.960 to 1.094)	0.459	1.097 (1.029 to 1.169)	0.0048	0.0557	1.027 (1.006 to 1.048)	0.0104
Women	Breast cancer	NONHDL	1	1.039 (0.974 to 1.107)	0.2453	1.012 (0.949 to 1.079)	0.7227	1.017 (0.954 to 1.084)	0.6114	1.090 (1.023 to 1.161)	0.0078	0.0329	1.027 (1.007 to 1.048)	0.0083
Women	Breast cancer	AIP	1	1.024 (0.958 to 1.093)	0.4869	1.055 (0.988 to 1.127)	0.1107	1.054 (0.986 to 1.127)	0.1229	1.071 (0.999 to 1.148)	0.0532	0.0409	1.027 (1.004 to 1.049)	0.0202
Women	Breast cancer	AC	1	1.012 (0.950 to 1.079)	0.705	1.000 (0.938 to 1.067)	0.9908	0.999 (0.936 to 1.066)	0.9686	1.059 (0.991 to 1.130)	0.0888	0.1742	1.026 (1.004 to 1.047)	0.0178
Women	Breast cancer	CRI1	1	1.012 (0.950 to 1.079)	0.705	1.000 (0.938 to 1.067)	0.9908	0.999 (0.936 to 1.066)	0.9686	1.059 (0.991 to 1.130)	0.0888	0.1742	1.026 (1.004 to 1.047)	0.0178
Women	Breast cancer	CRI2	1	1.038 (0.974 to 1.106)	0.2506	1.014 (0.951 to 1.081)	0.6799	0.978 (0.916 to 1.044)	0.5055	1.083 (1.015 to 1.156)	0.016	0.1645	1.026 (1.005 to 1.048)	0.0167
Women	Breast cancer	LCI	1	1.044 (0.977 to 1.115)	0.2037	1.079 (1.010 to 1.153)	0.0238	1.058 (0.989 to 1.131)	0.0996	1.095 (1.024 to 1.172)	0.0083	0.014	1.033 (1.014 to 1.053)	<0.001

Gender	Cancer type	Lipid index	1st quintile	2nd quintile		3rd quintile		4th quintile		5th quintile		p-value for trend	Per 1 SD increase	
				aHR (95% CI)	p-value	aHR (95% CI)	p-value	aHR (95% CI)	p-value	aHR (95% CI)	p-value		aHR (95% CI)	p-value
Women	Breast cancer	THDL	1	1.024 (0.958 to 1.093)	0.4869	1.055 (0.988 to 1.127)	0.1107	1.054 (0.986 to 1.127)	0.1229	1.071 (0.999 to 1.148)	0.0532	0.0409	1.021 (1.001 to 1.042)	0.0439
Women	Cervix uteri cancer	HDL-C	1	1.233 (0.830 to 1.829)	0.2994	1.040 (0.677 to 1.598)	0.8569	1.229 (0.801 to 1.886)	0.3458	1.521 (0.990 to 2.338)	0.0558	0.1031	1.167 (1.019 to 1.337)	0.0252
Women	Cervix uteri cancer	LDL-C	1	1.337 (0.904 to 1.979)	0.1462	1.045 (0.685 to 1.596)	0.837	1.300 (0.867 to 1.950)	0.2036	1.068 (0.693 to 1.644)	0.7667	0.8268	1.007 (0.890 to 1.140)	0.9079
Women	Cervix uteri cancer	TG	1	0.695 (0.455 to 1.061)	0.0916	1.042 (0.706 to 1.538)	0.836	0.835 (0.546 to 1.276)	0.4048	1.069 (0.715 to 1.598)	0.746	0.5383	1.040 (0.922 to 1.172)	0.525
Women	Cervix uteri cancer	TC	1	1.012 (0.659 to 1.555)	0.9554	1.554 (1.048 to 2.305)	0.0284	1.423 (0.943 to 2.147)	0.0926	1.111 (0.710 to 1.738)	0.6449	0.2283	1.066 (0.947 to 1.201)	0.2907
Women	Cervix uteri cancer	NONHDL	1	1.237 (0.832 to 1.840)	0.2935	1.155 (0.763 to 1.747)	0.4963	1.239 (0.824 to 1.861)	0.3028	1.071 (0.698 to 1.644)	0.7537	0.7639	1.020 (0.902 to 1.154)	0.7475
Women	Cervix uteri cancer	AIP	1	1.009 (0.678 to 1.501)	0.9643	0.950 (0.635 to 1.421)	0.8035	0.708 (0.454 to 1.105)	0.1281	1.107 (0.730 to 1.677)	0.633	0.8917	0.980 (0.854 to 1.125)	0.7768
Women	Cervix uteri cancer	AC	1	1.098 (0.750 to 1.609)	0.6306	0.626 (0.404 to 0.969)	0.0354	0.956 (0.642 to 1.424)	0.8255	0.896 (0.598 to 1.342)	0.5941	0.4467	0.955 (0.834 to 1.093)	0.5037
Women	Cervix uteri cancer	CRI-I	1	1.098 (0.750 to 1.609)	0.6306	0.626 (0.404 to 0.969)	0.0354	0.956 (0.642 to 1.424)	0.8255	0.896 (0.598 to 1.342)	0.5941	0.4467	0.955 (0.834 to 1.093)	0.5037
Women	Cervix uteri cancer	CRI-II	1	1.212 (0.836 to 1.758)	0.3096	0.617 (0.397 to 0.959)	0.0318	0.920 (0.616 to 1.373)	0.6825	0.914 (0.615 to 1.359)	0.6558	0.3101	0.942 (0.822 to 1.080)	0.3921
Women	Cervix uteri cancer	LCI	1	0.865 (0.577 to 1.298)	0.4847	1.167 (0.790 to 1.723)	0.4377	0.831 (0.544 to 1.269)	0.3907	0.983 (0.656 to 1.474)	0.9344	0.8815	1.008 (0.906 to 1.121)	0.8898
Women	Cervix uteri cancer	THDL	1	1.009 (0.678 to 1.501)	0.9643	0.950 (0.635 to 1.421)	0.8035	0.708 (0.454 to 1.105)	0.1281	1.107 (0.730 to 1.677)	0.633	0.8917	0.979 (0.867 to 1.105)	0.7257
Women	Thyroid cancer	HDL-C	1	1.009 (0.728 to 1.398)	0.9561	0.964 (0.678 to 1.369)	0.8357	0.814 (0.561 to 1.181)	0.2786	0.745 (0.488 to 1.136)	0.1713	0.1022	0.878 (0.769 to 1.002)	0.0532
Women	Thyroid cancer	LDL-C	1	0.706 (0.492 to 1.014)	0.0594	0.792 (0.555 to 1.128)	0.1962	1.112 (0.798 to 1.549)	0.5309	0.879 (0.614 to 1.257)	0.4784	0.7328	1.015 (0.903 to 1.141)	0.8001
Women	Thyroid cancer	TG	1	1.247 (0.856 to 1.817)	0.2508	1.055 (0.708 to 1.573)	0.7914	1.152 (0.779 to 1.704)	0.4784	1.289 (0.865 to 1.922)	0.2126	0.3478	1.054 (0.944 to 1.176)	0.349
Women	Thyroid cancer	TC	1	0.729 (0.508 to 1.046)	0.0858	0.872 (0.612 to 1.241)	0.4466	1.008 (0.716 to 1.419)	0.9644	0.962 (0.671 to 1.378)	0.8319	0.6817	1.002 (0.888 to 1.131)	0.9683
Women	Thyroid cancer	NONHDL	1	0.816 (0.568 to 1.172)	0.2707	0.805 (0.556 to 1.167)	0.252	1.203 (0.856 to 1.690)	0.2867	1.064 (0.746 to 1.515)	0.733	0.2508	1.038 (0.923 to 1.167)	0.5339
Women	Thyroid cancer	AIP	1	1.184 (0.795 to 1.763)	0.4069	1.371 (0.924 to 2.036)	0.1172	1.249 (0.829 to 1.882)	0.2877	1.367 (0.901 to 2.073)	0.1416	0.1709	1.093 (0.967 to 1.236)	0.1545
Women	Thyroid cancer	AC	1	0.962 (0.652 to 1.419)	0.8458	1.159 (0.792 to 1.695)	0.4483	1.291 (0.888 to 1.876)	0.1803	1.263 (0.864 to 1.848)	0.2285	0.0901	1.083 (0.969 to 1.210)	0.1608
Women	Thyroid cancer	CRI-I	1	0.962 (0.652 to 1.419)	0.8458	1.159 (0.792 to 1.695)	0.4483	1.291 (0.888 to 1.876)	0.1803	1.263 (0.864 to 1.848)	0.2285	0.0901	1.083 (0.969 to 1.210)	0.1608
Women	Thyroid cancer	CRI-II	1	1.084 (0.731 to 1.607)	0.6879	1.195 (0.811 to 1.761)	0.3679	1.487 (1.021 to 2.164)	0.0384	1.305 (0.884 to 1.926)	0.1806	0.0556	1.069 (0.958 to 1.194)	0.2337
Women	Thyroid cancer	LCI	1	0.803 (0.547 to 1.178)	0.2621	1.028 (0.708 to 1.494)	0.8836	1.153 (0.793 to 1.676)	0.4555	1.120 (0.773 to 1.622)	0.5503	0.2007	1.062 (0.955 to 1.182)	0.2662
Women	Thyroid cancer	THDL	1	1.184 (0.795 to 1.763)	0.4069	1.371 (0.924 to 2.036)	0.1172	1.249 (0.829 to 1.882)	0.2877	1.367 (0.901 to 2.073)	0.1416	0.1709	1.069 (0.963 to 1.186)	0.2092
Women	Pancreas cancer	HDL-C	1	0.887 (0.668 to 1.179)	0.41	0.888 (0.658 to 1.200)	0.4401	0.997 (0.733 to 1.354)	0.9822	1.153 (0.847 to 1.570)	0.3665	0.3169	1.052 (0.953 to 1.161)	0.3177
Women	Pancreas cancer	LDL-C	1	1.078 (0.796 to 1.458)	0.6283	1.086 (0.799 to 1.477)	0.5992	1.346 (1.005 to 1.803)	0.0464	1.196 (0.894 to 1.600)	0.229	0.0862	1.050 (0.962 to 1.146)	0.2707

Gender	Cancer type	Lipid index	1st quintile	2nd quintile		3rd quintile		4th quintile		5th quintile		p-value for trend	Per 1 SD increase	
				aHR (95% CI)	p-value	aHR (95% CI)	p-value	aHR (95% CI)	p-value	aHR (95% CI)	p-value		aHR (95% CI)	p-value
Women	Pancreas cancer	TG	1	1.184 (0.838 to 1.672)	0.3382	1.252 (0.895 to 1.752)	0.1898	1.111 (0.789 to 1.565)	0.5458	1.182 (0.843 to 1.658)	0.3332	0.6075	1.001 (0.913 to 1.099)	0.9769
Women	Pancreas cancer	TC	1	1.194 (0.884 to 1.613)	0.2474	1.070 (0.779 to 1.469)	0.6765	1.370 (1.019 to 1.841)	0.037	1.338 (0.999 to 1.793)	0.0509	0.0318	1.061 (0.972 to 1.158)	0.1846
Women	Pancreas cancer	NONHDL	1	1.012 (0.747 to 1.373)	0.9373	1.152 (0.855 to 1.553)	0.3514	1.205 (0.897 to 1.618)	0.2162	1.187 (0.890 to 1.583)	0.2425	0.1291	1.049 (0.962 to 1.143)	0.282
Women	Pancreas cancer	AIP	1	1.148 (0.832 to 1.584)	0.4003	1.127 (0.817 to 1.554)	0.4679	0.987 (0.714 to 1.365)	0.9376	1.019 (0.733 to 1.416)	0.9114	0.6665	0.996 (0.902 to 1.100)	0.936
Women	Pancreas cancer	AC	1	0.865 (0.637 to 1.174)	0.351	0.949 (0.706 to 1.275)	0.7275	0.996 (0.742 to 1.336)	0.9787	0.955 (0.712 to 1.282)	0.7591	0.8834	1.013 (0.924 to 1.111)	0.7791
Women	Pancreas cancer	CRI-I	1	0.865 (0.637 to 1.174)	0.351	0.949 (0.706 to 1.275)	0.7275	0.996 (0.742 to 1.336)	0.9787	0.955 (0.712 to 1.282)	0.7591	0.8834	1.013 (0.924 to 1.111)	0.7791
Women	Pancreas cancer	CRI-II	1	0.927 (0.686 to 1.251)	0.62	1.013 (0.757 to 1.356)	0.9312	0.981 (0.729 to 1.319)	0.8967	1.010 (0.755 to 1.352)	0.9446	0.8193	1.012 (0.923 to 1.110)	0.8001
Women	Pancreas cancer	LCI	1	1.116 (0.801 to 1.556)	0.5157	1.296 (0.944 to 1.781)	0.1088	1.118 (0.807 to 1.549)	0.5021	1.164 (0.845 to 1.604)	0.3534	0.4935	1.017 (0.930 to 1.112)	0.7107
Women	Pancreas cancer	THDL	1	1.148 (0.832 to 1.584)	0.4003	1.127 (0.817 to 1.554)	0.4679	0.987 (0.714 to 1.365)	0.9376	1.019 (0.733 to 1.416)	0.9114	0.6665	0.990 (0.900 to 1.090)	0.8397
Women	Ovarian cancer	HDL-C	1	1.179 (0.974 to 1.426)	0.0916	0.972 (0.793 to 1.193)	0.7887	1.038 (0.842 to 1.280)	0.7272	1.147 (0.929 to 1.417)	0.203	0.5808	1.018 (0.953 to 1.088)	0.5882
Women	Ovarian cancer	LDL-C	1	1.057 (0.867 to 1.289)	0.5831	0.951 (0.775 to 1.168)	0.6324	0.993 (0.811 to 1.215)	0.9447	1.155 (0.951 to 1.404)	0.1462	0.2784	1.063 (0.996 to 1.135)	0.066
Women	Ovarian cancer	TG	1	1.114 (0.901 to 1.377)	0.3194	1.079 (0.872 to 1.336)	0.4849	1.174 (0.949 to 1.453)	0.139	1.131 (0.909 to 1.406)	0.2692	0.2588	1.026 (0.964 to 1.091)	0.423
Women	Ovarian cancer	TC	1	1.067 (0.872 to 1.304)	0.5298	1.061 (0.865 to 1.301)	0.5675	1.052 (0.859 to 1.289)	0.6219	1.132 (0.927 to 1.383)	0.2243	0.3048	1.069 (1.001 to 1.141)	0.0464
Women	Ovarian cancer	NONHDL	1	1.037 (0.848 to 1.267)	0.7242	0.969 (0.791 to 1.188)	0.7632	0.974 (0.794 to 1.195)	0.801	1.170 (0.961 to 1.424)	0.1188	0.2278	1.066 (0.998 to 1.138)	0.0563
Women	Ovarian cancer	AIP	1	1.006 (0.818 to 1.237)	0.955	1.004 (0.817 to 1.233)	0.972	1.018 (0.827 to 1.253)	0.8657	1.005 (0.811 to 1.246)	0.9614	0.9316	1.018 (0.950 to 1.091)	0.6109
Women	Ovarian cancer	AC	1	0.999 (0.819 to 1.218)	0.9906	0.896 (0.731 to 1.098)	0.2906	0.888 (0.724 to 1.091)	0.2584	1.089 (0.891 to 1.331)	0.4045	0.7151	1.037 (0.971 to 1.107)	0.2834
Women	Ovarian cancer	CRI-I	1	0.999 (0.819 to 1.218)	0.9906	0.896 (0.731 to 1.098)	0.2906	0.888 (0.724 to 1.091)	0.2584	1.089 (0.891 to 1.331)	0.4045	0.7151	1.037 (0.971 to 1.107)	0.2834
Women	Ovarian cancer	CRI-II	1	1.013 (0.831 to 1.234)	0.9	0.877 (0.715 to 1.076)	0.2091	0.908 (0.740 to 1.113)	0.3518	1.118 (0.917 to 1.363)	0.2708	0.531	1.034 (0.969 to 1.103)	0.3141
Women	Ovarian cancer	LCI	1	1.125 (0.913 to 1.387)	0.2691	1.032 (0.836 to 1.275)	0.7691	1.149 (0.932 to 1.417)	0.1944	1.161 (0.939 to 1.436)	0.1668	0.1927	1.049 (0.990 to 1.111)	0.1081
Women	Ovarian cancer	THDL	1	1.006 (0.818 to 1.237)	0.955	1.004 (0.817 to 1.233)	0.972	1.018 (0.827 to 1.253)	0.8657	1.005 (0.811 to 1.246)	0.9614	0.9316	1.015 (0.953 to 1.082)	0.6333
Men	Overall cancer	HDL-C	1	0.961 (0.926 to 0.998)	0.0376	0.957 (0.921 to 0.994)	0.0229	0.965 (0.928 to 1.003)	0.0719	0.987 (0.948 to 1.028)	0.5345	0.6446	1.006 (0.993 to 1.019)	0.4016
Men	Overall cancer	LDL-C	1	1.083 (1.045 to 1.123)	<0.001	1.084 (1.044 to 1.125)	<0.001	1.085 (1.045 to 1.127)	<0.001	1.098 (1.057 to 1.141)	<0.001	<0.001	1.028 (1.016 to 1.041)	<0.001
Men	Overall cancer	TG	1	1.003 (0.966 to 1.041)	0.8762	1.001 (0.964 to 1.040)	0.944	1.028 (0.989 to 1.067)	0.1592	1.013 (0.974 to 1.054)	0.5095	0.2481	0.999 (0.987 to 1.012)	0.9086
Men	Overall cancer	TC	1	1.077 (1.038 to 1.116)	<0.001	1.055 (1.016 to 1.096)	0.0051	1.113 (1.072 to 1.156)	<0.001	1.098 (1.057 to 1.141)	<0.001	<0.001	1.028 (1.016 to 1.040)	<0.001
Men	Overall cancer	NONHDL	1	1.060 (1.023 to 1.099)	0.0014	1.073 (1.034 to 1.113)	<0.001	1.084 (1.044 to 1.125)	<0.001	1.088 (1.047 to 1.131)	<0.001	<0.001	1.028 (1.015 to 1.040)	<0.001

Gender	Cancer type	Lipid index	1st quintile	2nd quintile		3rd quintile		4th quintile		5th quintile		p-value for trend	Per 1 SD increase	
				aHR (95% CI)	p-value	aHR (95% CI)	p-value	aHR (95% CI)	p-value	aHR (95% CI)	p-value		aHR (95% CI)	p-value
Men	Overall cancer	AIP	1	0.966 (0.930 to 1.003)	0.0744	0.997 (0.960 to 1.036)	0.8967	1.010 (0.972 to 1.050)	0.6096	0.989 (0.951 to 1.030)	0.6036	0.5762	1.001 (0.988 to 1.014)	0.9094
Men	Overall cancer	AC	1	1.013 (0.977 to 1.050)	0.4799	1.008 (0.972 to 1.046)	0.6521	1.052 (1.013 to 1.092)	0.0078	1.077 (1.036 to 1.120)	<0.001	<0.001	1.026 (1.013 to 1.039)	<0.001
Men	Overall cancer	CRI-I	1	1.013 (0.977 to 1.050)	0.4799	1.008 (0.972 to 1.046)	0.6521	1.052 (1.013 to 1.092)	0.0078	1.077 (1.036 to 1.120)	<0.001	<0.001	1.026 (1.013 to 1.039)	<0.001
Men	Overall cancer	CRI-II	1	1.014 (0.978 to 1.051)	0.455	1.022 (0.985 to 1.060)	0.2488	1.047 (1.008 to 1.087)	0.0173	1.077 (1.036 to 1.120)	<0.001	<0.001	1.027 (1.014 to 1.040)	<0.001
Men	Overall cancer	LCI	1	1.040 (1.003 to 1.078)	0.0361	1.042 (1.005 to 1.081)	0.0271	1.033 (0.995 to 1.072)	0.0931	1.070 (1.029 to 1.112)	<0.001	0.004	1.011 (0.998 to 1.023)	0.0958
Men	Overall cancer	THDL	1	0.966 (0.930 to 1.003)	0.0744	0.997 (0.960 to 1.036)	0.8967	1.010 (0.972 to 1.050)	0.6096	0.989 (0.951 to 1.030)	0.6036	0.5762	1.001 (0.988 to 1.013)	0.9053
Men	Overall cancer*	HDL-C	1	0.965 (0.929 to 1.001)	0.0595	0.959 (0.923 to 0.996)	0.0319	0.967 (0.930 to 1.005)	0.0891	0.989 (0.950 to 1.029)	0.5754	0.657	1.006 (0.993 to 1.019)	0.3866
Men	Overall cancer*	LDL-C	1	1.082 (1.044 to 1.122)	<0.001	1.083 (1.044 to 1.124)	<0.001	1.085 (1.045 to 1.127)	<0.001	1.099 (1.057 to 1.141)	<0.001	<0.001	1.029 (1.016 to 1.041)	<0.001
Men	Overall cancer*	TG	1	1.003 (0.966 to 1.042)	0.8681	1.003 (0.965 to 1.041)	0.894	1.029 (0.991 to 1.069)	0.1358	1.014 (0.975 to 1.055)	0.4744	0.2158	1.000 (0.987 to 1.012)	0.9802
Men	Overall cancer*	TC	1	1.077 (1.038 to 1.117)	<0.001	1.055 (1.016 to 1.096)	0.0051	1.113 (1.072 to 1.156)	<0.001	1.100 (1.058 to 1.143)	<0.001	<0.001	1.028 (1.016 to 1.041)	<0.001
Men	Overall cancer*	NONHDL	1	1.060 (1.022 to 1.098)	0.0016	1.072 (1.033 to 1.113)	<0.001	1.083 (1.043 to 1.125)	<0.001	1.089 (1.048 to 1.131)	<0.001	<0.001	1.028 (1.016 to 1.040)	<0.001
Men	Overall cancer*	AIP	1	0.967 (0.931 to 1.004)	0.0781	0.999 (0.962 to 1.038)	0.9596	1.012 (0.974 to 1.052)	0.5339	0.990 (0.951 to 1.030)	0.6186	0.5367	1.001 (0.988 to 1.014)	0.8707
Men	Overall cancer*	AC	1	1.015 (0.979 to 1.052)	0.4224	1.011 (0.974 to 1.049)	0.5661	1.054 (1.016 to 1.095)	0.0057	1.077 (1.036 to 1.120)	<0.001	<0.001	1.026 (1.013 to 1.039)	<0.001
Men	Overall cancer*	CRI-I	1	1.015 (0.979 to 1.052)	0.4224	1.011 (0.974 to 1.049)	0.5661	1.054 (1.016 to 1.095)	0.0057	1.077 (1.036 to 1.120)	<0.001	<0.001	1.026 (1.013 to 1.039)	<0.001
Men	Overall cancer*	CRI-II	1	1.016 (0.980 to 1.053)	0.3837	1.023 (0.986 to 1.061)	0.2262	1.049 (1.010 to 1.089)	0.0126	1.077 (1.036 to 1.120)	<0.001	<0.001	1.027 (1.014 to 1.040)	<0.001
Men	Overall cancer*	LCI	1	1.039 (1.001 to 1.077)	0.0415	1.044 (1.006 to 1.083)	0.0227	1.035 (0.996 to 1.074)	0.0771	1.069 (1.029 to 1.112)	<0.001	0.0033	1.011 (0.999 to 1.024)	0.0838
Men	Overall cancer*	THDL	1	0.967 (0.931 to 1.004)	0.0781	0.999 (0.962 to 1.038)	0.9596	1.012 (0.974 to 1.052)	0.5339	0.990 (0.951 to 1.030)	0.6186	0.5367	1.001 (0.989 to 1.014)	0.8712
Men	Lung cancer	HDL-C	1	0.988 (0.854 to 1.143)	0.8726	0.959 (0.822 to 1.119)	0.5939	0.901 (0.766 to 1.060)	0.2079	1.031 (0.874 to 1.216)	0.7196	0.8504	1.035 (0.980 to 1.092)	0.2173
Men	Lung cancer	LDL-C	1	1.013 (0.883 to 1.163)	0.8503	0.958 (0.826 to 1.112)	0.5767	0.952 (0.817 to 1.109)	0.5258	0.813 (0.692 to 0.955)	0.012	0.0146	0.948 (0.902 to 0.996)	0.0336
Men	Lung cancer	TG	1	0.977 (0.838 to 1.140)	0.7708	0.863 (0.736 to 1.013)	0.0709	1.032 (0.884 to 1.206)	0.6886	0.939 (0.800 to 1.101)	0.4364	0.7286	0.999 (0.950 to 1.051)	0.9796
Men	Lung cancer	TC	1	0.923 (0.801 to 1.062)	0.2628	0.912 (0.785 to 1.059)	0.2263	0.956 (0.822 to 1.113)	0.5631	0.835 (0.712 to 0.978)	0.0255	0.0706	0.961 (0.914 to 1.009)	0.1091
Men	Lung cancer	NONHDL	1	1.035 (0.902 to 1.187)	0.6272	0.923 (0.794 to 1.072)	0.2934	0.946 (0.812 to 1.101)	0.4725	0.865 (0.739 to 1.013)	0.072	0.0426	0.951 (0.905 to 0.999)	0.0454
Men	Lung cancer	AIP	1	0.951 (0.811 to 1.115)	0.5366	0.986 (0.840 to 1.156)	0.858	0.999 (0.851 to 1.172)	0.9884	0.956 (0.810 to 1.128)	0.595	0.8345	0.987 (0.935 to 1.042)	0.6356
Men	Lung cancer	AC	1	0.951 (0.824 to 1.097)	0.4909	0.902 (0.777 to 1.047)	0.1746	0.864 (0.740 to 1.010)	0.0661	0.909 (0.777 to 1.064)	0.2359	0.1061	0.961 (0.913 to 1.012)	0.1335
Men	Lung cancer	CRI-I	1	0.951 (0.824 to 1.097)	0.4909	0.902 (0.777 to 1.047)	0.1746	0.864 (0.740 to 1.010)	0.0661	0.909 (0.777 to 1.064)	0.2359	0.1061	0.961 (0.913 to 1.012)	0.1335

Gender	Cancer type	Lipid index	1st quintile	2nd quintile		3rd quintile		4th quintile		5th quintile		p-value for trend	Per 1 SD increase	
				aHR (95% CI)	p-value	aHR (95% CI)	p-value	aHR (95% CI)	p-value	aHR (95% CI)	p-value		aHR (95% CI)	p-value
Men	Lung cancer	CRI-II	1	0.856 (0.742 to 0.988)	0.0332	0.883 (0.762 to 1.022)	0.0955	0.821 (0.703 to 0.959)	0.0131	0.871 (0.746 to 1.017)	0.0798	0.0591	0.958 (0.909 to 1.008)	0.0989
Men	Lung cancer	LCI	1	1.055 (0.912 to 1.220)	0.4702	1.067 (0.921 to 1.237)	0.3857	0.882 (0.753 to 1.033)	0.1204	0.984 (0.841 to 1.152)	0.8447	0.2641	0.976 (0.926 to 1.028)	0.3616
Men	Colon cancer	THDL	1	0.951 (0.811 to 1.115)	0.5366	0.986 (0.840 to 1.156)	0.858	0.999 (0.851 to 1.172)	0.9884	0.956 (0.810 to 1.128)	0.595	0.8345	0.995 (0.947 to 1.045)	0.8313
Men	Colon cancer	HDL-C	1	0.895 (0.786 to 1.019)	0.0944	0.929 (0.815 to 1.060)	0.2737	0.872 (0.760 to 1.000)	0.0499	0.914 (0.795 to 1.051)	0.208	0.1943	0.991 (0.945 to 1.038)	0.6939
Men	Colon cancer	LDL-C	1	1.021 (0.897 to 1.162)	0.7516	1.062 (0.929 to 1.212)	0.3785	1.141 (1.000 to 1.303)	0.0507	1.196 (1.047 to 1.366)	0.0085	0.0026	1.072 (1.027 to 1.119)	0.0014
Men	Colon cancer	TG	1	1.005 (0.877 to 1.153)	0.9414	1.000 (0.872 to 1.147)	0.9951	0.994 (0.866 to 1.140)	0.9287	1.163 (1.015 to 1.332)	0.0298	0.0534	1.061 (1.017 to 1.106)	0.0056
Men	Colon cancer	TC	1	1.017 (0.893 to 1.158)	0.7973	1.043 (0.911 to 1.194)	0.5437	1.183 (1.037 to 1.349)	0.0124	1.178 (1.031 to 1.348)	0.0164	0.0021	1.079 (1.034 to 1.126)	<0.001
Men	Colon cancer	NONHDL	1	1.019 (0.894 to 1.160)	0.7797	1.071 (0.937 to 1.223)	0.3169	1.238 (1.086 to 1.411)	0.0014	1.179 (1.030 to 1.349)	0.0169	<0.001	1.085 (1.039 to 1.132)	<0.001
Men	Colon cancer	AIP	1	0.902 (0.786 to 1.036)	0.1461	0.956 (0.833 to 1.097)	0.52	0.985 (0.859 to 1.129)	0.8238	1.124 (0.979 to 1.290)	0.096	0.0339	1.046 (0.999 to 1.097)	0.0578
Men	Colon cancer	AC	1	0.906 (0.795 to 1.034)	0.1422	1.018 (0.894 to 1.160)	0.7856	1.058 (0.926 to 1.208)	0.4089	1.222 (1.070 to 1.396)	0.0032	<0.001	1.082 (1.036 to 1.130)	<0.001
Men	Colon cancer	CRI-I	1	0.906 (0.795 to 1.034)	0.1422	1.018 (0.894 to 1.160)	0.7856	1.058 (0.926 to 1.208)	0.4089	1.222 (1.070 to 1.396)	0.0032	<0.001	1.082 (1.036 to 1.130)	<0.001
Men	Colon cancer	CRI-II	1	0.893 (0.784 to 1.017)	0.088	0.977 (0.858 to 1.113)	0.7254	1.076 (0.944 to 1.228)	0.2733	1.177 (1.030 to 1.343)	0.0163	0.0019	1.074 (1.028 to 1.122)	0.0014
Men	Colon cancer	LCI	1	1.065 (0.934 to 1.215)	0.3449	1.042 (0.911 to 1.191)	0.5499	1.072 (0.937 to 1.227)	0.3108	1.270 (1.111 to 1.453)	<0.001	0.0021	1.077 (1.036 to 1.119)	<0.001
Men	Rectal cancer	THDL	1	0.902 (0.786 to 1.036)	0.1461	0.956 (0.833 to 1.097)	0.52	0.985 (0.859 to 1.129)	0.8238	1.124 (0.979 to 1.290)	0.096	0.0339	1.060 (1.018 to 1.103)	0.0044
Men	Rectal cancer	HDL-C	1	0.937 (0.766 to 1.146)	0.5239	1.141 (0.938 to 1.388)	0.1856	1.160 (0.950 to 1.415)	0.1452	1.146 (0.936 to 1.404)	0.1873	0.0392	1.087 (1.018 to 1.160)	0.0124
Men	Rectal cancer	LDL-C	1	1.083 (0.899 to 1.305)	0.4016	1.085 (0.893 to 1.318)	0.4105	1.180 (0.973 to 1.432)	0.093	1.284 (1.059 to 1.558)	0.0111	0.009	1.095 (1.030 to 1.165)	0.0035
Men	Rectal cancer	TG	1	0.987 (0.813 to 1.198)	0.8927	0.996 (0.821 to 1.209)	0.9703	1.117 (0.923 to 1.352)	0.2554	0.981 (0.802 to 1.200)	0.8515	0.6706	1.022 (0.958 to 1.089)	0.5113
Men	Rectal cancer	TC	1	1.122 (0.930 to 1.353)	0.2288	1.153 (0.948 to 1.403)	0.1533	1.179 (0.969 to 1.435)	0.0993	1.378 (1.135 to 1.672)	0.0012	0.0021	1.112 (1.046 to 1.183)	<0.001
Men	Rectal cancer	NONHDL	1	1.027 (0.851 to 1.238)	0.7843	1.084 (0.895 to 1.314)	0.4086	1.192 (0.985 to 1.443)	0.0715	1.206 (0.993 to 1.464)	0.059	0.0196	1.093 (1.028 to 1.162)	0.0047
Men	Rectal cancer	AIP	1	0.784 (0.645 to 0.952)	0.0142	0.928 (0.769 to 1.121)	0.4405	0.945 (0.781 to 1.144)	0.5625	0.909 (0.746 to 1.107)	0.3405	0.9701	0.985 (0.922 to 1.053)	0.6546
Men	Rectal cancer	AC	1	1.042 (0.871 to 1.247)	0.652	0.980 (0.813 to 1.181)	0.8295	1.003 (0.829 to 1.213)	0.9773	1.105 (0.909 to 1.343)	0.3163	0.5152	1.037 (0.972 to 1.106)	0.2695
Men	Rectal cancer	CRI-I	1	1.042 (0.871 to 1.247)	0.652	0.980 (0.813 to 1.181)	0.8295	1.003 (0.829 to 1.213)	0.9773	1.105 (0.909 to 1.343)	0.3163	0.5152	1.037 (0.972 to 1.106)	0.2695
Men	Rectal cancer	CRI-II	1	1.125 (0.942 to 1.343)	0.1941	0.951 (0.786 to 1.150)	0.6032	1.124 (0.931 to 1.357)	0.2224	1.081 (0.888 to 1.316)	0.439	0.4973	1.036 (0.972 to 1.104)	0.277
Men	Rectal cancer	LCI	1	1.042 (0.863 to 1.258)	0.6713	1.134 (0.940 to 1.367)	0.1891	0.999 (0.821 to 1.215)	0.9898	1.151 (0.945 to 1.403)	0.1632	0.2887	1.057 (0.993 to 1.124)	0.0804
Men	Stomach cancer	THDL	1	0.784 (0.645 to 0.952)	0.0142	0.928 (0.769 to 1.121)	0.4405	0.945 (0.781 to 1.144)	0.5625	0.909 (0.746 to 1.107)	0.3405	0.9701	1.006 (0.942 to 1.075)	0.8563

Gender	Cancer type	Lipid index	1st quintile	2nd quintile		3rd quintile		4th quintile		5th quintile		p-value for trend	Per 1 SD increase	
				aHR (95% CI)	p-value	aHR (95% CI)	p-value	aHR (95% CI)	p-value	aHR (95% CI)	p-value		aHR (95% CI)	p-value
Men	Stomach cancer	HDL-C	1	0.868 (0.672 to 1.120)	0.2767	0.955 (0.737 to 1.239)	0.7302	0.872 (0.660 to 1.152)	0.334	0.899 (0.669 to 1.208)	0.4804	0.5121	0.991 (0.892 to 1.101)	0.8668
Men	Stomach cancer	LDL-C	1	1.130 (0.880 to 1.452)	0.3383	1.022 (0.780 to 1.340)	0.8726	1.169 (0.896 to 1.526)	0.2495	0.882 (0.659 to 1.180)	0.3971	0.6502	0.973 (0.897 to 1.056)	0.5143
Men	Stomach cancer	TG	1	1.061 (0.797 to 1.411)	0.6859	1.070 (0.807 to 1.421)	0.6372	0.982 (0.737 to 1.310)	0.9032	1.141 (0.862 to 1.511)	0.3569	0.5504	1.003 (0.924 to 1.089)	0.9449
Men	Stomach cancer	TC	1	1.196 (0.931 to 1.538)	0.1614	1.008 (0.767 to 1.325)	0.9521	1.151 (0.876 to 1.512)	0.3123	0.920 (0.685 to 1.234)	0.5765	0.6482	0.973 (0.896 to 1.056)	0.51
Men	Stomach cancer	NONHDL	1	1.081 (0.838 to 1.394)	0.5483	1.170 (0.901 to 1.521)	0.2394	1.066 (0.812 to 1.399)	0.6463	0.856 (0.637 to 1.149)	0.2993	0.4527	0.974 (0.899 to 1.055)	0.5192
Men	Stomach cancer	AIP	1	1.144 (0.854 to 1.532)	0.3663	1.001 (0.743 to 1.348)	0.9973	1.097 (0.820 to 1.470)	0.5325	1.174 (0.875 to 1.574)	0.2842	0.4014	1.023 (0.932 to 1.123)	0.6311
Men	Stomach cancer	AC	1	1.186 (0.903 to 1.558)	0.2197	1.298 (0.993 to 1.698)	0.0567	1.315 (1.000 to 1.730)	0.05	1.111 (0.828 to 1.490)	0.4842	0.2831	1.006 (0.925 to 1.093)	0.8948
Men	Stomach cancer	CRI-I	1	1.186 (0.903 to 1.558)	0.2197	1.298 (0.993 to 1.698)	0.0567	1.315 (1.000 to 1.730)	0.05	1.111 (0.828 to 1.490)	0.4842	0.2831	1.006 (0.925 to 1.093)	0.8948
Men	Stomach cancer	CRI-II	1	1.101 (0.839 to 1.445)	0.4879	1.436 (1.108 to 1.861)	0.0063	1.255 (0.954 to 1.651)	0.1044	0.989 (0.734 to 1.332)	0.9406	0.5777	1.002 (0.922 to 1.090)	0.9538
Men	Stomach cancer	LCI	1	1.175 (0.898 to 1.538)	0.2398	1.133 (0.863 to 1.488)	0.369	1.142 (0.869 to 1.500)	0.3413	1.134 (0.854 to 1.506)	0.3836	0.4804	0.958 (0.884 to 1.038)	0.2979
Men	Liver cancer	THDL	1	1.144 (0.854 to 1.532)	0.3663	1.001 (0.743 to 1.348)	0.9973	1.097 (0.820 to 1.470)	0.5325	1.174 (0.875 to 1.574)	0.2842	0.4014	1.023 (0.943 to 1.110)	0.5813
Men	Liver cancer	HDL-C	1	0.917 (0.679 to 1.239)	0.5721	1.225 (0.915 to 1.639)	0.1729	0.918 (0.656 to 1.284)	0.6176	1.273 (0.908 to 1.784)	0.161	0.2328	1.061 (0.945 to 1.190)	0.3174
Men	Liver cancer	LDL-C	1	0.778 (0.588 to 1.028)	0.0773	0.833 (0.619 to 1.120)	0.2268	0.594 (0.422 to 0.835)	0.0027	0.531 (0.365 to 0.771)	<0.001	<0.001	0.807 (0.718 to 0.906)	<0.001
Men	Liver cancer	TG	1	1.189 (0.861 to 1.641)	0.2933	0.844 (0.601 to 1.186)	0.3289	0.979 (0.704 to 1.362)	0.8996	0.963 (0.692 to 1.341)	0.8243	0.4403	0.980 (0.880 to 1.091)	0.7121
Men	Liver cancer	TC	1	0.824 (0.623 to 1.090)	0.1742	0.807 (0.591 to 1.101)	0.1768	0.643 (0.458 to 0.902)	0.0106	0.646 (0.451 to 0.926)	0.0173	0.0044	0.833 (0.741 to 0.937)	0.0023
Men	Liver cancer	NONHDL	1	0.657 (0.494 to 0.874)	0.0039	0.705 (0.523 to 0.950)	0.0218	0.611 (0.437 to 0.854)	0.004	0.593 (0.419 to 0.840)	0.0033	0.0013	0.813 (0.723 to 0.915)	<0.001
Men	Liver cancer	AIP	1	0.961 (0.689 to 1.339)	0.812	0.802 (0.571 to 1.126)	0.2024	0.851 (0.609 to 1.189)	0.3441	0.881 (0.631 to 1.229)	0.4546	0.396	0.955 (0.855 to 1.067)	0.4166
Men	Liver cancer	AC	1	0.849 (0.646 to 1.116)	0.241	0.588 (0.431 to 0.801)	<0.001	0.591 (0.428 to 0.815)	0.0013	0.690 (0.499 to 0.953)	0.0245	0.0019	0.829 (0.736 to 0.934)	0.002
Men	Liver cancer	CRI-I	1	0.849 (0.646 to 1.116)	0.241	0.588 (0.431 to 0.801)	<0.001	0.591 (0.428 to 0.815)	0.0013	0.690 (0.499 to 0.953)	0.0245	0.0019	0.829 (0.736 to 0.934)	0.002
Men	Liver cancer	CRI-II	1	0.875 (0.668 to 1.147)	0.3344	0.599 (0.439 to 0.818)	0.0012	0.627 (0.455 to 0.864)	0.0043	0.690 (0.497 to 0.958)	0.0268	0.0023	0.813 (0.725 to 0.912)	<0.001
Men	Liver cancer	LCI	1	0.844 (0.634 to 1.124)	0.2457	0.762 (0.567 to 1.026)	0.0735	0.715 (0.523 to 0.977)	0.0351	0.679 (0.487 to 0.945)	0.0219	0.0113	0.894 (0.784 to 1.020)	0.0968
Men	Bladder cancer	THDL	1	0.961 (0.689 to 1.339)	0.812	0.802 (0.571 to 1.126)	0.2024	0.851 (0.609 to 1.189)	0.3441	0.881 (0.631 to 1.229)	0.4546	0.396	0.988 (0.889 to 1.098)	0.825
Men	Bladder cancer	HDL-C	1	1.019 (0.886 to 1.172)	0.7915	1.039 (0.900 to 1.199)	0.6048	0.968 (0.832 to 1.126)	0.6724	0.885 (0.753 to 1.040)	0.1389	0.1294	0.957 (0.910 to 1.007)	0.0918
Men	Bladder cancer	LDL-C	1	1.340 (1.174 to 1.530)	<0.001	1.188 (1.028 to 1.372)	0.0192	1.216 (1.051 to 1.408)	0.0085	1.219 (1.049 to 1.416)	0.0096	0.0416	1.057 (1.011 to 1.106)	0.0155
Men	Bladder cancer	TG	1	1.040 (0.894 to 1.211)	0.6116	1.126 (0.970 to 1.308)	0.12	1.140 (0.981 to 1.325)	0.0869	1.121 (0.961 to 1.308)	0.1476	0.0714	1.019 (0.973 to 1.067)	0.4257

Gender	Cancer type	Lipid index	1st quintile	2nd quintile		3rd quintile		4th quintile		5th quintile		p-value for trend	Per 1 SD increase	
				aHR (95% CI)	p-value	aHR (95% CI)	p-value	aHR (95% CI)	p-value	aHR (95% CI)	p-value		aHR (95% CI)	p-value
Men	Bladder cancer	TC	1	1.127 (0.985 to 1.290)	0.0813	1.195 (1.038 to 1.376)	0.013	1.077 (0.929 to 1.248)	0.3273	1.162 (1.002 to 1.348)	0.0467	0.0967	1.042 (0.995 to 1.090)	0.0774
Men	Bladder cancer	NONHDL	1	1.158 (1.012 to 1.325)	0.0323	1.158 (1.005 to 1.335)	0.0427	1.167 (1.010 to 1.349)	0.0359	1.188 (1.024 to 1.378)	0.0226	0.0243	1.055 (1.008 to 1.103)	0.0204
Men	Bladder cancer	AIP	1	1.068 (0.915 to 1.247)	0.4042	1.141 (0.979 to 1.330)	0.0922	1.268 (1.090 to 1.475)	0.0021	1.081 (0.921 to 1.268)	0.3409	0.072	1.043 (0.994 to 1.095)	0.0856
Men	Bladder cancer	AC	1	1.009 (0.875 to 1.163)	0.9004	1.218 (1.059 to 1.401)	0.0056	1.174 (1.015 to 1.359)	0.0304	1.184 (1.017 to 1.378)	0.0292	0.0035	1.066 (1.018 to 1.116)	0.0069
Men	Bladder cancer	CRI-I	1	1.009 (0.875 to 1.163)	0.9004	1.218 (1.059 to 1.401)	0.0056	1.174 (1.015 to 1.359)	0.0304	1.184 (1.017 to 1.378)	0.0292	0.0035	1.066 (1.018 to 1.116)	0.0069
Men	Bladder cancer	CRI-II	1	1.073 (0.933 to 1.233)	0.3251	1.178 (1.023 to 1.357)	0.0226	1.198 (1.035 to 1.386)	0.0153	1.197 (1.029 to 1.392)	0.0196	0.0047	1.075 (1.026 to 1.126)	0.0023
Men	Bladder cancer	LCI	1	1.133 (0.981 to 1.307)	0.0883	1.169 (1.013 to 1.350)	0.0332	1.286 (1.113 to 1.487)	<0.001	1.159 (0.994 to 1.351)	0.0594	0.0093	1.027 (0.982 to 1.074)	0.2451
Men	Bladder cancer	THDL	1	1.068 (0.915 to 1.247)	0.4042	1.141 (0.979 to 1.330)	0.0922	1.268 (1.090 to 1.475)	0.0021	1.081 (0.921 to 1.268)	0.3409	0.072	1.011 (0.968 to 1.057)	0.6175
Men	Prostate cancer	HDL-C	1	0.989 (0.922 to 1.061)	0.757	0.994 (0.926 to 1.067)	0.865	1.044 (0.972 to 1.121)	0.2382	1.020 (0.946 to 1.098)	0.6113	0.2735	1.025 (1.001 to 1.049)	0.0423
Men	Prostate cancer	LDL-C	1	1.172 (1.096 to 1.253)	<0.001	1.195 (1.116 to 1.281)	<0.001	1.208 (1.126 to 1.295)	<0.001	1.187 (1.105 to 1.275)	<0.001	<0.001	1.056 (1.034 to 1.080)	<0.001
Men	Prostate cancer	TG	1	1.005 (0.939 to 1.075)	0.8907	0.988 (0.922 to 1.058)	0.7256	1.027 (0.958 to 1.101)	0.4499	0.960 (0.892 to 1.032)	0.2677	0.5069	0.986 (0.963 to 1.010)	0.256
Men	Prostate cancer	TC	1	1.243 (1.162 to 1.329)	<0.001	1.146 (1.068 to 1.231)	<0.001	1.268 (1.182 to 1.360)	<0.001	1.215 (1.130 to 1.305)	<0.001	<0.001	1.056 (1.034 to 1.080)	<0.001
Men	Prostate cancer	NONHDL	1	1.140 (1.066 to 1.219)	<0.001	1.184 (1.106 to 1.268)	<0.001	1.196 (1.116 to 1.282)	<0.001	1.173 (1.092 to 1.260)	<0.001	<0.001	1.052 (1.029 to 1.075)	<0.001
Men	Prostate cancer	AIP	1	0.993 (0.928 to 1.063)	0.8395	0.993 (0.927 to 1.063)	0.8303	1.000 (0.932 to 1.072)	0.9932	0.937 (0.870 to 1.009)	0.0865	0.1656	0.980 (0.957 to 1.004)	0.1044
Men	Prostate cancer	AC	1	1.068 (1.000 to 1.140)	0.0492	1.051 (0.983 to 1.124)	0.1465	1.084 (1.012 to 1.162)	0.0212	1.094 (1.017 to 1.176)	0.0154	0.0158	1.028 (1.005 to 1.052)	0.0166
Men	Prostate cancer	CRI-I	1	1.068 (1.000 to 1.140)	0.0492	1.051 (0.983 to 1.124)	0.1465	1.084 (1.012 to 1.162)	0.0212	1.094 (1.017 to 1.176)	0.0154	0.0158	1.028 (1.005 to 1.052)	0.0166
Men	Prostate cancer	CRI-II	1	1.066 (0.999 to 1.138)	0.054	1.058 (0.989 to 1.132)	0.1017	1.081 (1.009 to 1.158)	0.0268	1.116 (1.039 to 1.199)	0.0027	0.0041	1.033 (1.010 to 1.057)	0.0046
Men	Prostate cancer	LCI	1	1.066 (0.997 to 1.138)	0.0597	1.053 (0.984 to 1.126)	0.1361	1.053 (0.983 to 1.128)	0.1415	1.063 (0.989 to 1.143)	0.0946	0.1606	1.014 (0.990 to 1.038)	0.2661
Men	Prostate cancer	THDL	1	0.993 (0.928 to 1.063)	0.8395	0.993 (0.927 to 1.063)	0.8303	1.000 (0.932 to 1.072)	0.9932	0.937 (0.870 to 1.009)	0.0865	0.1656	0.978 (0.954 to 1.003)	0.0817
Men	Thyroid cancer	HDL-C	1	0.582 (0.338 to 1.001)	0.0505	0.666 (0.388 to 1.144)	0.141	0.477 (0.261 to 0.871)	0.016	0.513 (0.270 to 0.974)	0.0412	0.0319	0.778 (0.604 to 1.001)	0.0506
Men	Thyroid cancer	LDL-C	1	1.182 (0.688 to 2.031)	0.5459	1.089 (0.615 to 1.926)	0.7706	1.093 (0.614 to 1.947)	0.7617	1.155 (0.652 to 2.045)	0.6216	0.7584	1.022 (0.833 to 1.253)	0.8351
Men	Thyroid cancer	TG	1	1.180 (0.608 to 2.290)	0.6257	1.427 (0.743 to 2.739)	0.2855	1.275 (0.651 to 2.496)	0.4783	1.739 (0.916 to 3.300)	0.0908	0.1	1.176 (1.008 to 1.373)	0.0399
Men	Thyroid cancer	TC	1	0.583 (0.312 to 1.088)	0.0901	0.887 (0.504 to 1.560)	0.6776	1.137 (0.666 to 1.943)	0.6381	0.899 (0.500 to 1.614)	0.7206	0.5728	1.012 (0.821 to 1.247)	0.9138
Men	Thyroid cancer	NONHDL	1	1.067 (0.595 to 1.912)	0.8283	1.321 (0.759 to 2.302)	0.3249	1.277 (0.729 to 2.238)	0.3925	1.285 (0.721 to 2.290)	0.3959	0.3055	1.069 (0.877 to 1.302)	0.5087
Men	Thyroid cancer	AIP	1	1.122 (0.568 to 2.218)	0.7399	1.605 (0.825 to 3.122)	0.1639	1.113 (0.553 to 2.241)	0.7633	1.801 (0.917 to 3.538)	0.0877	0.1116	1.277 (1.038 to 1.572)	0.0208

Gender	Cancer type	Lipid index	1st quintile	2nd quintile		3rd quintile		4th quintile		5th quintile		p-value for trend	Per 1 SD increase	
				aHR (95% CI)	p-value	aHR (95% CI)	p-value	aHR (95% CI)	p-value	aHR (95% CI)	p-value		aHR (95% CI)	p-value
Men	Thyroid cancer	AC	1	0.660 (0.344 to 1.265)	0.2105	0.892 (0.482 to 1.651)	0.7154	0.999 (0.553 to 1.802)	0.9963	1.682 (0.982 to 2.882)	0.0584	0.0275	1.202 (1.009 to 1.431)	0.0394
Men	Thyroid cancer	CRI-I	1	0.660 (0.344 to 1.265)	0.2105	0.892 (0.482 to 1.651)	0.7154	0.999 (0.553 to 1.802)	0.9963	1.682 (0.982 to 2.882)	0.0584	0.0275	1.202 (1.009 to 1.431)	0.0394
Men	Thyroid cancer	CRI-II	1	0.637 (0.333 to 1.216)	0.1711	0.952 (0.530 to 1.710)	0.8684	1.014 (0.573 to 1.794)	0.9631	1.453 (0.852 to 2.476)	0.1699	0.073	1.172 (0.980 to 1.400)	0.0813
Men	Thyroid cancer	LCI	1	1.191 (0.649 to 2.183)	0.5728	0.873 (0.454 to 1.678)	0.6836	1.029 (0.547 to 1.937)	0.9292	1.929 (1.101 to 3.381)	0.0216	0.0509	1.140 (1.006 to 1.292)	0.0394
Men	Thyroid cancer	THDL	1	1.122 (0.568 to 2.218)	0.7399	1.605 (0.825 to 3.122)	0.1639	1.113 (0.553 to 2.241)	0.7633	1.801 (0.917 to 3.538)	0.0877	0.1116	1.198 (1.050 to 1.367)	0.0074
Men	Pancreas Cancer	HDL-C	1	0.917 (0.710 to 1.184)	0.5074	1.039 (0.800 to 1.351)	0.7735	0.874 (0.662 to 1.155)	0.3438	1.021 (0.769 to 1.355)	0.8871	0.9821	1.014 (0.923 to 1.115)	0.7646
Men	Pancreas Cancer	LDL-C	1	0.963 (0.750 to 1.237)	0.7695	1.046 (0.807 to 1.357)	0.7328	1.087 (0.836 to 1.412)	0.5341	1.135 (0.872 to 1.477)	0.3453	0.243	1.039 (0.955 to 1.130)	0.3699
Men	Pancreas Cancer	TG	1	1.001 (0.772 to 1.297)	0.9945	0.876 (0.670 to 1.145)	0.3316	0.807 (0.614 to 1.061)	0.1252	1.061 (0.815 to 1.380)	0.6617	0.8441	0.999 (0.919 to 1.086)	0.9854
Men	Pancreas Cancer	TC	1	0.951 (0.739 to 1.222)	0.6922	0.906 (0.690 to 1.190)	0.4773	1.104 (0.851 to 1.432)	0.457	1.101 (0.844 to 1.437)	0.4771	0.3075	1.040 (0.955 to 1.132)	0.364
Men	Pancreas Cancer	NONHDL	1	0.814 (0.632 to 1.047)	0.1089	0.906 (0.699 to 1.173)	0.4534	1.120 (0.873 to 1.436)	0.3746	1.050 (0.809 to 1.361)	0.7152	0.2423	1.038 (0.955 to 1.128)	0.3824
Men	Pancreas Cancer	AIP	1	0.929 (0.713 to 1.211)	0.5878	0.960 (0.735 to 1.253)	0.7624	0.870 (0.663 to 1.140)	0.3121	0.935 (0.708 to 1.234)	0.6356	0.5478	1.001 (0.918 to 1.093)	0.9737
Men	Pancreas Cancer	AC	1	1.011 (0.791 to 1.293)	0.9295	1.003 (0.780 to 1.291)	0.9786	1.099 (0.853 to 1.416)	0.4641	0.990 (0.754 to 1.300)	0.943	0.8009	1.025 (0.939 to 1.118)	0.5813
Men	Pancreas Cancer	CRI-I	1	1.011 (0.791 to 1.293)	0.9295	1.003 (0.780 to 1.291)	0.9786	1.099 (0.853 to 1.416)	0.4641	0.990 (0.754 to 1.300)	0.943	0.8009	1.025 (0.939 to 1.118)	0.5813
Men	Pancreas Cancer	CRI-II	1	1.056 (0.828 to 1.347)	0.6616	1.044 (0.813 to 1.341)	0.7361	1.035 (0.799 to 1.340)	0.7952	1.084 (0.830 to 1.417)	0.5526	0.6395	1.028 (0.943 to 1.120)	0.5333
Men	Pancreas Cancer	LCI	1	1.081 (0.843 to 1.388)	0.5382	0.962 (0.742 to 1.248)	0.7723	1.028 (0.793 to 1.333)	0.8321	1.134 (0.872 to 1.476)	0.3473	0.5248	1.022 (0.939 to 1.112)	0.6184
Men	Pancreas Cancer	THDL	1	0.929 (0.713 to 1.211)	0.5878	0.960 (0.735 to 1.253)	0.7624	0.870 (0.663 to 1.140)	0.3121	0.935 (0.708 to 1.234)	0.6356	0.5478	0.993 (0.912 to 1.081)	0.8656

Note:

HDL-C: High Density Lipoprotein Cholesterol; LDL-C: Low density lipoprotein Cholesterol; TG: Triglycerides; TC: Total Cholesterol; NONHDL: Non-high-density lipoprotein Cholesterol; AIP: Atherogenic Index of plasma; AC: Atherogenic coefficient; CRI-I: Castelli risk index -I; CRI-II: Castelli risk index-II; LCI: Lipoprotein combination index; THDL: Triglyceride HDL-C ratio.

**: Overall cancer excluded participants of breast cancer and thyroid cancer.*

Model: Cox Proportional-Hazards Model adjusted for age, body mass index, Type 2 Diabetes mellitus, hypertension, smoking history, alcohol consumption.

Table S5. Results of the estimated association between serum lipids and overall cancer risk in KCPS-II Biobank

Gender	Cancer type	Lipid index	1st quintile	2nd quintile		3rd quintile		4th quintile		5th quintile		p-value for trend	Per 1 SD increase	
				aHR (95% CI)	p-value	aHR (95% CI)	p-value	aHR (95% CI)	p-value	aHR (95% CI)	p-value		aHR (95% CI)	p-value
Women	Overall cancer	HDL-C	1	0.937 (0.834 to 1.054)	0.279	0.964 (0.855 to 1.088)	0.5532	0.861 (0.761 to 0.975)	0.0181	0.908 (0.802 to 1.028)	0.1268	0.0505	0.974 (0.934 to 1.015)	0.2079
Women	Overall cancer	LDL-C	1	0.981 (0.861 to 1.118)	0.7736	1.016 (0.893 to 1.155)	0.8126	1.075 (0.947 to 1.221)	0.2644	0.994 (0.872 to 1.132)	0.923	0.6265	1.005 (0.966 to 1.046)	0.791
Women	Overall cancer	TG	1	1.037 (0.909 to 1.182)	0.5885	1.041 (0.914 to 1.186)	0.5439	1.134 (0.997 to 1.291)	0.0563	1.051 (0.919 to 1.201)	0.4696	0.2297	0.992 (0.956 to 1.030)	0.6877
Women	Overall cancer	TC	1	1.009 (0.885 to 1.150)	0.8938	1.097 (0.965 to 1.247)	0.1588	1.043 (0.917 to 1.187)	0.5211	0.999 (0.875 to 1.140)	0.9868	0.9138	0.990 (0.951 to 1.030)	0.6091
Women	Overall cancer	NONHDL	1	1.002 (0.877 to 1.145)	0.9708	1.110 (0.975 to 1.264)	0.116	1.060 (0.929 to 1.211)	0.3858	1.032 (0.901 to 1.182)	0.6471	0.5002	0.999 (0.960 to 1.039)	0.9485
Women	Overall cancer	AIP	1	0.994 (0.872 to 1.134)	0.9322	1.024 (0.899 to 1.167)	0.7237	1.100 (0.967 to 1.251)	0.1486	1.096 (0.961 to 1.251)	0.1708	0.0631	1.018 (0.977 to 1.061)	0.387
Women	Overall cancer	AC	1	1.033 (0.906 to 1.179)	0.6259	0.981 (0.858 to 1.121)	0.7735	1.139 (1.000 to 1.297)	0.0504	1.081 (0.945 to 1.237)	0.2543	0.0992	1.018 (0.974 to 1.063)	0.4328
Women	Overall cancer	CRI-I	1	1.032 (0.904 to 1.177)	0.6422	0.980 (0.857 to 1.120)	0.7646	1.138 (0.999 to 1.296)	0.0519	1.080 (0.945 to 1.236)	0.2592	0.1002	1.018 (0.974 to 1.063)	0.4328
Women	Overall cancer	CRI-II	1	1.007 (0.882 to 1.149)	0.9201	1.017 (0.891 to 1.161)	0.7989	1.105 (0.971 to 1.258)	0.131	1.101 (0.965 to 1.256)	0.1531	0.0599	1.023 (0.979 to 1.070)	0.3122
Women	Overall cancer	LCI	1	1.098 (0.959 to 1.257)	0.1766	1.137 (0.994 to 1.300)	0.0609	1.152 (1.007 to 1.319)	0.0397	1.116 (0.970 to 1.285)	0.1249	0.1208	0.986 (0.946 to 1.029)	0.5256
Women	Overall cancer	THDL	1	0.994 (0.872 to 1.134)	0.9322	1.024 (0.899 to 1.167)	0.7237	1.100 (0.967 to 1.251)	0.1486	1.096 (0.961 to 1.251)	0.1708	0.0631	1.003 (0.967 to 1.041)	0.8587
Women	Overall cancer*	HDL-C	1	0.924 (0.779 to 1.094)	0.3584	0.939 (0.787 to 1.120)	0.4837	0.881 (0.738 to 1.052)	0.1624	0.947 (0.793 to 1.131)	0.5504	0.4159	0.996 (0.938 to 1.057)	0.8871
Women	Overall cancer*	LDL-C	1	0.955 (0.783 to 1.167)	0.6544	1.042 (0.860 to 1.263)	0.6733	1.106 (0.917 to 1.334)	0.2914	1.037 (0.859 to 1.251)	0.7075	0.3373	1.023 (0.968 to 1.082)	0.4247
Women	Overall cancer*	TG	1	0.974 (0.796 to 1.190)	0.7937	1.135 (0.938 to 1.374)	0.1928	1.075 (0.887 to 1.303)	0.4609	1.022 (0.843 to 1.240)	0.8246	0.596	0.979 (0.931 to 1.029)	0.4044
Women	Overall cancer*	TC	1	0.968 (0.794 to 1.181)	0.7511	1.105 (0.913 to 1.338)	0.3062	1.038 (0.857 to 1.256)	0.7035	1.013 (0.837 to 1.226)	0.8948	0.738	1.006 (0.951 to 1.065)	0.822
Women	Overall cancer*	NONHDL	1	0.945 (0.773 to 1.156)	0.5819	1.069 (0.881 to 1.299)	0.4981	0.996 (0.819 to 1.211)	0.9679	1.041 (0.859 to 1.263)	0.6798	0.5427	1.008 (0.952 to 1.067)	0.788
Women	Overall cancer*	AIP	1	0.968 (0.794 to 1.180)	0.7449	1.049 (0.865 to 1.271)	0.6295	1.032 (0.853 to 1.249)	0.7471	1.066 (0.883 to 1.287)	0.5035	0.3781	1.009 (0.954 to 1.069)	0.7465
Women	Overall cancer*	AC	1	0.917 (0.754 to 1.116)	0.388	0.867 (0.712 to 1.056)	0.155	0.978 (0.810 to 1.180)	0.8137	0.988 (0.817 to 1.193)	0.8974	0.7515	1.027 (0.964 to 1.093)	0.4122
Women	Overall cancer*	CRI-I	1	0.925 (0.760 to 1.125)	0.4328	0.870 (0.714 to 1.060)	0.1673	0.982 (0.813 to 1.185)	0.8463	0.992 (0.821 to 1.198)	0.9307	0.7381	1.027 (0.964 to 1.093)	0.4122
Women	Overall cancer*	CRI-II	1	0.960 (0.788 to 1.170)	0.6885	0.920 (0.755 to 1.121)	0.4087	1.067 (0.883 to 1.289)	0.5035	1.070 (0.886 to 1.292)	0.4801	0.2265	1.041 (0.977 to 1.109)	0.2137
Women	Overall cancer*	LCI	1	1.082 (0.881 to 1.331)	0.4516	1.184 (0.970 to 1.446)	0.097	1.090 (0.892 to 1.333)	0.3991	1.124 (0.919 to 1.375)	0.2563	0.3625	1.007 (0.953 to 1.065)	0.7928
Women	Overall cancer*	THDL	1	0.968 (0.794 to 1.180)	0.7449	1.049 (0.865 to 1.271)	0.6295	1.032 (0.853 to 1.249)	0.7471	1.066 (0.883 to 1.287)	0.5035	0.3781	0.991 (0.943 to 1.042)	0.7253
Women	Lung cancer	HDL-C	1	0.877 (0.507 to 1.517)	0.6385	0.854 (0.478 to 1.526)	0.5938	0.732 (0.404 to 1.327)	0.3043	0.947 (0.533 to 1.682)	0.8526	0.6192	0.999 (0.815 to 1.226)	0.9937

Gender	Cancer type	Lipid index	1st quintile	2nd quintile		3rd quintile		4th quintile		5th quintile		p-value for trend	Per 1 SD increase	
				aHR (95% CI)	p-value	aHR (95% CI)	p-value	aHR (95% CI)	p-value	aHR (95% CI)	p-value		aHR (95% CI)	p-value
Women	Lung cancer	LDL-C	1	1.544 (0.650 to 3.669)	0.3253	2.283 (1.036 to 5.032)	0.0406	2.899 (1.355 to 6.202)	0.0061	1.603 (0.724 to 3.549)	0.245	0.1361	1.068 (0.912 to 1.250)	0.4165
Women	Lung cancer	TG	1	0.955 (0.463 to 1.972)	0.9016	1.537 (0.799 to 2.956)	0.1978	0.801 (0.391 to 1.641)	0.5444	0.999 (0.505 to 1.974)	0.9966	0.7188	0.985 (0.807 to 1.204)	0.8861
Women	Lung cancer	TC	1	1.712 (0.770 to 3.809)	0.1875	1.813 (0.834 to 3.938)	0.1330	1.989 (0.929 to 4.257)	0.0766	1.578 (0.733 to 3.396)	0.2435	0.3717	1.045 (0.884 to 1.234)	0.6088
Women	Lung cancer	NONHDL	1	0.815 (0.324 to 2.053)	0.6645	2.472 (1.181 to 5.172)	0.0163	1.746 (0.807 to 3.778)	0.1569	1.484 (0.687 to 3.203)	0.3152	0.2038	1.044 (0.885 to 1.231)	0.6097
Women	Lung cancer	AIP	1	1.046 (0.535 to 2.047)	0.8946	0.946 (0.485 to 1.843)	0.8699	0.759 (0.383 to 1.506)	0.4306	1.022 (0.534 to 1.955)	0.9478	0.8448	0.967 (0.790 to 1.185)	0.7497
Women	Lung cancer	AC	1	0.823 (0.391 to 1.732)	0.6071	0.895 (0.438 to 1.829)	0.7608	1.275 (0.668 to 2.433)	0.4611	1.109 (0.573 to 2.145)	0.7587	0.3796	1.049 (0.875 to 1.257)	0.6065
Women	Lung cancer	CRI-I	1	0.821 (0.390 to 1.729)	0.6033	0.894 (0.437 to 1.827)	0.7585	1.274 (0.667 to 2.430)	0.4632	1.108 (0.573 to 2.142)	0.7613	0.3802	1.049 (0.875 to 1.257)	0.6065
Women	Lung cancer	CRI-II	1	0.764 (0.355 to 1.648)	0.4932	0.981 (0.487 to 1.978)	0.9574	1.183 (0.608 to 2.302)	0.6215	1.323 (0.701 to 2.498)	0.3877	0.1498	1.074 (0.900 to 1.280)	0.4296
Women	Lung cancer	LCI	1	1.881 (0.779 to 4.541)	0.1601	2.526 (1.099 to 5.805)	0.0290	2.033 (0.879 to 4.704)	0.0972	1.719 (0.735 to 4.021)	0.2115	0.5392	1.095 (0.944 to 1.270)	0.2289
Women	Lung cancer	THDL	1	1.046 (0.535 to 2.047)	0.8946	0.946 (0.485 to 1.843)	0.8699	0.759 (0.383 to 1.506)	0.4306	1.022 (0.534 to 1.955)	0.9478	0.8448	1.024 (0.864 to 1.213)	0.7867
Women	Colon cancer	HDL-C	1	1.542 (0.792 to 3.003)	0.2026	1.213 (0.581 to 2.534)	0.6072	1.798 (0.955 to 3.383)	0.0691	0.818 (0.359 to 1.865)	0.6333	0.9438	0.975 (0.799 to 1.190)	0.8056
Women	Colon cancer	LDL-C	1	1.348 (0.582 to 3.122)	0.4863	1.007 (0.414 to 2.451)	0.9874	1.836 (0.835 to 4.041)	0.1309	1.259 (0.567 to 2.796)	0.5707	0.4110	1.114 (0.891 to 1.393)	0.3434
Women	Colon cancer	TG	1	0.780 (0.334 to 1.819)	0.5649	0.840 (0.371 to 1.901)	0.6762	0.939 (0.425 to 2.075)	0.8764	1.264 (0.608 to 2.627)	0.5310	0.3703	1.015 (0.888 to 1.159)	0.8318
Women	Colon cancer	TC	1	2.075 (0.799 to 5.390)	0.1337	2.132 (0.831 to 5.469)	0.1151	2.676 (1.080 to 6.631)	0.0335	1.535 (0.592 to 3.979)	0.3775	0.4624	1.095 (0.918 to 1.305)	0.3132
Women	Colon cancer	NONHDL	1	1.221 (0.467 to 3.195)	0.6837	1.768 (0.711 to 4.397)	0.2199	1.941 (0.801 to 4.700)	0.1418	1.689 (0.701 to 4.067)	0.2428	0.1475	1.101 (0.930 to 1.303)	0.2659
Women	Colon cancer	AIP	1	0.630 (0.244 to 1.623)	0.3387	1.423 (0.669 to 3.027)	0.3591	1.173 (0.536 to 2.567)	0.6889	1.180 (0.570 to 2.443)	0.656	0.3618	1.071 (0.883 to 1.298)	0.4881
Women	Colon cancer	AC	1	0.926 (0.384 to 2.234)	0.8638	1.051 (0.449 to 2.459)	0.9088	1.302 (0.589 to 2.874)	0.5142	1.361 (0.628 to 2.953)	0.4351	0.2625	1.063 (0.883 to 1.279)	0.5187
Women	Colon cancer	CRI-I	1	0.925 (0.383 to 2.230)	0.8616	1.050 (0.449 to 2.457)	0.9100	1.301 (0.589 to 2.872)	0.5152	1.360 (0.627 to 2.950)	0.4359	0.2627	1.063 (0.883 to 1.279)	0.5187
Women	Colon cancer	CRI-II	1	1.227 (0.516 to 2.919)	0.6435	1.276 (0.538 to 3.024)	0.5799	1.359 (0.593 to 3.114)	0.4679	1.512 (0.674 to 3.392)	0.3153	0.3028	1.080 (0.884 to 1.320)	0.4518
Women	Colon cancer	LCI	1	0.586 (0.207 to 1.658)	0.314	1.449 (0.634 to 3.315)	0.3794	1.376 (0.591 to 3.202)	0.4587	1.378 (0.615 to 3.089)	0.4365	0.1496	1.012 (0.896 to 1.143)	0.8455
Women	Colon cancer	THDL	1	0.630 (0.244 to 1.623)	0.3387	1.423 (0.669 to 3.027)	0.3591	1.173 (0.536 to 2.567)	0.6889	1.180 (0.570 to 2.443)	0.656	0.3618	1.004 (0.867 to 1.161)	0.9623
Women	Rectal cancer	HDL-C	1	1.010 (0.483 to 2.113)	0.9785	1.118 (0.530 to 2.357)	0.7693	0.323 (0.103 to 1.011)	0.0523	0.995 (0.471 to 2.103)	0.9899	0.4287	0.934 (0.730 to 1.194)	0.5864
Women	Rectal cancer	LDL-C	1	2.001 (0.763 to 5.248)	0.1583	1.262 (0.446 to 3.572)	0.6611	1.610 (0.611 to 4.244)	0.3355	1.826 (0.701 to 4.758)	0.2176	0.4111	1.124 (0.844 to 1.497)	0.4255
Women	Rectal cancer	TG	1	0.421 (0.174 to 1.016)	0.0542	0.392 (0.163 to 0.940)	0.0358	0.445 (0.196 to 1.010)	0.0528	0.838 (0.414 to 1.697)	0.6239	0.7668	1.036 (0.866 to 1.239)	0.6968
Women	Rectal cancer	TC	1	0.718 (0.284 to 1.815)	0.4833	0.911 (0.380 to 2.183)	0.8348	0.935 (0.409 to 2.138)	0.8735	0.972 (0.413 to 2.287)	0.9474	0.8207	1.075 (0.845 to 1.367)	0.555

Gender	Cancer type	Lipid index	1st quintile	2nd quintile		3rd quintile		4th quintile		5th quintile		p-value for trend	Per 1 SD increase	
				aHR (95% CI)	p-value	aHR (95% CI)	p-value	aHR (95% CI)	p-value	aHR (95% CI)	p-value		aHR (95% CI)	p-value
Women	Rectal cancer	NONHDL	1	0.884 (0.365 to 2.138)	0.7838	0.571 (0.215 to 1.518)	0.2615	0.925 (0.401 to 2.133)	0.8551	1.072 (0.462 to 2.488)	0.8718	0.7399	1.096 (0.870 to 1.383)	0.4363
Women	Rectal cancer	AIP	1	0.619 (0.252 to 1.521)	0.2962	0.518 (0.203 to 1.322)	0.1689	0.773 (0.354 to 1.689)	0.5182	1.048 (0.481 to 2.282)	0.9068	0.7361	1.018 (0.773 to 1.341)	0.9
Women	Rectal cancer	AC	1	1.041 (0.444 to 2.442)	0.9268	0.604 (0.235 to 1.553)	0.2952	1.001 (0.441 to 2.277)	0.9972	1.113 (0.499 to 2.485)	0.7934	0.7814	1.126 (0.858 to 1.480)	0.3922
Women	Rectal cancer	CRI-I	1	1.039 (0.443 to 2.438)	0.9295	0.603 (0.235 to 1.551)	0.2945	1.001 (0.440 to 2.275)	0.9987	1.112 (0.498 to 2.484)	0.7948	0.782	1.126 (0.858 to 1.480)	0.3922
Women	Rectal cancer	CRI-II	1	0.947 (0.394 to 2.276)	0.9026	0.969 (0.417 to 2.250)	0.9418	0.610 (0.239 to 1.559)	0.3018	1.292 (0.587 to 2.845)	0.5244	0.7248	1.152 (0.852 to 1.557)	0.3578
Women	Rectal cancer	LCI	1	0.426 (0.163 to 1.110)	0.0808	0.510 (0.208 to 1.251)	0.1411	0.565 (0.249 to 1.279)	0.1708	1.026 (0.481 to 2.191)	0.9464	0.6584	1.041 (0.889 to 1.219)	0.6171
Women	Rectal cancer	THDL	1	0.619 (0.252 to 1.521)	0.2962	0.518 (0.203 to 1.322)	0.1689	0.773 (0.354 to 1.689)	0.5182	1.048 (0.481 to 2.282)	0.9068	0.7361	1.034 (0.873 to 1.224)	0.701
Women	Stomach cancer	HDL-C	1	0.754 (0.468 to 1.215)	0.2457	0.704 (0.422 to 1.173)	0.1781	0.569 (0.326 to 0.996)	0.0483	0.772 (0.464 to 1.286)	0.3208	0.1723	0.883 (0.739 to 1.055)	0.1716
Women	Stomach cancer	LDL-C	1	0.849 (0.470 to 1.534)	0.5878	0.973 (0.556 to 1.704)	0.9248	0.867 (0.495 to 1.518)	0.6174	1.226 (0.726 to 2.069)	0.4462	0.3723	1.046 (0.895 to 1.221)	0.5734
Women	Stomach cancer	TG	1	1.474 (0.804 to 2.703)	0.2095	1.325 (0.726 to 2.416)	0.3593	1.446 (0.800 to 2.614)	0.2217	1.359 (0.759 to 2.435)	0.3016	0.4406	1.027 (0.929 to 1.136)	0.5976
Women	Stomach cancer	TC	1	0.736 (0.407 to 1.332)	0.3113	1.142 (0.676 to 1.927)	0.6201	0.761 (0.436 to 1.328)	0.3369	0.968 (0.576 to 1.629)	0.9037	0.991	0.974 (0.830 to 1.142)	0.7429
Women	Stomach cancer	NONHDL	1	1.285 (0.707 to 2.335)	0.4101	1.166 (0.639 to 2.129)	0.6167	1.051 (0.573 to 1.929)	0.8723	1.310 (0.738 to 2.324)	0.356	0.6013	1.014 (0.869 to 1.182)	0.8615
Women	Stomach cancer	AIP	1	2.024 (1.092 to 3.751)	0.0252	1.175 (0.601 to 2.297)	0.6366	1.854 (0.995 to 3.457)	0.052	1.693 (0.916 to 3.126)	0.0927	0.2188	1.116 (0.955 to 1.304)	0.1681
Women	Stomach cancer	AC	1	0.810 (0.445 to 1.475)	0.4912	0.817 (0.452 to 1.477)	0.5031	1.053 (0.599 to 1.850)	0.8582	1.111 (0.635 to 1.943)	0.7125	0.4203	1.066 (0.911 to 1.248)	0.4264
Women	Stomach cancer	CRI-I	1	0.809 (0.445 to 1.473)	0.4883	0.816 (0.451 to 1.476)	0.5016	1.052 (0.598 to 1.849)	0.8604	1.110 (0.635 to 1.941)	0.7146	0.4209	1.066 (0.911 to 1.248)	0.4264
Women	Stomach cancer	CRI-II	1	1.038 (0.566 to 1.903)	0.9045	0.960 (0.519 to 1.777)	0.8972	1.200 (0.668 to 2.157)	0.5424	1.437 (0.806 to 2.561)	0.2191	0.1545	1.090 (0.933 to 1.273)	0.2759
Women	Stomach cancer	LCI	1	0.977 (0.515 to 1.856)	0.9445	1.245 (0.681 to 2.273)	0.4765	1.059 (0.579 to 1.937)	0.8524	1.422 (0.783 to 2.584)	0.2472	0.2088	1.017 (0.915 to 1.130)	0.7535
Women	Stomach cancer	THDL	1	2.024 (1.092 to 3.751)	0.0252	1.175 (0.601 to 2.297)	0.6366	1.854 (0.995 to 3.457)	0.052	1.693 (0.916 to 3.126)	0.0927	0.2188	1.044 (0.949 to 1.147)	0.3768
Women	Liver cancer	HDL-C	1	0.929 (0.332 to 2.599)	0.8879	1.150 (0.410 to 3.228)	0.791	0.369 (0.081 to 1.689)	0.199	1.420 (0.490 to 4.121)	0.5185	0.9367	1.156 (0.739 to 1.809)	0.5247
Women	Liver cancer	LDL-C	1	0.410 (0.101 to 1.663)	0.212	0.558 (0.166 to 1.878)	0.3462	0.597 (0.187 to 1.903)	0.3832	0.580 (0.184 to 1.833)	0.3537	0.6404	1.002 (0.654 to 1.536)	0.9917
Women	Liver cancer	TG	1	0.850 (0.170 to 4.247)	0.8427	1.313 (0.320 to 5.391)	0.7051	1.428 (0.359 to 5.676)	0.6131	1.042 (0.265 to 4.102)	0.9531	0.8315	0.854 (0.655 to 1.113)	0.2423
Women	Liver cancer	TC	1	1.050 (0.280 to 3.943)	0.9425	1.346 (0.397 to 4.566)	0.6337	0.364 (0.079 to 1.668)	0.1933	0.941 (0.275 to 3.219)	0.9225	0.5755	1.046 (0.684 to 1.599)	0.8355
Women	Liver cancer	NONHDL	1	0.291 (0.055 to 1.528)	0.1446	0.739 (0.223 to 2.445)	0.6202	0.660 (0.195 to 2.236)	0.5048	0.542 (0.153 to 1.919)	0.3423	0.6945	0.995 (0.651 to 1.523)	0.9832
Women	Liver cancer	AIP	1	0.365 (0.069 to 1.919)	0.234	0.433 (0.107 to 1.755)	0.241	1.094 (0.360 to 3.319)	0.8744	0.658 (0.204 to 2.128)	0.4849	0.9391	0.922 (0.665 to 1.278)	0.6259
Women	Liver cancer	AC	1	0.136 (0.016 to 1.139)	0.0658	0.526 (0.152 to 1.816)	0.3096	0.690 (0.228 to 2.091)	0.5117	0.482 (0.146 to 1.590)	0.2305	0.7207	1.008 (0.630 to 1.615)	0.9721

Gender	Cancer type	Lipid index	1st quintile	2nd quintile		3rd quintile		4th quintile		5th quintile		p-value for trend	Per 1 SD increase	
				aHR (95% CI)	p-value	aHR (95% CI)	p-value	aHR (95% CI)	p-value	aHR (95% CI)	p-value		aHR (95% CI)	p-value
Women	Liver cancer	CRI-I	1	0.136 (0.016 to 1.138)	0.0657	0.526 (0.152 to 1.815)	0.3093	0.689 (0.227 to 2.090)	0.5111	0.481 (0.146 to 1.589)	0.2301	0.7205	1.008 (0.630 to 1.615)	0.9721
Women	Liver cancer	CRI-II	1	0.967 (0.299 to 3.131)	0.9557	0.374 (0.086 to 1.632)	0.1907	0.918 (0.295 to 2.853)	0.882	0.421 (0.117 to 1.520)	0.1866	0.2374	1.029 (0.620 to 1.709)	0.9114
Women	Liver cancer	LCI	1	0.569 (0.126 to 2.573)	0.4644	0.539 (0.129 to 2.257)	0.3978	0.915 (0.253 to 3.307)	0.8918	0.564 (0.145 to 2.201)	0.4101	0.6946	0.936 (0.657 to 1.334)	0.7146
Women	Liver cancer	THDL	1	0.365 (0.069 to 1.919)	0.234	0.433 (0.107 to 1.755)	0.241	1.094 (0.360 to 3.319)	0.8744	0.658 (0.204 to 2.128)	0.4849	0.9391	0.869 (0.655 to 1.154)	0.3328
Women	Breast cancer	HDL-C	1	0.981 (0.767 to 1.255)	0.8782	1.052 (0.821 to 1.350)	0.6876	0.785 (0.601 to 1.024)	0.0744	1.082 (0.840 to 1.392)	0.5424	0.8882	1.004 (0.924 to 1.092)	0.9173
Women	Breast cancer	LDL-C	1	0.970 (0.742 to 1.267)	0.8228	0.965 (0.739 to 1.260)	0.7926	1.277 (0.991 to 1.646)	0.0586	0.977 (0.745 to 1.281)	0.8651	0.422	1.030 (0.948 to 1.119)	0.4856
Women	Breast cancer	TG	1	1.231 (0.947 to 1.600)	0.1212	1.032 (0.784 to 1.358)	0.8238	1.252 (0.956 to 1.638)	0.1022	1.217 (0.917 to 1.615)	0.174	0.2207	1.009 (0.931 to 1.093)	0.8266
Women	Breast cancer	TC	1	1.035 (0.789 to 1.358)	0.8058	1.205 (0.926 to 1.568)	0.166	1.096 (0.838 to 1.435)	0.5028	1.169 (0.888 to 1.539)	0.2654	0.2485	1.028 (0.949 to 1.114)	0.5014
Women	Breast cancer	NONHDL	1	0.964 (0.731 to 1.272)	0.7976	1.194 (0.917 to 1.555)	0.188	1.219 (0.933 to 1.594)	0.1469	1.065 (0.802 to 1.414)	0.6631	0.2682	1.026 (0.946 to 1.113)	0.5364
Women	Breast cancer	AIP	1	1.053 (0.810 to 1.370)	0.6976	0.992 (0.758 to 1.297)	0.952	1.183 (0.911 to 1.537)	0.2067	1.107 (0.837 to 1.463)	0.4776	0.3167	1.017 (0.929 to 1.112)	0.7188
Women	Breast cancer	AC	1	1.219 (0.937 to 1.587)	0.1402	0.975 (0.738 to 1.288)	0.8593	1.277 (0.977 to 1.670)	0.0735	1.122 (0.846 to 1.489)	0.4249	0.4011	1.024 (0.933 to 1.123)	0.6244
Women	Breast cancer	CRI-I	1	1.218 (0.936 to 1.585)	0.1425	0.975 (0.738 to 1.287)	0.8561	1.277 (0.976 to 1.669)	0.0742	1.121 (0.845 to 1.488)	0.4273	0.4022	1.024 (0.933 to 1.123)	0.6244
Women	Breast cancer	CRI-II	1	0.975 (0.748 to 1.270)	0.8495	0.977 (0.750 to 1.273)	0.8645	1.034 (0.793 to 1.349)	0.8034	1.091 (0.834 to 1.428)	0.5247	0.4468	1.026 (0.934 to 1.127)	0.595
Women	Breast cancer	LCI	1	1.089 (0.829 to 1.430)	0.5393	1.111 (0.845 to 1.460)	0.4514	1.212 (0.920 to 1.596)	0.1708	1.192 (0.889 to 1.598)	0.2413	0.1791	0.993 (0.917 to 1.077)	0.8736
Women	Breast cancer	THDL	1	1.053 (0.810 to 1.370)	0.6976	0.992 (0.758 to 1.297)	0.952	1.183 (0.911 to 1.537)	0.2067	1.107 (0.837 to 1.463)	0.4776	0.3167	1.016 (0.935 to 1.105)	0.7017
Women	Cervix uteri cancer	HDL	1	0.970 (0.348 to 2.703)	0.954	0.878 (0.298 to 2.583)	0.8126	0.879 (0.298 to 2.594)	0.8153	1.337 (0.498 to 3.588)	0.5642	0.6589	0.970 (0.670 to 1.404)	0.8719
Women	Cervix uteri cancer	LDL	1	0.397 (0.076 to 2.066)	0.2722	1.149 (0.352 to 3.744)	0.8182	1.756 (0.572 to 5.397)	0.3254	2.627 (0.873 to 7.911)	0.0859	0.0198	1.524 (1.103 to 2.105)	0.0105
Women	Cervix uteri cancer	TG	1	0.499 (0.124 to 2.014)	0.3286	0.850 (0.257 to 2.816)	0.7907	1.789 (0.645 to 4.958)	0.2637	1.656 (0.564 to 4.863)	0.3587	0.0971	1.101 (1.003 to 1.209)	0.0424
Women	Cervix uteri cancer	TC	1	0.988 (0.252 to 3.873)	0.9861	0.495 (0.090 to 2.709)	0.4174	2.185 (0.660 to 7.230)	0.2004	4.009 (1.244 to 12.927)	0.0201	0.0075	1.190 (1.062 to 1.334)	0.0027
Women	Cervix uteri cancer	NONHDL	1	1.295 (0.293 to 5.723)	0.7331	1.298 (0.295 to 5.717)	0.73	3.610 (1.006 to 12.956)	0.049	4.410 (1.199 to 16.220)	0.0255	0.0058	1.197 (1.067 to 1.344)	0.0023
Women	Cervix uteri cancer	AIP	1	0.288 (0.060 to 1.369)	0.1175	1.434 (0.540 to 3.803)	0.4692	0.936 (0.318 to 2.755)	0.9038	1.202 (0.426 to 3.391)	0.7283	0.4174	1.279 (0.923 to 1.774)	0.1398
Women	Cervix uteri cancer	AC	1	0.732 (0.163 to 3.284)	0.6835	2.454 (0.748 to 8.045)	0.1385	1.426 (0.388 to 5.244)	0.5932	2.977 (0.905 to 9.790)	0.0724	0.04	1.261 (1.076 to 1.478)	0.0041
Women	Cervix uteri cancer	CRI1	1	0.731 (0.163 to 3.281)	0.6825	2.452 (0.748 to 8.040)	0.1387	1.425 (0.388 to 5.241)	0.5938	2.976 (0.905 to 9.787)	0.0726	0.0401	1.261 (1.076 to 1.478)	0.0041
Women	Cervix uteri cancer	CRI2	1	1.454 (0.411 to 5.144)	0.5619	1.221 (0.318 to 4.693)	0.7716	1.907 (0.562 to 6.464)	0.3001	3.113 (0.957 to 10.119)	0.0591	0.0553	1.302 (1.094 to 1.549)	0.0029
Women	Cervix uteri cancer	LCI	1	1.013 (0.203 to 5.056)	0.9873	2.015 (0.512 to 7.930)	0.3164	4.017 (1.107 to 14.576)	0.0345	4.057 (1.067 to 15.428)	0.0399	0.0036	1.126 (1.041 to 1.217)	0.0029

Gender	Cancer type	Lipid index	1st quintile	2nd quintile		3rd quintile		4th quintile		5th quintile		p-value for trend	Per 1 SD increase	
				aHR (95% CI)	p-value	aHR (95% CI)	p-value	aHR (95% CI)	p-value	aHR (95% CI)	p-value		aHR (95% CI)	p-value
Women	Cervix uteri cancer	THDL	1	0.288 (0.060 to 1.369)	0.1175	1.434 (0.540 to 3.803)	0.4692	0.936 (0.318 to 2.755)	0.9038	1.202 (0.426 to 3.391)	0.7283	0.4174	1.112 (1.011 to 1.224)	0.0288
Women	Thyroid cancer	HDL-C	1	0.917 (0.743 to 1.132)	0.4219	0.935 (0.754 to 1.160)	0.5429	0.870 (0.697 to 1.085)	0.2168	0.698 (0.551 to 0.884)	0.0029	0.0045	0.900 (0.833 to 0.972)	0.0076
Women	Thyroid cancer	LDL-C	1	0.998 (0.799 to 1.246)	0.9846	0.999 (0.799 to 1.248)	0.9898	0.890 (0.705 to 1.124)	0.3295	0.935 (0.737 to 1.187)	0.5821	0.3738	0.962 (0.893 to 1.037)	0.3175
Women	Thyroid cancer	TG	1	1.010 (0.805 to 1.266)	0.9326	0.927 (0.735 to 1.170)	0.5251	1.143 (0.911 to 1.432)	0.2479	1.013 (0.796 to 1.291)	0.9146	0.5733	1.014 (0.942 to 1.091)	0.7138
Women	Thyroid cancer	TC	1	1.011 (0.810 to 1.262)	0.9225	0.987 (0.788 to 1.236)	0.9085	1.007 (0.805 to 1.260)	0.9487	0.849 (0.665 to 1.084)	0.1897	0.2411	0.933 (0.865 to 1.007)	0.0743
Women	Thyroid cancer	NONHDL	1	1.101 (0.877 to 1.383)	0.4085	1.105 (0.878 to 1.390)	0.3942	1.048 (0.826 to 1.329)	0.7002	0.981 (0.762 to 1.264)	0.8816	0.7396	0.968 (0.898 to 1.043)	0.3917
Women	Thyroid cancer	AIP	1	1.006 (0.798 to 1.269)	0.9585	1.031 (0.816 to 1.302)	0.7989	1.186 (0.943 to 1.494)	0.1454	1.169 (0.917 to 1.490)	0.2068	0.089	1.047 (0.970 to 1.131)	0.2369
Women	Thyroid cancer	AC	1	1.122 (0.883 to 1.424)	0.3465	1.223 (0.964 to 1.553)	0.0972	1.356 (1.068 to 1.720)	0.0123	1.249 (0.969 to 1.609)	0.0857	0.0284	1.008 (0.935 to 1.087)	0.8323
Women	Thyroid cancer	CRI-I	1	1.104 (0.870 to 1.402)	0.4148	1.213 (0.957 to 1.539)	0.111	1.344 (1.060 to 1.705)	0.0147	1.238 (0.962 to 1.595)	0.0976	0.0307	1.008 (0.935 to 1.087)	0.8323
Women	Thyroid cancer	CRI-II	1	1.147 (0.906 to 1.450)	0.2543	1.219 (0.964 to 1.543)	0.0988	1.254 (0.990 to 1.589)	0.0605	1.196 (0.931 to 1.536)	0.161	0.1253	1.002 (0.925 to 1.086)	0.9591
Women	Thyroid cancer	LCI	1	1.146 (0.908 to 1.447)	0.252	1.119 (0.882 to 1.419)	0.3534	1.228 (0.966 to 1.562)	0.0937	1.083 (0.834 to 1.405)	0.5515	0.4449	0.939 (0.867 to 1.017)	0.1205
Women	Thyroid cancer	THDL	1	1.006 (0.798 to 1.269)	0.9585	1.031 (0.816 to 1.302)	0.7989	1.186 (0.943 to 1.494)	0.1454	1.169 (0.917 to 1.490)	0.2068	0.089	1.025 (0.961 to 1.094)	0.4459
Women	Pancreas cancer	HDL-C	1	0.769 (0.276 to 2.142)	0.6147	0.331 (0.072 to 1.526)	0.1564	1.243 (0.472 to 3.273)	0.6592	0.674 (0.220 to 2.066)	0.4894	0.7843	1.022 (0.688 to 1.517)	0.9155
Women	Pancreas cancer	LDL-C	1	0.692 (0.208 to 2.308)	0.5494	0.435 (0.119 to 1.583)	0.2064	0.486 (0.157 to 1.511)	0.2125	0.605 (0.209 to 1.747)	0.3527	0.4432	0.948 (0.637 to 1.410)	0.7911
Women	Pancreas cancer	TG	1	1.223 (0.198 to 7.554)	0.8285	2.422 (0.486 to 12.076)	0.2806	1.102 (0.188 to 6.443)	0.9143	2.043 (0.389 to 10.721)	0.3982	0.4527	1.067 (0.934 to 1.219)	0.3375
Women	Pancreas cancer	TC	1	0.378 (0.092 to 1.549)	0.1762	0.438 (0.121 to 1.588)	0.2091	0.381 (0.116 to 1.251)	0.1115	0.747 (0.263 to 2.124)	0.5849	0.9859	1.092 (0.715 to 1.667)	0.6829
Women	Pancreas cancer	NONHDL	1	1.079 (0.294 to 3.955)	0.9089	0.437 (0.097 to 1.958)	0.2793	0.508 (0.137 to 1.887)	0.3117	0.888 (0.265 to 2.972)	0.8466	0.804	1.083 (0.708 to 1.656)	0.7131
Women	Pancreas cancer	AIP	1	3.558 (0.404 to 31.334)	0.2528	5.495 (0.705 to 42.805)	0.1038	2.679 (0.334 to 21.514)	0.3539	3.987 (0.547 to 29.034)	0.1722	0.4029	1.134 (0.818 to 1.572)	0.4495
Women	Pancreas cancer	AC	1	0.778 (0.220 to 2.754)	0.6968	0.226 (0.045 to 1.131)	0.0704	0.577 (0.186 to 1.786)	0.3399	0.679 (0.214 to 2.148)	0.5096	0.7259	1.092 (0.716 to 1.665)	0.6826
Women	Pancreas cancer	CRI-I	1	0.776 (0.219 to 2.747)	0.6943	0.226 (0.045 to 1.130)	0.0701	0.576 (0.186 to 1.783)	0.3388	0.678 (0.214 to 2.144)	0.5081	0.7255	1.092 (0.716 to 1.665)	0.6826
Women	Pancreas cancer	CRI-II	1	0.648 (0.200 to 2.107)	0.4713	0.000 (0.000 to 0.000)	<0.001	0.720 (0.267 to 1.945)	0.5175	0.528 (0.180 to 1.552)	0.246	0.5958	0.981 (0.625 to 1.539)	0.9332
Women	Pancreas cancer	LCI	1	1.029 (0.897 to 1.122)	0.9667	1.058 (0.949 to 1.183)	0.3052	1.033 (0.936 to 1.145)	0.5591	1.061 (0.937 to 1.178)	0.3912	0.3191	1.100 (0.935 to 1.294)	0.2505
Women	Pancreas cancer	THDL	1	3.558 (0.404 to 31.334)	0.2528	5.495 (0.705 to 42.805)	0.1038	2.679 (0.334 to 21.514)	0.3539	3.987 (0.547 to 29.034)	0.1722	0.4029	1.074 (0.897 to 1.287)	0.437
Women	Ovarian cancer	HDL-C	1	0.994 (0.425 to 2.322)	0.9888	0.908 (0.363 to 2.267)	0.8359	1.638 (0.744 to 3.607)	0.2207	1.249 (0.512 to 3.045)	0.6249	0.3247	1.047 (0.803 to 1.365)	0.7352
Women	Ovarian cancer	LDL-C	1	0.587 (0.226 to 1.525)	0.274	0.754 (0.313 to 1.813)	0.5277	0.637 (0.259 to 1.568)	0.3261	0.990 (0.454 to 2.161)	0.9808	0.8742	1.130 (0.853 to 1.497)	0.3929

Gender	Cancer type	Lipid index	1st quintile	2nd quintile		3rd quintile		4th quintile		5th quintile		p-value for trend	Per 1 SD increase	
				aHR (95% CI)	p-value	aHR (95% CI)	p-value	aHR (95% CI)	p-value	aHR (95% CI)	p-value		aHR (95% CI)	p-value
Women	Ovarian cancer	TG	1	0.289 (0.106 to 0.784)	0.0148	0.493 (0.223 to 1.091)	0.081	0.427 (0.187 to 0.975)	0.0433	0.559 (0.251 to 1.248)	0.1559	0.3049	0.993 (0.729 to 1.352)	0.9624
Women	Ovarian cancer	TC	1	0.335 (0.106 to 1.058)	0.0624	0.795 (0.337 to 1.876)	0.6011	1.005 (0.445 to 2.269)	0.9902	0.891 (0.410 to 1.938)	0.7709	0.5288	1.107 (0.907 to 1.353)	0.3176
Women	Ovarian cancer	NONHDL	1	0.710 (0.278 to 1.811)	0.4733	0.888 (0.372 to 2.123)	0.7900	0.592 (0.227 to 1.543)	0.2833	1.000 (0.443 to 2.256)	0.9998	0.9799	1.094 (0.862 to 1.388)	0.4585
Women	Ovarian cancer	AIP	1	0.606 (0.264 to 1.392)	0.2376	0.249 (0.082 to 0.753)	0.0138	0.740 (0.356 to 1.535)	0.418	0.536 (0.235 to 1.222)	0.1378	0.2928	0.924 (0.667 to 1.280)	0.6357
Women	Ovarian cancer	AC	1	0.981 (0.414 to 2.325)	0.9659	0.477 (0.169 to 1.341)	0.1602	0.894 (0.383 to 2.086)	0.7958	0.776 (0.322 to 1.871)	0.5724	0.6078	1.095 (0.854 to 1.406)	0.4737
Women	Ovarian cancer	CRI-I	1	0.979 (0.413 to 2.318)	0.9614	0.476 (0.169 to 1.339)	0.1594	0.893 (0.383 to 2.083)	0.7934	0.775 (0.321 to 1.869)	0.5704	0.607	1.095 (0.854 to 1.406)	0.4737
Women	Ovarian cancer	CRI-II	1	0.743 (0.304 to 1.813)	0.5134	0.445 (0.163 to 1.212)	0.1132	0.848 (0.368 to 1.954)	0.6985	0.814 (0.360 to 1.843)	0.622	0.8401	1.120 (0.898 to 1.397)	0.3131
Women	Ovarian cancer	LCI	1	0.852 (0.353 to 2.058)	0.722	0.846 (0.362 to 1.979)	0.6995	0.527 (0.206 to 1.349)	0.1815	0.763 (0.325 to 1.793)	0.5355	0.3657	1.098 (0.972 to 1.239)	0.1317
Women	Ovarian cancer	THDL	1	0.606 (0.264 to 1.392)	0.2376	0.249 (0.082 to 0.753)	0.0138	0.740 (0.356 to 1.535)	0.418	0.536 (0.235 to 1.222)	0.1378	0.2928	1.027 (0.755 to 1.395)	0.867
Men	Overall cancer	HDL-C	1	1.019 (0.929 to 1.119)	0.6866	0.975 (0.882 to 1.077)	0.6157	0.982 (0.891 to 1.083)	0.722	1.043 (0.945 to 1.150)	0.4049	0.6958	1.017 (0.987 to 1.049)	0.2743
Men	Overall cancer	LDL-C	1	0.985 (0.891 to 1.089)	0.7699	1.037 (0.940 to 1.145)	0.4641	1.043 (0.945 to 1.151)	0.4034	1.036 (0.939 to 1.143)	0.4763	0.2558	1.008 (0.977 to 1.039)	0.628
Men	Overall cancer	TG	1	0.999 (0.906 to 1.102)	0.9858	0.957 (0.867 to 1.057)	0.3907	0.961 (0.869 to 1.063)	0.4357	0.965 (0.869 to 1.071)	0.5046	0.3559	0.982 (0.950 to 1.015)	0.2854
Men	Overall cancer	TC	1	0.957 (0.866 to 1.058)	0.3884	1.055 (0.956 to 1.163)	0.2889	1.045 (0.947 to 1.153)	0.3813	1.008 (0.913 to 1.114)	0.8694	0.3606	1.008 (0.978 to 1.040)	0.5959
Men	Overall cancer	NONHDL	1	0.994 (0.898 to 1.099)	0.9007	1.071 (0.969 to 1.182)	0.179	1.064 (0.964 to 1.175)	0.2193	1.030 (0.931 to 1.140)	0.5656	0.2699	1.003 (0.972 to 1.035)	0.8416
Men	Overall cancer	AIP	1	1.055 (0.957 to 1.163)	0.2826	0.932 (0.843 to 1.032)	0.1754	1.017 (0.920 to 1.125)	0.7396	0.964 (0.868 to 1.071)	0.4934	0.3495	0.984 (0.951 to 1.017)	0.3367
Men	Overall cancer	AC	1	0.949 (0.859 to 1.049)	0.3049	0.957 (0.866 to 1.057)	0.3851	1.016 (0.920 to 1.122)	0.7497	0.966 (0.873 to 1.069)	0.5020	0.9864	0.991 (0.960 to 1.023)	0.5835
Men	Overall cancer	CRI-I	1	0.949 (0.859 to 1.049)	0.3050	0.957 (0.866 to 1.057)	0.3851	1.016 (0.920 to 1.122)	0.7497	0.966 (0.873 to 1.069)	0.5020	0.9864	0.991 (0.960 to 1.023)	0.5835
Men	Overall cancer	CRI-II	1	0.870 (0.786 to 0.962)	0.0067	0.966 (0.875 to 1.066)	0.4917	1.019 (0.925 to 1.122)	0.7088	0.977 (0.886 to 1.078)	0.6479	0.3264	0.995 (0.965 to 1.027)	0.7692
Men	Overall cancer	LCI	1	1.025 (0.927 to 1.133)	0.6293	1.030 (0.932 to 1.139)	0.5622	1.030 (0.930 to 1.140)	0.5721	0.975 (0.877 to 1.084)	0.6377	0.6816	0.987 (0.951 to 1.024)	0.4865
Men	Overall cancer	THDL	1	1.055 (0.957 to 1.163)	0.2826	0.932 (0.843 to 1.032)	0.1754	1.017 (0.920 to 1.125)	0.7396	0.964 (0.868 to 1.071)	0.4934	0.3495	0.981 (0.949 to 1.016)	0.2822
Men	Overall cancer*	HDL-C	1	0.995 (0.897 to 1.103)	0.9197	1.002 (0.897 to 1.119)	0.9728	0.996 (0.895 to 1.109)	0.9476	1.054 (0.946 to 1.173)	0.3402	0.3995	1.026 (0.993 to 1.060)	0.1301
Men	Overall cancer*	LDL-C	1	1.004 (0.898 to 1.123)	0.9409	1.041 (0.933 to 1.161)	0.4711	1.046 (0.938 to 1.167)	0.4168	1.053 (0.945 to 1.173)	0.3529	0.2480	1.013 (0.979 to 1.048)	0.4504
Men	Overall cancer*	TG	1	0.988 (0.887 to 1.100)	0.8227	0.934 (0.837 to 1.042)	0.2225	0.932 (0.834 to 1.042)	0.2145	0.926 (0.825 to 1.040)	0.1957	0.1109	0.969 (0.934 to 1.006)	0.0993
Men	Overall cancer*	TC	1	0.971 (0.869 to 1.084)	0.6015	1.042 (0.935 to 1.163)	0.4556	1.035 (0.928 to 1.155)	0.5331	1.029 (0.922 to 1.148)	0.6061	0.3312	1.014 (0.980 to 1.050)	0.4202
Men	Overall cancer*	NONHDL	1	1.003 (0.898 to 1.121)	0.9566	1.059 (0.949 to 1.183)	0.3051	1.034 (0.926 to 1.155)	0.5491	1.051 (0.940 to 1.175)	0.3812	0.3090	1.006 (0.972 to 1.042)	0.7296

Gender	Cancer type	Lipid index	1st quintile	2nd quintile		3rd quintile		4th quintile		5th quintile		p-value for trend	Per 1 SD increase	
				aHR (95% CI)	p-value	aHR (95% CI)	p-value	aHR (95% CI)	p-value	aHR (95% CI)	p-value		aHR (95% CI)	p-value
Men	Overall cancer*	AIP	1	1.047 (0.941 to 1.165)	0.402	0.891 (0.797 to 0.995)	0.0413	0.978 (0.875 to 1.092)	0.6912	0.915 (0.815 to 1.028)	0.1342	0.0611	0.969 (0.933 to 1.005)	0.0937
Men	Overall cancer*	AC	1	0.937 (0.839 to 1.046)	0.2441	0.938 (0.840 to 1.048)	0.2595	1.000 (0.896 to 1.115)	0.9934	0.946 (0.847 to 1.057)	0.3298	0.7391	0.989 (0.954 to 1.024)	0.5269
Men	Overall cancer*	CRI-I	1	0.937 (0.839 to 1.046)	0.2441	0.938 (0.840 to 1.048)	0.2595	1.000 (0.896 to 1.115)	0.9934	0.946 (0.847 to 1.057)	0.3298	0.7391	0.989 (0.954 to 1.024)	0.5269
Men	Overall cancer*	CRI-II	1	0.869 (0.778 to 0.972)	0.0138	0.921 (0.825 to 1.028)	0.1419	1.033 (0.929 to 1.149)	0.5461	0.956 (0.858 to 1.065)	0.4102	0.5086	0.995 (0.961 to 1.030)	0.7647
Men	Overall cancer*	LCI	1	1.043 (0.934 to 1.165)	0.45	1.015 (0.908 to 1.135)	0.7883	1.018 (0.910 to 1.139)	0.7589	0.970 (0.862 to 1.091)	0.6069	0.4935	0.978 (0.938 to 1.019)	0.2867
Men	Overall cancer*	THDL	1	1.047 (0.941 to 1.165)	0.402	0.891 (0.797 to 0.995)	0.0413	0.978 (0.875 to 1.092)	0.6912	0.915 (0.815 to 1.028)	0.1342	0.0611	0.969 (0.932 to 1.007)	0.1093
Men	Lung cancer	HDL-C	1	1.084 (0.791 to 1.485)	0.6168	0.943 (0.659 to 1.350)	0.7479	1.098 (0.789 to 1.527)	0.5801	1.293 (0.936 to 1.786)	0.1195	0.1639	1.058 (0.970 to 1.154)	0.2031
Men	Lung cancer	LDL-C	1	1.023 (0.730 to 1.434)	0.8943	0.918 (0.651 to 1.295)	0.625	1.165 (0.843 to 1.609)	0.356	1.091 (0.785 to 1.515)	0.6044	0.4056	1.047 (0.943 to 1.162)	0.3931
Men	Lung cancer	TG	1	1.311 (0.953 to 1.804)	0.096	1.055 (0.752 to 1.482)	0.7558	0.959 (0.671 to 1.371)	0.8192	1.099 (0.765 to 1.580)	0.6095	0.6825	0.984 (0.874 to 1.109)	0.7969
Men	Lung cancer	TC	1	1.402 (1.003 to 1.959)	0.0481	1.212 (0.853 to 1.723)	0.2838	1.338 (0.953 to 1.879)	0.0924	1.170 (0.823 to 1.663)	0.3826	0.5404	1.047 (0.943 to 1.162)	0.3879
Men	Lung cancer	NONHDL	1	1.158 (0.821 to 1.631)	0.4033	1.433 (1.026 to 2.002)	0.0349	1.182 (0.840 to 1.661)	0.3372	1.122 (0.788 to 1.599)	0.5235	0.5442	1.026 (0.925 to 1.139)	0.6296
Men	Lung cancer	AIP	1	1.205 (0.885 to 1.641)	0.237	0.833 (0.589 to 1.179)	0.3037	0.913 (0.648 to 1.286)	0.6039	0.930 (0.650 to 1.333)	0.6941	0.2721	0.958 (0.854 to 1.075)	0.467
Men	Lung cancer	AC	1	1.052 (0.752 to 1.470)	0.7685	1.241 (0.893 to 1.724)	0.1984	1.215 (0.874 to 1.687)	0.246	0.828 (0.578 to 1.186)	0.3033	0.6086	0.961 (0.861 to 1.071)	0.4713
Men	Lung cancer	CRI-I	1	1.052 (0.752 to 1.470)	0.7685	1.241 (0.893 to 1.724)	0.1984	1.215 (0.874 to 1.687)	0.246	0.828 (0.578 to 1.186)	0.3033	0.6086	0.961 (0.861 to 1.071)	0.4713
Men	Lung cancer	CRI-II	1	0.933 (0.663 to 1.314)	0.6931	1.058 (0.758 to 1.478)	0.7394	1.278 (0.933 to 1.752)	0.1263	0.896 (0.636 to 1.261)	0.5281	0.748	0.979 (0.880 to 1.090)	0.7027
Men	Lung cancer	LCI	1	1.152 (0.821 to 1.618)	0.4122	1.333 (0.959 to 1.853)	0.0867	1.102 (0.774 to 1.569)	0.5909	0.998 (0.689 to 1.444)	0.9903	0.883	0.977 (0.855 to 1.115)	0.7271
Men	Lung cancer	THDL	1	1.205 (0.885 to 1.641)	0.237	0.833 (0.589 to 1.179)	0.3037	0.913 (0.648 to 1.286)	0.6039	0.930 (0.650 to 1.333)	0.6941	0.2721	0.962 (0.850 to 1.088)	0.5366
Men	Colon cancer	HDL-C	1	0.734 (0.491 to 1.097)	0.1314	0.840 (0.549 to 1.287)	0.4239	0.999 (0.677 to 1.475)	0.9968	0.818 (0.538 to 1.243)	0.3461	0.8009	0.989 (0.868 to 1.127)	0.8686
Men	Colon cancer	LDL-C	1	1.488 (0.960 to 2.306)	0.0756	1.106 (0.695 to 1.762)	0.6699	1.298 (0.830 to 2.031)	0.2527	1.599 (1.040 to 2.458)	0.0325	0.1029	1.150 (1.006 to 1.314)	0.0401
Men	Colon cancer	TG	1	2.460 (1.483 to 4.081)	<0.001	2.720 (1.638 to 4.517)	<0.001	1.706 (0.997 to 2.917)	0.0512	2.158 (1.252 to 3.720)	0.0056	0.1478	1.053 (0.928 to 1.194)	0.4207
Men	Colon cancer	TC	1	1.360 (0.869 to 2.128)	0.1791	1.193 (0.751 to 1.895)	0.4545	1.660 (1.077 to 2.559)	0.0217	1.487 (0.953 to 2.320)	0.0804	0.0472	1.200 (1.057 to 1.362)	0.0049
Men	Colon cancer	NONHDL	1	1.323 (0.838 to 2.088)	0.2294	1.331 (0.840 to 2.108)	0.2229	1.434 (0.918 to 2.239)	0.113	1.669 (1.070 to 2.602)	0.0239	0.0287	1.192 (1.048 to 1.357)	0.0076
Men	Colon cancer	AIP	1	1.580 (1.001 to 2.495)	0.0496	1.699 (1.080 to 2.671)	0.0217	1.135 (0.693 to 1.858)	0.6146	1.559 (0.957 to 2.539)	0.0746	0.3991	1.088 (0.951 to 1.246)	0.2201
Men	Colon cancer	AC	1	1.147 (0.747 to 1.763)	0.5302	0.936 (0.598 to 1.466)	0.774	1.093 (0.710 to 1.683)	0.686	1.332 (0.872 to 2.037)	0.1848	0.2609	1.142 (1.012 to 1.290)	0.0319
Men	Colon cancer	CRI-I	1	1.147 (0.747 to 1.763)	0.5302	0.936 (0.598 to 1.466)	0.774	1.093 (0.710 to 1.683)	0.686	1.332 (0.872 to 2.037)	0.1848	0.2609	1.142 (1.012 to 1.290)	0.0319

Gender	Cancer type	Lipid index	1st quintile	2nd quintile		3rd quintile		4th quintile		5th quintile		p-value for trend	Per 1 SD increase	
				aHR (95% CI)	p-value	aHR (95% CI)	p-value	aHR (95% CI)	p-value	aHR (95% CI)	p-value		aHR (95% CI)	p-value
Men	Colon cancer	CRI-II	1	1.115 (0.727 to 1.710)	0.6177	0.855 (0.541 to 1.352)	0.5029	1.212 (0.801 to 1.834)	0.362	1.291 (0.851 to 1.960)	0.2302	0.201	1.132 (0.998 to 1.283)	0.0534
Men	Colon cancer	LCI	1	1.766 (1.074 to 2.905)	0.025	2.058 (1.269 to 3.339)	0.0035	1.517 (0.909 to 2.533)	0.111	2.307 (1.398 to 3.807)	0.0011	0.0094	1.068 (0.989 to 1.153)	0.0955
Men	Colon cancer	THDL	1	1.580 (1.001 to 2.495)	0.0496	1.699 (1.080 to 2.671)	0.0217	1.135 (0.693 to 1.858)	0.6146	1.559 (0.957 to 2.539)	0.0746	0.3991	1.034 (0.918 to 1.165)	0.581
Men	Rectal cancer	HDL-C	1	1.482 (0.939 to 2.337)	0.0908	1.085 (0.661 to 1.781)	0.7473	1.198 (0.738 to 1.945)	0.4655	1.387 (0.853 to 2.254)	0.1872	0.4697	1.032 (0.908 to 1.172)	0.6331
Men	Rectal cancer	LDL-C	1	0.871 (0.552 to 1.373)	0.5515	1.103 (0.717 to 1.695)	0.6556	0.853 (0.539 to 1.349)	0.4955	0.900 (0.570 to 1.420)	0.6506	0.6528	0.951 (0.819 to 1.105)	0.5133
Men	Rectal cancer	TG	1	0.931 (0.576 to 1.506)	0.7715	1.088 (0.681 to 1.738)	0.7242	1.284 (0.809 to 2.037)	0.2887	1.239 (0.763 to 2.013)	0.3866	0.1738	1.068 (0.939 to 1.214)	0.3146
Men	Rectal cancer	TC	1	1.013 (0.654 to 1.567)	0.9555	0.872 (0.551 to 1.380)	0.5596	0.708 (0.433 to 1.156)	0.1676	1.119 (0.721 to 1.735)	0.617	0.9059	1.007 (0.868 to 1.168)	0.9268
Men	Rectal cancer	NONHDL	1	0.980 (0.627 to 1.531)	0.9287	1.078 (0.688 to 1.690)	0.7423	0.597 (0.351 to 1.016)	0.0571	1.189 (0.757 to 1.867)	0.4525	0.9884	0.998 (0.858 to 1.162)	0.9806
Men	Rectal cancer	AIP	1	0.884 (0.559 to 1.400)	0.6005	0.948 (0.594 to 1.515)	0.8244	1.147 (0.728 to 1.806)	0.5544	1.101 (0.682 to 1.777)	0.6931	0.4154	1.068 (0.913 to 1.248)	0.4115
Men	Rectal cancer	AC	1	1.567 (1.027 to 2.392)	0.0373	0.726 (0.432 to 1.221)	0.2276	0.874 (0.524 to 1.457)	0.6056	1.087 (0.670 to 1.765)	0.7348	0.3703	0.962 (0.825 to 1.121)	0.6176
Men	Rectal cancer	CRI-I	1	1.567 (1.027 to 2.392)	0.0373	0.726 (0.432 to 1.221)	0.2276	0.874 (0.524 to 1.457)	0.6056	1.087 (0.670 to 1.765)	0.7348	0.3703	0.962 (0.825 to 1.121)	0.6176
Men	Rectal cancer	CRI-II	1	1.197 (0.773 to 1.854)	0.4192	1.089 (0.696 to 1.705)	0.7079	0.730 (0.440 to 1.210)	0.2218	1.010 (0.631 to 1.617)	0.9672	0.3945	0.930 (0.794 to 1.089)	0.3675
Men	Rectal cancer	LCI	1	1.457 (0.913 to 2.324)	0.1146	1.354 (0.832 to 2.204)	0.2226	1.001 (0.590 to 1.699)	0.9959	1.379 (0.819 to 2.321)	0.2267	0.7036	0.986 (0.863 to 1.126)	0.8335
Men	Rectal cancer	THDL	1	0.884 (0.559 to 1.400)	0.6005	0.948 (0.594 to 1.515)	0.8244	1.147 (0.728 to 1.806)	0.5544	1.101 (0.682 to 1.777)	0.6931	0.4154	1.031 (0.906 to 1.173)	0.6407
Men	Stomach cancer	HDL-C	1	0.797 (0.635 to 0.999)	0.0487	0.896 (0.709 to 1.133)	0.3581	0.727 (0.572 to 0.924)	0.0092	0.883 (0.699 to 1.115)	0.294	0.1915	0.948 (0.877 to 1.025)	0.1793
Men	Stomach cancer	LDL-C	1	0.807 (0.629 to 1.034)	0.0902	0.773 (0.603 to 0.992)	0.0427	1.025 (0.813 to 1.293)	0.8322	1.038 (0.825 to 1.306)	0.7502	0.2377	1.043 (0.965 to 1.128)	0.2861
Men	Stomach cancer	TG	1	1.018 (0.798 to 1.299)	0.887	0.861 (0.667 to 1.110)	0.2479	1.018 (0.793 to 1.305)	0.89	1.091 (0.848 to 1.403)	0.4982	0.5239	1.051 (0.978 to 1.129)	0.1778
Men	Stomach cancer	TC	1	0.770 (0.596 to 0.993)	0.0443	1.014 (0.799 to 1.288)	0.9062	0.880 (0.688 to 1.125)	0.3076	1.164 (0.924 to 1.468)	0.1972	0.097	1.061 (0.982 to 1.148)	0.1341
Men	Stomach cancer	NONHDL	1	0.811 (0.626 to 1.050)	0.1112	1.032 (0.808 to 1.319)	0.801	0.979 (0.767 to 1.251)	0.8673	1.202 (0.945 to 1.527)	0.1334	0.0425	1.075 (0.995 to 1.161)	0.0678
Men	Stomach cancer	AIP	1	1.147 (0.896 to 1.469)	0.2774	0.921 (0.710 to 1.195)	0.5367	1.193 (0.929 to 1.532)	0.1676	1.152 (0.891 to 1.489)	0.2817	0.2748	1.061 (0.977 to 1.152)	0.1597
Men	Stomach cancer	AC	1	0.998 (0.774 to 1.286)	0.9848	1.056 (0.819 to 1.363)	0.6732	1.086 (0.844 to 1.397)	0.5224	1.229 (0.959 to 1.576)	0.1035	0.0759	1.071 (0.998 to 1.150)	0.0559
Men	Stomach cancer	CRI-I	1	0.998 (0.774 to 1.286)	0.9848	1.056 (0.819 to 1.363)	0.6732	1.086 (0.844 to 1.397)	0.5224	1.229 (0.959 to 1.576)	0.1035	0.0759	1.071 (0.998 to 1.150)	0.0559
Men	Stomach cancer	CRI-II	1	0.846 (0.658 to 1.089)	0.1947	0.816 (0.632 to 1.052)	0.1169	1.072 (0.844 to 1.362)	0.5662	1.151 (0.910 to 1.455)	0.241	0.0555	1.054 (0.980 to 1.133)	0.1548
Men	Stomach cancer	LCI	1	1.021 (0.793 to 1.315)	0.8723	0.997 (0.772 to 1.287)	0.9795	1.010 (0.781 to 1.306)	0.9397	1.206 (0.933 to 1.558)	0.1518	0.1969	1.030 (0.973 to 1.090)	0.3108
Men	Stomach cancer	THDL	1	1.147 (0.896 to 1.469)	0.2774	0.921 (0.710 to 1.195)	0.5367	1.193 (0.929 to 1.532)	0.1676	1.152 (0.891 to 1.489)	0.2817	0.2748	1.052 (0.984 to 1.124)	0.1378

Gender	Cancer type	Lipid index	1st quintile	2nd quintile		3rd quintile		4th quintile		5th quintile		p-value for trend	Per 1 SD increase	
				aHR (95% CI)	p-value	aHR (95% CI)	p-value	aHR (95% CI)	p-value	aHR (95% CI)	p-value		aHR (95% CI)	p-value
Men	Liver cancer	HDL-C	1	1.356 (0.925 to 1.988)	0.1182	0.872 (0.551 to 1.378)	0.5564	1.244 (0.830 to 1.866)	0.29	1.516 (1.016 to 2.263)	0.0416	0.1232	1.082 (0.959 to 1.221)	0.1983
Men	Liver cancer	LDL-C	1	1.059 (0.731 to 1.536)	0.7613	0.830 (0.561 to 1.227)	0.3506	0.755 (0.506 to 1.125)	0.1673	0.610 (0.402 to 0.927)	0.0206	0.0043	0.860 (0.759 to 0.975)	0.0188
Men	Liver cancer	TG	1	0.562 (0.396 to 0.796)	0.0012	0.376 (0.255 to 0.553)	<0.001	0.281 (0.184 to 0.428)	<0.001	0.254 (0.163 to 0.396)	<0.001	<0.001	0.626 (0.477 to 0.820)	<0.001
Men	Liver cancer	TC	1	0.903 (0.625 to 1.305)	0.587	0.848 (0.582 to 1.235)	0.3904	0.615 (0.409 to 0.926)	0.0199	0.479 (0.311 to 0.736)	<0.001	<0.001	0.765 (0.673 to 0.869)	<0.001
Men	Liver cancer	NONHDL	1	0.714 (0.496 to 1.030)	0.0717	0.731 (0.505 to 1.056)	0.0949	0.431 (0.283 to 0.656)	<0.001	0.445 (0.295 to 0.671)	<0.001	<0.001	0.747 (0.649 to 0.861)	<0.001
Men	Liver cancer	AIP	1	0.728 (0.514 to 1.032)	0.0743	0.428 (0.285 to 0.641)	<0.001	0.338 (0.222 to 0.516)	<0.001	0.308 (0.197 to 0.482)	<0.001	<0.001	0.650 (0.553 to 0.763)	<0.001
Men	Liver cancer	AC	1	0.779 (0.540 to 1.123)	0.1812	0.550 (0.367 to 0.823)	0.0037	0.508 (0.337 to 0.764)	0.0012	0.512 (0.342 to 0.766)	0.0011	<0.001	0.818 (0.680 to 0.983)	0.0321
Men	Liver cancer	CRI-I	1	0.779 (0.540 to 1.123)	0.1812	0.550 (0.367 to 0.823)	0.0037	0.508 (0.337 to 0.764)	0.0012	0.512 (0.342 to 0.766)	0.0011	<0.001	0.818 (0.680 to 0.983)	0.0321
Men	Liver cancer	CRI-II	1	0.815 (0.557 to 1.190)	0.2889	0.723 (0.487 to 1.072)	0.1064	0.607 (0.405 to 0.909)	0.0155	0.679 (0.460 to 1.000)	0.0501	0.0216	0.876 (0.757 to 1.013)	0.0738
Men	Liver cancer	LCI	1	0.656 (0.464 to 0.928)	0.0172	0.400 (0.269 to 0.593)	<0.001	0.324 (0.213 to 0.493)	<0.001	0.311 (0.204 to 0.474)	<0.001	<0.001	0.694 (0.494 to 0.976)	0.036
Men	Liver cancer	THDL	1	0.728 (0.514 to 1.032)	0.0743	0.428 (0.285 to 0.641)	<0.001	0.338 (0.222 to 0.516)	<0.001	0.308 (0.197 to 0.482)	<0.001	<0.001	0.724 (0.518 to 1.012)	0.0584
Men	Bladder cancer	HDL-C	1	1.008 (0.564 to 1.802)	0.9784	0.727 (0.353 to 1.496)	0.3863	1.524 (0.872 to 2.664)	0.1389	0.650 (0.327 to 1.291)	0.2188	0.7824	0.921 (0.787 to 1.077)	0.3011
Men	Bladder cancer	LDL-C	1	0.927 (0.460 to 1.866)	0.8317	1.076 (0.559 to 2.069)	0.827	1.139 (0.601 to 2.160)	0.6892	1.231 (0.655 to 2.313)	0.5194	0.3834	1.032 (0.855 to 1.247)	0.7423
Men	Bladder cancer	TG	1	1.109 (0.534 to 2.301)	0.7818	1.561 (0.782 to 3.118)	0.2068	1.433 (0.691 to 2.970)	0.3331	1.905 (0.941 to 3.858)	0.0734	0.0555	1.125 (0.977 to 1.295)	0.1011
Men	Bladder cancer	TC	1	1.021 (0.524 to 1.988)	0.952	0.884 (0.439 to 1.779)	0.7294	1.480 (0.803 to 2.726)	0.2085	1.352 (0.720 to 2.540)	0.348	0.1666	1.089 (0.903 to 1.312)	0.3716
Men	Bladder cancer	NONHDL	1	1.017 (0.502 to 2.060)	0.9636	1.078 (0.535 to 2.172)	0.8339	1.406 (0.731 to 2.706)	0.3074	1.653 (0.861 to 3.174)	0.1312	0.0687	1.113 (0.925 to 1.339)	0.2567
Men	Bladder cancer	AIP	1	1.386 (0.667 to 2.877)	0.3815	1.665 (0.813 to 3.412)	0.1637	1.605 (0.768 to 3.353)	0.2079	1.804 (0.860 to 3.784)	0.1187	0.1239	1.184 (0.963 to 1.455)	0.1088
Men	Bladder cancer	AC	1	0.949 (0.457 to 1.969)	0.8881	1.462 (0.752 to 2.842)	0.2633	1.214 (0.610 to 2.419)	0.5808	1.489 (0.771 to 2.879)	0.2361	0.1796	1.066 (0.907 to 1.252)	0.4403
Men	Bladder cancer	CRI-I	1	0.949 (0.457 to 1.969)	0.8881	1.462 (0.752 to 2.842)	0.2633	1.214 (0.610 to 2.419)	0.5808	1.489 (0.771 to 2.879)	0.2361	0.1796	1.066 (0.907 to 1.252)	0.4403
Men	Bladder cancer	CRI-II	1	1.036 (0.520 to 2.063)	0.9194	0.997 (0.497 to 2.000)	0.993	1.437 (0.760 to 2.718)	0.2645	1.113 (0.576 to 2.152)	0.7492	0.45	1.018 (0.857 to 1.208)	0.8397
Men	Bladder cancer	LCI	1	1.111 (0.527 to 2.345)	0.7821	1.138 (0.543 to 2.386)	0.7325	1.873 (0.942 to 3.723)	0.0733	1.567 (0.746 to 3.292)	0.2355	0.0685	1.033 (0.909 to 1.174)	0.6189
Men	Bladder cancer	THDL	1	1.386 (0.667 to 2.877)	0.3815	1.665 (0.813 to 3.412)	0.1637	1.605 (0.768 to 3.353)	0.2079	1.804 (0.860 to 3.784)	0.1187	0.1239	1.096 (0.964 to 1.246)	0.1598
Men	Prostate cancer	HDL-C	1	1.098 (0.842 to 1.432)	0.4914	1.096 (0.819 to 1.466)	0.5387	1.019 (0.766 to 1.356)	0.8954	1.053 (0.803 to 1.381)	0.7083	0.903	1.054 (0.972 to 1.143)	0.2024
Men	Prostate cancer	LDL-C	1	1.144 (0.834 to 1.569)	0.4034	1.522 (1.134 to 2.042)	0.0051	1.342 (0.993 to 1.814)	0.0557	1.324 (0.979 to 1.790)	0.0681	0.0404	1.079 (0.991 to 1.174)	0.0798
Men	Prostate cancer	TG	1	1.078 (0.830 to 1.400)	0.5744	0.903 (0.684 to 1.193)	0.4742	0.929 (0.701 to 1.232)	0.6091	0.811 (0.594 to 1.109)	0.1891	0.1082	0.934 (0.839 to 1.041)	0.219

Gender	Cancer type	Lipid index	1st quintile	2nd quintile		3rd quintile		4th quintile		5th quintile		p-value for trend	Per 1 SD increase	
				aHR (95% CI)	p-value	aHR (95% CI)	p-value	aHR (95% CI)	p-value	aHR (95% CI)	p-value		aHR (95% CI)	p-value
Men	Prostate cancer	TC	1	0.962 (0.716 to 1.294)	0.7999	1.150 (0.863 to 1.533)	0.339	1.230 (0.931 to 1.624)	0.1448	1.177 (0.881 to 1.571)	0.2697	0.0757	1.069 (0.982 to 1.165)	0.1244
Men	Prostate cancer	NONHDL	1	1.193 (0.889 to 1.602)	0.239	1.221 (0.907 to 1.643)	0.1887	1.314 (0.985 to 1.751)	0.0632	1.300 (0.963 to 1.755)	0.0861	0.0627	1.050 (0.962 to 1.146)	0.2746
Men	Prostate cancer	AIP	1	1.074 (0.826 to 1.397)	0.5932	0.859 (0.648 to 1.137)	0.2875	0.991 (0.750 to 1.309)	0.9467	0.834 (0.616 to 1.129)	0.2399	0.2025	0.941 (0.856 to 1.035)	0.2106
Men	Prostate cancer	AC	1	0.780 (0.579 to 1.052)	0.1041	0.999 (0.755 to 1.322)	0.9961	1.219 (0.932 to 1.594)	0.1488	0.941 (0.704 to 1.258)	0.6805	0.3129	1.001 (0.915 to 1.096)	0.9745
Men	Prostate cancer	CRI-I	1	0.780 (0.579 to 1.052)	0.1041	0.999 (0.755 to 1.322)	0.9961	1.219 (0.932 to 1.594)	0.1488	0.941 (0.704 to 1.258)	0.6805	0.3129	1.001 (0.915 to 1.096)	0.9745
Men	Prostate cancer	CRI-II	1	0.933 (0.691 to 1.259)	0.6486	1.007 (0.750 to 1.352)	0.964	1.292 (0.981 to 1.703)	0.0685	1.087 (0.814 to 1.450)	0.5724	0.1169	1.032 (0.945 to 1.126)	0.4836
Men	Prostate cancer	LCI	1	1.281 (0.969 to 1.695)	0.0825	1.065 (0.793 to 1.430)	0.6752	1.337 (1.007 to 1.775)	0.0446	1.065 (0.776 to 1.461)	0.6967	0.5674	0.979 (0.873 to 1.098)	0.718
Men	Prostate cancer	THDL	1	1.074 (0.826 to 1.397)	0.5932	0.859 (0.648 to 1.137)	0.2875	0.991 (0.750 to 1.309)	0.9467	0.834 (0.616 to 1.129)	0.2399	0.2025	0.938 (0.840 to 1.048)	0.2573
Men	Thyroid cancer	HDL-C	1	1.111 (0.889 to 1.388)	0.3563	0.864 (0.683 to 1.094)	0.226	0.861 (0.677 to 1.095)	0.2225	0.919 (0.720 to 1.172)	0.4943	0.1167	0.950 (0.873 to 1.034)	0.2323
Men	Thyroid cancer	LDL-C	1	0.939 (0.742 to 1.188)	0.6015	1.061 (0.843 to 1.334)	0.6151	1.093 (0.869 to 1.375)	0.4466	1.010 (0.798 to 1.278)	0.9339	0.5197	1.000 (0.931 to 1.074)	0.9971
Men	Thyroid cancer	TG	1	1.121 (0.881 to 1.426)	0.3535	1.140 (0.895 to 1.452)	0.2902	1.158 (0.906 to 1.480)	0.2405	1.243 (0.967 to 1.598)	0.0899	0.1072	1.050 (0.977 to 1.129)	0.1846
Men	Thyroid cancer	TC	1	1.000 (0.787 to 1.271)	0.9975	1.190 (0.945 to 1.498)	0.1382	1.172 (0.927 to 1.481)	0.1839	1.005 (0.787 to 1.283)	0.9695	0.5379	1.002 (0.931 to 1.079)	0.9523
Men	Thyroid cancer	NONHDL	1	1.048 (0.822 to 1.336)	0.7064	1.209 (0.955 to 1.530)	0.1143	1.321 (1.043 to 1.672)	0.0208	1.032 (0.803 to 1.327)	0.8053	0.2754	1.015 (0.943 to 1.092)	0.693
Men	Thyroid cancer	AIP	1	1.165 (0.911 to 1.491)	0.2227	1.248 (0.975 to 1.598)	0.0781	1.330 (1.037 to 1.706)	0.0247	1.325 (1.025 to 1.714)	0.0318	0.0197	1.078 (0.996 to 1.166)	0.0623
Men	Thyroid cancer	AC	1	1.073 (0.842 to 1.369)	0.5693	1.177 (0.927 to 1.496)	0.1809	1.191 (0.931 to 1.523)	0.1643	1.188 (0.923 to 1.529)	0.1811	0.1231	1.026 (0.956 to 1.101)	0.4727
Men	Thyroid cancer	CRI-I	1	1.073 (0.842 to 1.369)	0.5693	1.177 (0.927 to 1.496)	0.1809	1.191 (0.931 to 1.523)	0.1643	1.188 (0.923 to 1.529)	0.1811	0.1231	1.026 (0.956 to 1.101)	0.4727
Men	Thyroid cancer	CRI-II	1	0.919 (0.720 to 1.174)	0.4995	1.265 (1.007 to 1.588)	0.0431	1.011 (0.794 to 1.289)	0.9268	1.180 (0.928 to 1.502)	0.1767	0.1229	1.018 (0.950 to 1.091)	0.6165
Men	Thyroid cancer	LCI	1	1.018 (0.800 to 1.295)	0.8862	1.209 (0.955 to 1.531)	0.1153	1.164 (0.911 to 1.488)	0.2243	1.099 (0.853 to 1.415)	0.4651	0.2816	1.032 (0.956 to 1.113)	0.4229
Men	Thyroid cancer	THDL	1	1.165 (0.911 to 1.491)	0.2227	1.248 (0.975 to 1.598)	0.0781	1.330 (1.037 to 1.706)	0.0247	1.325 (1.025 to 1.714)	0.0318	0.0197	1.048 (0.977 to 1.123)	0.1885
Men	Pancreas cacner	HDL-C	1	1.617 (0.881 to 2.969)	0.121	1.743 (0.912 to 3.334)	0.0929	1.602 (0.831 to 3.088)	0.1593	1.359 (0.673 to 2.744)	0.3926	0.3762	1.086 (0.920 to 1.282)	0.327
Men	Pancreas cacner	LDL-C	1	0.705 (0.333 to 1.496)	0.3629	1.516 (0.819 to 2.806)	0.1856	1.250 (0.662 to 2.362)	0.4915	1.272 (0.674 to 2.398)	0.4577	0.18	1.073 (0.908 to 1.269)	0.4077
Men	Pancreas cacner	TG	1	1.157 (0.597 to 2.244)	0.6657	1.166 (0.604 to 2.250)	0.6478	1.053 (0.538 to 2.062)	0.8792	1.073 (0.538 to 2.141)	0.8422	0.9883	0.933 (0.775 to 1.123)	0.4629
Men	Pancreas cacner	TC	1	0.958 (0.470 to 1.955)	0.9067	1.364 (0.691 to 2.692)	0.371	2.024 (1.091 to 3.755)	0.0254	1.048 (0.525 to 2.093)	0.8935	0.1828	1.072 (0.900 to 1.277)	0.4369
Men	Pancreas cacner	NONHDL	1	1.552 (0.771 to 3.122)	0.2183	1.239 (0.585 to 2.624)	0.5756	2.184 (1.115 to 4.278)	0.0228	1.347 (0.656 to 2.766)	0.4173	0.1985	1.044 (0.873 to 1.250)	0.6356
Men	Pancreas cacner	AIP	1	1.334 (0.698 to 2.550)	0.3831	0.994 (0.499 to 1.980)	0.9871	1.187 (0.612 to 2.300)	0.6123	0.809 (0.389 to 1.683)	0.5709	0.438	0.941 (0.769 to 1.150)	0.5501

Gender	Cancer type	Lipid index	1st quintile	2nd quintile		3rd quintile		4th quintile		5th quintile		p-value for trend	Per 1 SD increase	
				aHR (95% CI)	p-value	aHR (95% CI)	p-value	aHR (95% CI)	p-value	aHR (95% CI)	p-value		aHR (95% CI)	p-value
Men	Pancreas cacner	AC	1	0.629 (0.316 to 1.254)	0.1877	0.881 (0.467 to 1.661)	0.6947	0.735 (0.385 to 1.402)	0.3499	0.881 (0.475 to 1.636)	0.6885	0.8746	0.949 (0.786 to 1.145)	0.5845
Men	Pancreas cacner	CRI-I	1	0.629 (0.316 to 1.254)	0.1877	0.881 (0.467 to 1.661)	0.6947	0.735 (0.385 to 1.402)	0.3499	0.881 (0.475 to 1.636)	0.6885	0.8746	0.949 (0.786 to 1.145)	0.5845
Men	Pancreas cacner	CRI-II	1	0.992 (0.496 to 1.982)	0.9815	1.236 (0.643 to 2.377)	0.5254	1.262 (0.662 to 2.406)	0.4802	1.037 (0.532 to 2.021)	0.9155	0.693	0.976 (0.824 to 1.156)	0.7792
Men	Pancreas cacner	LCI	1	1.103 (0.529 to 2.299)	0.7934	1.413 (0.700 to 2.853)	0.335	1.510 (0.759 to 3.005)	0.2403	1.192 (0.564 to 2.521)	0.6457	0.4165	0.934 (0.769 to 1.134)	0.4913
Men	Pancreas cacner	THDL	1	1.334 (0.698 to 2.550)	0.3831	0.994 (0.499 to 1.980)	0.9871	1.187 (0.612 to 2.300)	0.6123	0.809 (0.389 to 1.683)	0.5709	0.438	0.887 (0.723 to 1.089)	0.2516

Note:

HDL-C: High Density Lipoprotein Cholesterol; LDL-C: Low density lipoprotein Cholesterol; TG: Triglycerides; TC: Total Cholesterol; NONHDL: Non-high-density lipoprotein Cholesterol; AIP: Atherogenic Index of plasma;

AC: Atherogenic coefficient; CRI-I: Castelli risk index -I; CRI-II: Castelli risk index-II; LCI: Lipoprotein combination index; THDL: Triglyceride HDL-C ratio.

**: Overall cancer excluded participants of breast cancer and thyroid cancer.*

Model: Cox Proportional-Hazards Model adjusted for age, body mass index, Type 2 Diabetes mellitus, hypertension, smoking history, alcohol consumption.

Table S6. R Packages

Name	Version
broom	1.0.7
compareGroups	4.8.0
data.table	1.16.0
dplyr	1.1.4
flextable	0.9.6
fst	0.9.8
fstcore	0.9.18
ggplot2	3.5.1
ggpubr	0.6.0
ggrepel	0.9.5
gmodels	2.19.1
HardyWeinberg	1.7.8
haven	2.5.4
Hmisc	5.1-3
jtools	2.2.2
lmtest	0.9-40
MASS	7.3-60.0.1
pec	2023.04.12
purrr	1.0.2
Rsolnp	1.16
sandwich	3.1-0
summarytools	1.0.1
survcompare	0.1.2
survival	3.7-0
survminer	0.4.9
timereg	2.0.5
EValue	4.1.3
